# Pr_1–*x*_Ca_*x*_MnO_3_‑Based Memristive Heterostructures: Basic Mechanisms and Applications

**DOI:** 10.1021/acs.chemrev.4c00813

**Published:** 2025-06-26

**Authors:** Max Buczek, Zoe Moos, Alexander Gutsche, Stephan Menzel, Regina Dittmann

**Affiliations:** 28334 Peter Grünberg Institut (PGI-7/10), Forschungszentrum Jülich GmbH, 52425 Jülich, Germany

## Abstract

Memristive devices are highly promising candidates for overcoming the limits of conventional nonvolatile memory, such as flash memory, due to their high scalability, low power consumption, and simple structure. Moreover, memristive devices might be employed as hardware representations of synapses in neuromorphic circuits. Heterostructures of the perovskite Pr_1–*x*_Ca_*x*_MnO_3_ (PCMO) and a tunnel oxide are a well-studied system and a famous representative of area-dependent switching in the family of valence change memory. In contrast to filamentary switching, area-dependent switching can be tuned gradually, making it highly interesting for application in neuromorphic circuits. Further, PCMO-based devices are considered a persistent memory for DRAM replacement. This review discusses PCMO as a material and its properties, the types and aspects of memristive heterostructures based on PCMO, and different switching mechanisms. Finally, it provides an overview of the use of PCMO-based devices in neuromorphic applications and industrial activities on PCMO-based devices for memory.

## Graphical abstract

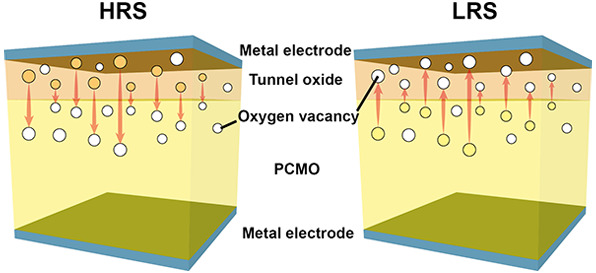

## 1 Introduction

Due to their high scalability and simple structure, memristive devices are promising candidates as a storage class memory or as a competing technology to overcome the limitations of conventional nonvolatile memories such as flash memory. They are already being used embedded memory due to their ability to integrate BEOL and their low power consumption. In addition, memristive devices can be used as hardware representations of synapses in neuromorphic circuits.^1–3^

One of the most common types of memristive device is based on the valence change mechanism (VCM), which is caused by the movement of oxygen vacancies in mixed conducting oxide thin films or heterostructures. The switching behavior of VCM devices can be distinguished between a resistance change along a filament or a resistance change across the entire device interface. Filamentary switching is stochastic because the movement of the vacancies is highly temperature accelerated by the Joule heating of the filament. In contrast, the kinetics of area-dependent switching can be tuned gradually, making it highly interesting for application in neuromorphic circuits.^4^

Stacks of the perovskite Pr_1–*x*_Ca_*x*_MnO_3_ (PCMO) and an insulating oxide are an intensively studied material system for area-dependent switching. This review gives an overview of the broad field of studies, from aspects of the material system to the application of its devices.^5^

Section 2 therefore provides a broad overview of the material properties of PCMO. The section starts with the atomic and electronic structure and the resulting electronic transport properties, which are important for device modeling. The effect of oxygen vacancies on carrier density, mobility and band gap is then discussed, providing a basis for explaining the effect of oxygen vacancies during resistive switching. The last two subsections of section 2 discuss studies of the work function and permittivity of PCMO to evaluate the choice of parameters for physical models.

Section 3 then moves from PCMO as a material to PCMO in a stack. The section starts with a classification of the different material choices to achieve a switching interface. The section continues with the effect of the thermodynamic potential of the material on the interface and interfacial oxide formation. The next section discusses the electronic potential at the interface and reviews the studies on the formation of an interfacial space charge region. The final section discusses models for electronic transport through the interfacial region.

After describing the stacks and the interfacial region, section 4 discusses switching by oxygen vacancy movement across the interface. The section begins with a general consideration of area dependent switching. The different types of switching mechanisms proposed in the literature are then introduced. Roughly divided into mechanisms that consider the effect of oxygen vacancies in the PCMO and mechanisms that consider the effect on the tunnel oxide, the next two sections give a detailed analysis of these mechanisms. This is followed by the kinetics of vacancy movement and the stability of the resistive states. The section ends with the reliability of the switching process, starting with endurance and followed by other reliability criteria.

Section 5 deals with the application of PCMO-based devices. The first section covers the engineering of potentiation and depression curves for use in artificial neural networks. The section ends with the use of potentiation and depression for time-dependent spiking neural networks. The second section discusses the integration of PCMO-based devices into crossbar arrays. This section starts with the difficulties of CMOS compatible PCMO deposition, then discusses the issues of sneak path current and readout concepts of memory arrays. The section closes with studies of PCMO-based crossbar arrays and hardware as well as simulation-based performance in artificial neural networks. The final section gives an overview of industrial applications and industrial activities of PCMO-based memories.

The final section provides a brief outlook by identifying critical and open research questions in the field. By thoroughly covering all aspects of PCMO-based devices, this review provides a full overview of the material system and its applications compared to other recent reviews.^6–8^ It will help the research community to identify open questions, stimulate numerical simulation and modulation, and place PCMO as a material system in the rapidly growing field of neuromorphic computing.

## 2 Basics of PCMO

### 2.1 Crystal Structure, Phase Diagram, and Magneto Resistance

Pr_1–*x*_Ca_*x*_MnO_3_ has an orthorhombic distorted perovskite ABO_3_ structure with Pr and Ca sharing the A site and Manganese on the B site over the whole doping region. The ionic radii of Pr^3+^ (137 pm) and Ca^2+^ (134 pm) under 12-fold coordination^9^ are similar, which leads to a stress-free homogeneous distribution on the A side places. Pr_1–*x*_Ca_*x*_MnO_3_ has a small bandwidth^10^ compared to other rear earth manganites such as La_1–*x*_Sr_*x*_MnO_3_, because of the small ion radii of Pr^3+^ and Ca^2+^ compared to other A-site atoms. For LSMO, Sr is larger than Ca since it is in period 5, compared to period 4 Ca, and La is larger since it has two atomic numbers less in the f-block as Pr.^11^ The average small ion radius *r*_A_ on the A site, comparing to the B site *r*_B_, leads to a decrease in the Goldschmidt tolerance factor

2.1 $$t=\frac{1}{\sqrt{2}}\frac{r_{A}+r_{O}}{r_{B}+r_{O}}$$

Here *r*_O_ is the radius of the oxygen anion. A Goldschmidt factor smaller 1 means the ionic distances in the Mn/O plane of the perovskite structure are larger than the distances in the Pr/Ca/O plane. Therefore, the crystal responds by a bending of the interplanar Mn–O–Mn bond angle away from 180°. The octahedral tilting changes the ideal cubic perovskite structure of PCMO to an orthorhombic symmetry over its entire doping range^9^ and increases the unit cell to account for the octahedral tilting (Figure 1b). Reducing the bond angle reduces the transfer integral of the Manganese e_g_ electron across the Mn–O–Mn bond.^10,11^ This localization causes a small bandwidth of PCMO. This strong influence of the A ion on the resistance can also be seen in Figure 11a. Experimentally, intense external hydrostatic pressure can reduce this buckling of the Mn–O–Mn bonds and increase the bandwidth.^12^

**Figure 1:**
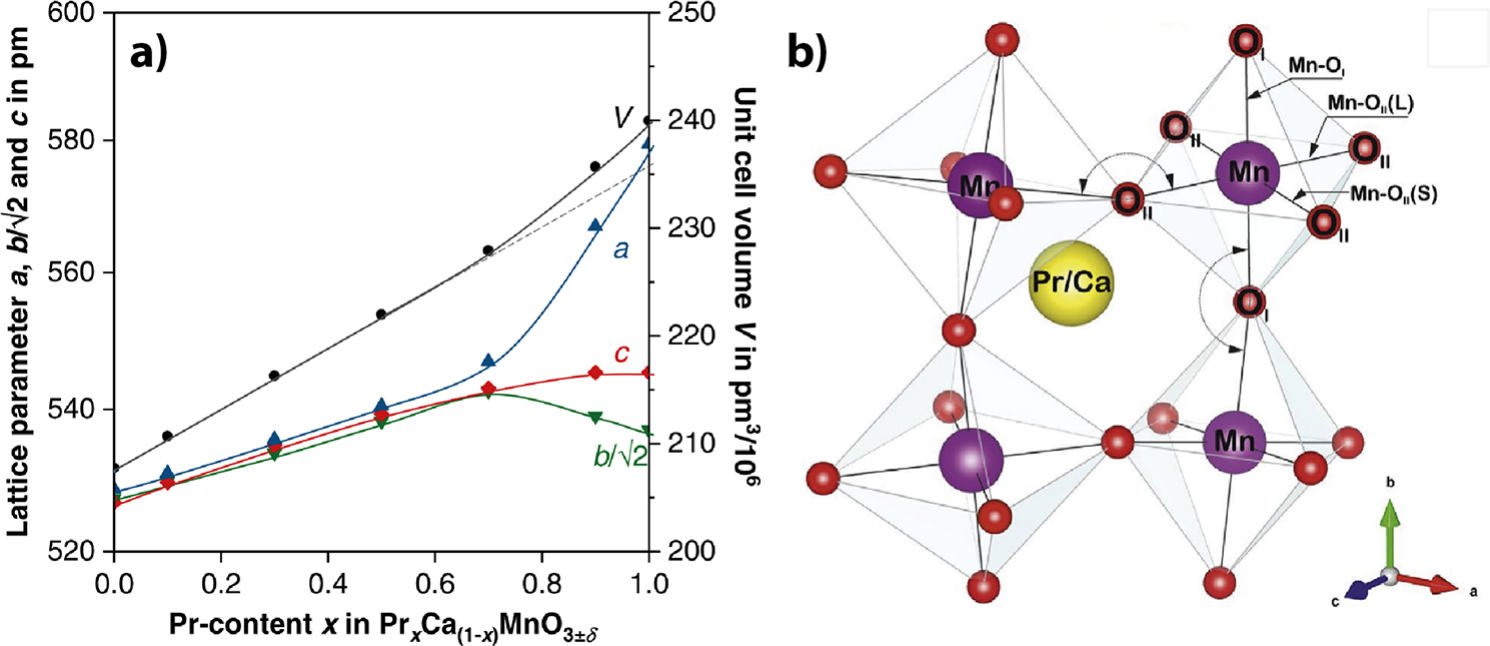
a) Pseudo cubic lattice parameter of PCMO in dependence of the doping. Adapted and reprinted with permission from ref 9. Copyright 2022 by Elsevier. b) Fragment of the PCMO unit cell to show the octahedral tilting. Adapted and reprinted with permission from ref 14. Copyright 2018 by Elsevier.

Doping with Ca increases the amount of Mn^4+^ and decreases the ionic radius on the B site. The ionic radius of Mn in a 6-fold coordination is 64.5 pm, ranging from Mn^3+^ at high spin coordination to 53 pm for Mn^4+^.^13^ The small A site radius of Ca is compensated by reducing the average B site radii. Therefore, the pseudocubic lattice parameters *a*,*b*, and *c* decrease linearly, as can be seen in Figure 1a for Ca content *x* > 0.3. This can be regarded as a solid solution according to a modified version of Vegard’s law where, here, the linear combination of the lattice constant does not refer to the doping but to the doping controlled average ionic manganese radii. CaMnO_3_ and PCMO have an orthorhombic symmetry of the space group *Pmna* up to a Ca doping of *x* ≥ 0.3. For low Ca doping *x* < 0.3, the higher ratio of Mn^3+^ induces a symmetry reduction from *Pmna* toward Pbnm.^9^

The Manganite family has been the subject of extensive research due to its manifestation of colossal magnetoresistance (CMR).^10^ An effect where the application of a magnetic field *H* in the order of a few Tesla leads to a substantial reduction of the resistance *R*, where (*R*(*H*) – *R*(0))/*R*(0) can go close to 100%.

The manifestation of colossal magnetoresistance (CMR) in manganites is tightly connected with the double exchange, a mechanism according to an increase of the delocalization of the e_g_ electron for parallel spin alignment of neighboring mixed valence Mn^3+/4+^ ions. The probability of electron transfer Mn^3+^–O-Mn^4+^ goes by cos­(Θ/2) where Θ is the angle between the spins of the neighboring Mn ions under the assumption of strong Hund coupling.^15^ This mechanism combines ferromagnetic ordering with metallic conduction, leading to a ferromagnetic metallic ground state at low temperatures for manganites with a large bandwidth.^10^ The CMR effect is strongly pronounced near the Curie temperature, where the ferromagnetic spin ordering starts to become disordered. At this temperature, an external magnetic field can substantially affect the reordering of the spins, elevate the Curie temperature, and, thus, reduce the electrical resistance by the double exchange. The CMR in mixed-valence manganese oxides (R_1–*x*_D_*x*_)­MnO_3_ (R = trivalent cation, e.g. rare-earth, D divalent cation, e.g. alkaline earth), like Pr_1–*x*_Ca_*x*_MnO_3,_ is particularly pronounced in the *x* = (0.3–0.4) doping region (Figure 2a).^16^ This is because the mechanism of charge ordering, which is most pronounced for a doping of 0.5, is destabilized.^17^ Hence, PCMO with the composition Pr_2/3_Ca_1/3_MnO_3_ has been a primary focus in this context.

**Figure 2:**
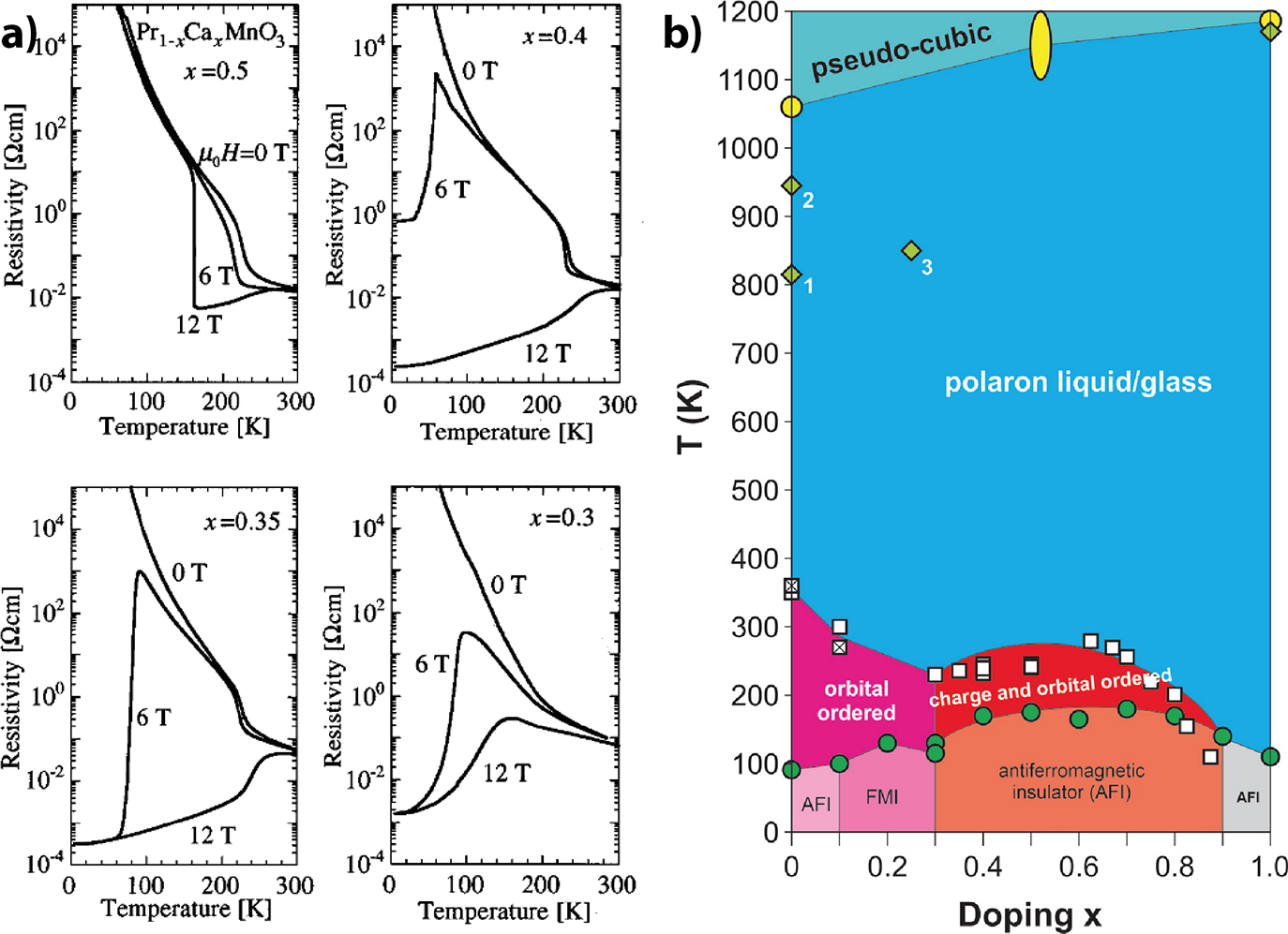
a) CMR effect in PCMO for Ca doping from 0.3 to 0.5. The resistance change is seen in the difference between the magnetic field applied, and the no-field applied cooling curves. Taken from ref 17. Copyright 1996 by American Physical Society. b) Phase diagram of PCMO taken from ref 18. Copyright 2021 by American Physical Society.

PCMO is characterized by low bandwidth,^10^ which leads to an insulating behavior over the entire Ca doping range (Figure 2b). It shows no ferromagnetic metallic ground state at low temperatures. Instead, it shows a charge-ordered state in a broad doping range around *x* = 0.5. Additional at lower temperatures an antiferromagnetic ordering forms.^17–20^

Applying a magnetic field, below the Neel temperature, can melt this charge-ordered state and introduce a ferromagnetic metallic state. This state is not considered to be a homogeneous phase, it is an electronic-magnetic percolation phase within the single-crystal.^10^ The resistance difference to the ground state becomes enormous for a lower conducting small bandwidth Manganite such as PCMO.

The charge-ordered state can also be melted by high currents or high fields, which lead to resistive switching in PCMO with noble ohmic contacts like Pt/PCMO/Pt at low temperatures.^21–24^ This effect is the called colossal electro-resistance effect (CER) and relates to a current-induced polaron solid–liquid transition.^23^

### 2.2 Electronic Structure and the Influence on Conductivity

The electronic structure of PCMO is strongly influenced by the electronic state of the Mn ion and its hybridization with the oxygen ion. The electronic state of Pr^3+^ ([Xe] 4f^2^) is usually seen as less important for the electronic properties, with contributions to the valence band and the higher energetic states of the conduction band (Figure 3a). Ca^2+^ is formally in the [Ar] noble gas configuration and is generally considered unimportant in terms of contribution to the valence band and conduction band.^25^

**Figure 3:**
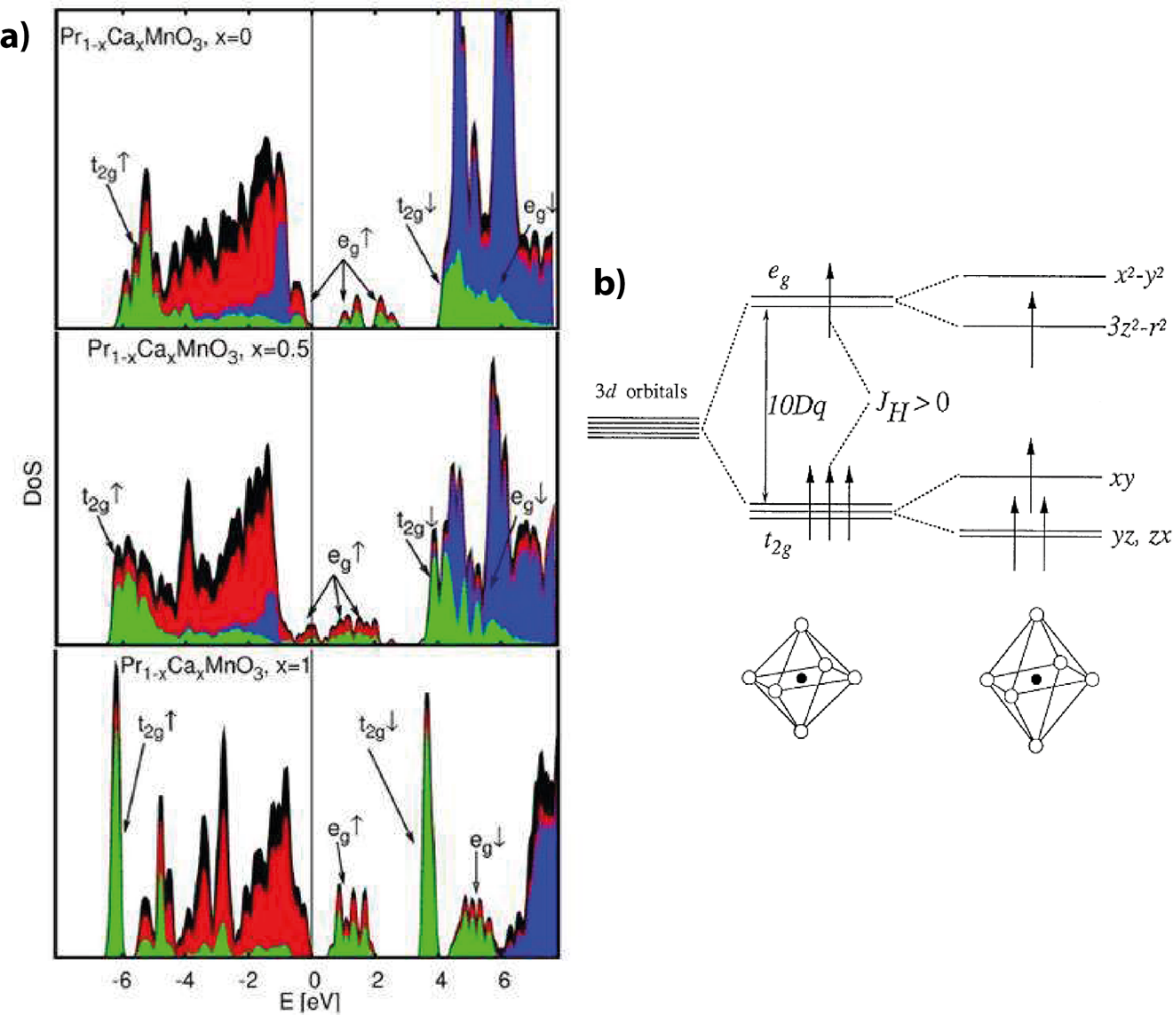
a) The black enveloping area shows the density of states of PCMO with calcium doping of 0, 0.5 and 1, calculated by DFT. The color represents the contributions from the atomic orbitals (red O 2p states, blues Pr d and f states, and green Mn d-states). Adapted and reprinted with permission from ref 25. Copyright 2012 by John Wiley and Sons. b) Crystal field splitting for Mn^3+^ high spin configuration in an octahedral crystal field – caused by the O^2–^ ligands. Adapted and reprinted with permission from ref 10. Copyright 2001 by Elsevier.

The electronic configuration of the Mn^3+^ and the Mn^4+^ ions is [Ar]­3d^4^ and [Ar]­3d^3^, respectively, without occupation of the s-shell. Since an octahedral crystal field surrounds the Mn ion in the perovskite structure, the d states become split into the six t_2g_ and four e_g_ electronic states (Figure 3). Hund’s rule and Coulomb repulsion results in further spin-dependent splitting of the t_2g_ and e_g_, states into spin-up and spin-down bands (Figure 3b). Therefore, the spin-split bands e_g_ and t_2g_ contain two and three remaining states, respectively.^26^

For CaMnO_3_, the 3d^3^ electron configuration of Mn^4+^ completely occupies the t_2g_ band, resulting in an insulator. The spin-dependent energy splitting of t_2g_↑ and t_2g_↓ is strong because of a high Coulomb repulsion described by U in the Hubbard Model. Thus, the t_2g_↑ band lies lower than the oxygen p-band (Figure 3a), and CMO has to be regarded as a charge transfer insulator.^27–29^

For pure Mn^3+^ as in the case of PrMnO_3_, the two degenerated e_g_ states are filled with a single electron. According to the Jahn–Teller (JT) theorem, this degeneracy is lifted by a distortion of the ligand octahedron. There are two different ways of distortion. Compression in the *xy* plane and an extension along the *z* direction. This splits the e_g_ band by energetically lowering the *d*_z^2^_ state and increasing the *d*_*x* ^2^ ‑ *y* ^2^_. Also, the t_2g_ band is split by lowering the *d*_*yz*_ state and increasing the *d*_*xy*_ and *d*_*xz*_ states. The distortion is the extension in the *x-y* plane and the compression along the *z* axis. This increases *d*_z^2^_, *d*_*yz*__,_ and *d*_*xz*_ in energy and lowers *d*_*x* ^2^ ‑ *y* ^2^_ and *d*_*xz*_ energetically.

If Mn^3+^, the JT-splitting of the e_g_↑ band leads to a one electron band. For PrMnO_3_ (PMO), this band is fully occupied, causing it to be an insulator (Figure 3a). Inge et al.^28^ and Rosenberg et al.^30^ have called PMO a Mott insulator. However, this label does not necessarily reflect the nature of the insulating state, since the e_g_↑ splitting is caused by the JT effect and the Coulomb interaction of the two e_g_^1^ and e_g_^2^ states is reduced due to their spin parallelism.

The substitution of Pr^3+^ by Ca^2+^ leads to a hole doping of the one electron e_g_ band of PrMnO_3_ and causes formally a mixed valency of the Manganese in the ratio of Mn^3+^_1–*x*_/Mn^4+^_*x*_. From a perspective of simple band theory, Pr_1–*x*_Ca_*x*_MnO_3_ should, therefore, be a conducting material since the band has unoccupied states and the charge carrier concentration is high with 10^21^–10^22^ /cm^3^ already for small Ca concentrations of a few atomic percentages.

Surprisingly, PCMO shows quite insulating behavior over its entire doping range. This can be explained by the low charge carrier mobility. A simple argument can be drawn from the connection of bandwidth and Fermi velocity from the simple tight-binding model. The narrow bandwidth, caused by the octahedral tilting, reduces the charge carrier’s Fermi velocity and, therefore, increases the lattice’s coupling. Further, each electron in the one-electron e_g_ band relates to a lattice distortion based on the Jahn–Teller effect. This couples the movement of an electron with the movement of a lattice distortion. Both factors lead to a polaronic description of the charge transport in PCMO, which results in a substantial increase in the effective mass and a strong reduction in charge carrier mobility.^22^

The strong connection of the Jahn–Teller mode with the bandwidth has been shown by a metal–insulator phase transition from the CO phase caused by controlled stimulation of the Jahn–Teller mode in a pump–probe experiment.^31^

### 2.3 Detection of the Charge Carrier Type

The Hall and Seebeck measurements indicate that the carrier type in PCMO changes with the Ca doping.^9,32^ Besides from direct measurements, the charge carrier type in PCMO can also be determined indirectly by the interaction of PCMO in a junction.^33^

From the perspective of a narrow e_g_ band, the charge carrier type can be described in dependence on the band filling *c*, which means the ratio of filled states *n* to the total amount of states *n*_c_ in the e_g_ band *c* = *n*/*n*_*c*_. In the context of mixed valence, this can also be described by

2.2 $$c\approx \left[{\mathrm{Mn}}^{3+}\right]/\left(\left[{\mathrm{Mn}}^{3+}\right]+\left[{\mathrm{Mn}}^{4+}\right]\right)$$

or in the case of perfect oxygen stoichiometry

2.3 $$c\approx \left[{\mathrm{Pr}}^{3+}\right]/\left(\left[{\mathrm{Pr}}^{3+}\right]+\left[{\mathrm{Ca}}^{2+}\right]\right)$$

If *c* < 0.5, then the e_g_ band is less than 50% filled with electrons. Therefore, it is an n-conductor. If *c* > 0.5, the filling is more than 50%, and thus, p-type. Interestingly, measurement data of the Seebeck coefficient indicate that the carrier type change happens between Ca_0.34_ and Ca_0.37_ and not as expected at Ca_0.5_.

The experimental determination by the Hall effect, caused by a magnetic field *B*_*z*_, is difficult for PCMO. Since the Hall voltage^26^

2.4 $$V_{y}=\frac{B_{z}j_{x}}{ne}$$

decreases with high carrier density *n* and low current *j*_*x*_ it is difficult to measure the Hall effect on a material with high carrier density and low mobility. Nevertheless, Asanuma et al.^32^ measured the Hall resistance *R*_*H*_ of CaMnO_3_ and Pr_0.14_Ca_0.86_MnO_3,_ which showed n-type conductivity. Further, they tried to measure the Hall resistance *R*_*H*_ at a Ca content of between 0.5 and 0.8 but were unable to obtain reliable measurements because of the high charge carrier density.

The highly rectifying character of a Pr_0.7_Ca_0.3_MnO_3_/Nb:STO junction (highly rectifying because of the low n-type doping SrTi_0.9998_Nb_0.0002_O_3_) showed that the description with a p-n type model fits better as the description with a Schottky junction model since the built-in voltage determined from the 1/*C* ^2^ – *V* characteristics is the same as the built-in voltage determined from the *J*/*T* – 1/*T* characteristics. This can be regarded as verification for the description of Pr_0.7_Ca_0.3_MnO_3_ as a p-type semiconductor.

The determination of the charge carrier type by the Seebeck effect is performed by measuring the sign of the Seebeck coefficient or thermopower *S* which relates to the thermoelectric voltage Δ*V* in a material with the temperature difference Δ*T* in a material by

2.5 ΔV=SΔT

The voltage is caused by the diffusion of the mobile carrier from the hot source to the cold sink. Therefore, the cold part of the material is charged according to the majority type of mobile charge carriers. *S* is negative (*S* < 0) for n-type materials and is positive (*S* > 0) for p-type materials. This consideration is true for doped semiconductors with clearly defined charge carrier characteristics but becomes more complicated for transition metal oxides.

The Heikes formula can be used,^26,34^ to describe the Seebeck coefficient in materials with narrow band and localized charge carriers with hopping mobility. It describes with

2.6 $$S=\frac{k_{B}}{e}\;\log\left(\frac{c}{1-c}\right)$$

the dependence of the Seebeck coefficient from the filling state *c* of the narrow band. The term log­(*c*/(1 – *c*)) describes the previously described sign change at *c* = 0.5. The term monotonously increases for both filling directions, *c* → 0 and *c* → 1, and diverges at 0/1 to −/+ ∞.

Interestingly, the Heikes formula predicts that the Seebeck coefficient only depends on the band’s filling and not on the temperature. Therefore, the Heikes formula should only be applied in cases where the Seebeck coefficient is temperature independent.

Seebeck coefficient measurements from PCMO for different stoichiometries and temperatures are shown in Figure 4. PCMO shows no strong temperature dependence at room temperature over its whole stoichiometry range except for the stoichiometry Pr_0.9_Ca_0.1_MnO_3_, as can be seen in Figure 4a.

**Figure 4:**
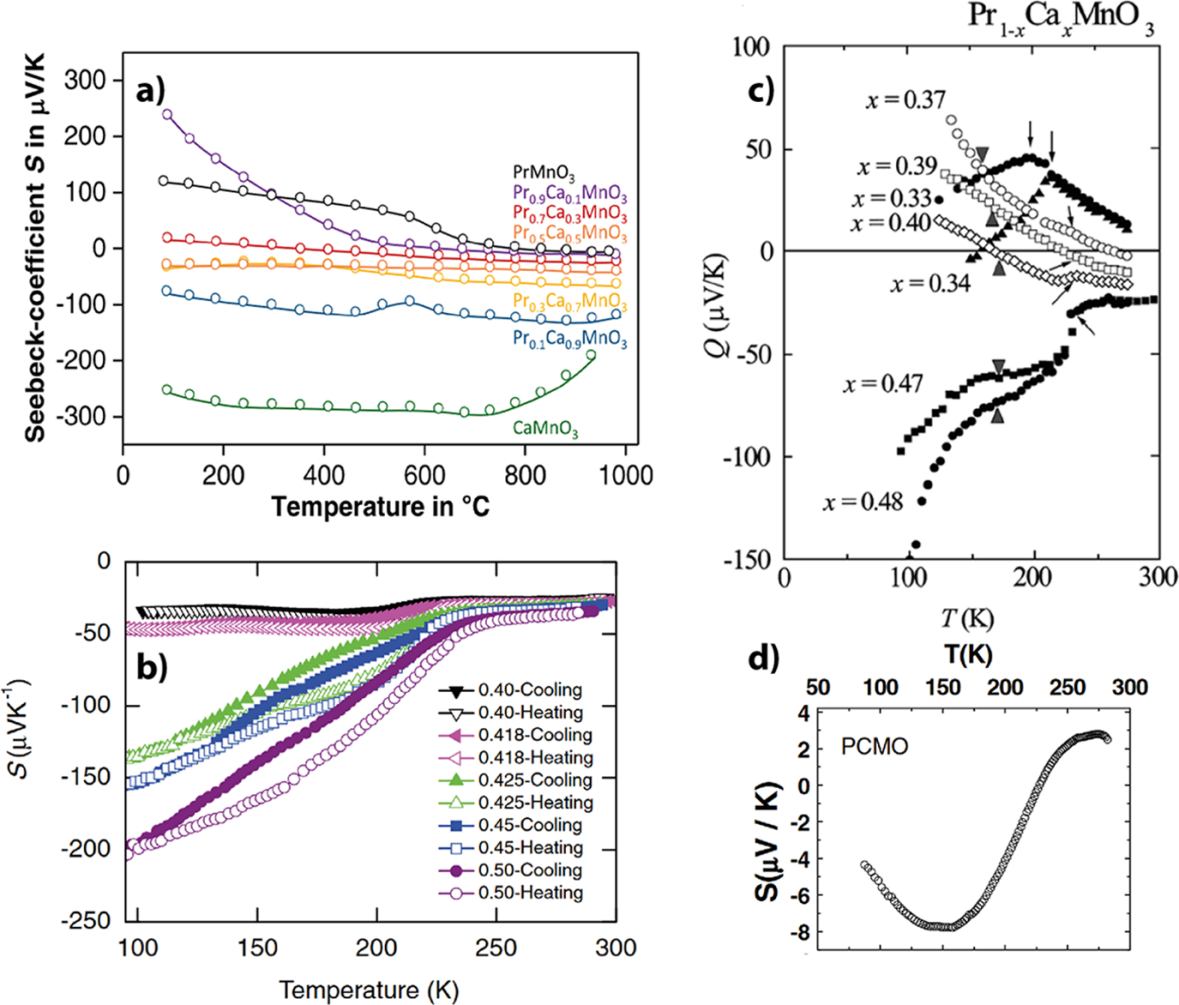
a) Seebeck coefficient from PCMO measured for different stoichiometries measured on bulk, polycrystalline PCMO. Adapted and reprinted with permission from ref 9. Copyright 2022 by Elsevier. b) Seebeck coefficient S from PCMO in the doping region Ca_0.4_ until Ca_0,5_, taken from ref 35. c) Seebeck coefficient *Q* of PCMO in the doping range between Ca_0.48_ and Ca_0.33._ Adapted and reprinted with permission from ref 36. Copyright 2000 by the Physical Society of Japan. d) Temperature dependence of the Seebeck coefficient of Pr_0.67_Ca_0.33_MnO_3_. Adapted and reprinted with permission from ref 37. Copyright 2007 by IOP Publishing Ltd.

Therefore, based on the interpretation of the sign of the Seebeck coefficient, Figure 4a shows n-type conductivity until a stoichiometry of Pr_0.5_Ca_0.5_MnO_3_ and p conductivity at the stoichiometry of Pr_0.7_Ca_0.3_MO_3_. At stoichiometry between Ca_0.5_ and Ca_0.3_, the type of conductivity changes cannot be extracted from Figure 4a. However, since the absolute value of the Seebeck coefficient is smaller for Ca_0.3_ than for Ca_0.5_, the expected stoichiometry, which leads to the change of the carrier type, is probably closer to Ca_0.3_.

A more precious measurement on the stoichiometry range between 0.5 and 0.4 can be found in Figure 4b. It shows the temperature independent negative Seebeck coefficient in the range from 250 to 300 K, continuously decreasing from Ca_0.5_ to Ca_0.4_. This data verifies the measurement from Figure 4a and further narrows the stoichiometric interval of sign change to the range between Ca_0.3_ and Ca_0.4_. This has also been shown by Kozakov et al.^14^

Figure 4c shows the Seebeck coefficient in the stoichiometric region Ca_0.33_ to Ca_0.48_. Most of the data stems from a temperature region with strong *S­(T)* dependence. The influence of *T* on *S* becomes particularly strong for temperatures below ∼230 K, when the CO phase begins to form. The data at the highest temperature of ∼270 K within the plateau region can be regarded as an approximation for room temperature for the change from p-type to n-type conduction in the stoichiometric region between Ca_0.37_ and Ca_0.33_.

The positive diminishing Seebeck coefficient at Ca_0.33_ was also reported by Venkataiah et al.^37^ and is depicted in Figure 4d.

The behavior of PCMO does not fulfill the Heikes formula in the approximate temperature independent region at room temperature, since it would predict a change in sign at Ca_0.5_. This deviation can be explained by considering the defect equilibria. Since metal vacancies dominate in the doping region of *x* < 0.5, as discussed in section 3.5, and each metal vacancy causes 2 holes, the real hole concentration is higher than the formal doping by the Ca content:^9^

2.7 $$3O_{2}⇌4V_{M}^{’’’}+12{h_{\mathrm{VM}}}^{.}+6{O_{O}^{x}}^{.}$$

Defining the excess hole concentration caused by metal vacancies with *h*_VM_, *c* could be corrected in the following way

2.8 $$c=\frac{n-h_{VM}}{n_{c}}$$

Unfortunately, *h*_VM_ ≈ 0.15 is needed for a correction, but according to Figure 7 only *h*_*VM*_ ≈ 0.02 could be expected.

An additional correction could result from the assumption that a relevant subset *b* of the e_g_ electrons are tightly bound and, therefore, do not play a role in the calculation of the configuration entropy. This would change *c* to

2.9 $$c=\frac{n-h_{VM}-b}{n_{c}-b}$$

The Seebeck data shows that the carrier type in Pr_0.7_Ca_0.3_MnO_3_, the most common stoichiometry for resistive switching devices, cannot simply be described as a p-type, as is often done in the literature. Further, the picture of a charge carrier type change at a certain doping is not consistent with the fact that the conduction mechanism in PCMO is based on a small polaron model, as will be described in the next section. In this model, the description of the charge carriers does not change at a certain stoichiometry.

### 2.4 Polaronic Model of PCMO and Characterization by Optical, Vibrational, and Electric Properties

The polaronic nature of PCMO is not only based on the idea, that the polarization of the matter causes the localization of a charge carrier, as reflected in the word polaron. The polarization localization is based on the difference in the dielectric constant at different frequencies. The permittivity is much higher at static frequencies (*ε*_static_) than at high frequencies (*ε*_high‑ω_), since the contributions of dipolar, molecular, and electronic polarization can be neglected. Therefore, for materials with high permittivity, a localized charge in a sphere of the radius *r* has a higher potential energy by polarization as a fast-moving (delocalized) charge, given in a simple electrostatic model^26^ by

2.10 $$\Delta E=\frac{-e^{2}}{8\pi \varepsilon_{0}r}\left(\frac{1}{\varepsilon_{static}}-\frac{1}{\varepsilon_{high‐\omega }}\right)$$

This localization mechanism therefore competes with the delocalization energy represented by the kinetic energy *D* of the electron, which is half of the bandwidth. Since PCMO is a narrow-bandwidth Manganite,^10^ mechanisms that provide potential energy gain from localization can easily compete with the energetic benefits of delocalization.

Polarization mechanisms can contribute, but the main mechanism of the polaronic trapping in PCMO is based on the Jahn–Teller effect which causes a splitting of the degenerated, e_g_ band and as a result a local lattice distortion. Therefore, the polaronic model is not based on a continuum model as in the polarization-based description by the Fröhlich polaron. Instead, the model to describe PCMO requires a local configuration coordinate to parametrize the distortion as in the Holstein model.^38^

A common scheme for the graphical representation of a configuration coordinate q-based description of the energy landscape is seen in Figure 5a.^26,39^ The black line shows the energetic configuration of an electron on either the left or right cation of a two-atom model. The activation energy required to jump between these two states is shown by *E*_act_, and a photonic absorption-activated change of the electronic configuration is shown by the absorption of energy *E*_opt_. According to the Franck-Cordon principle, this process is likely a photonic absorption process.

**Figure 5:**
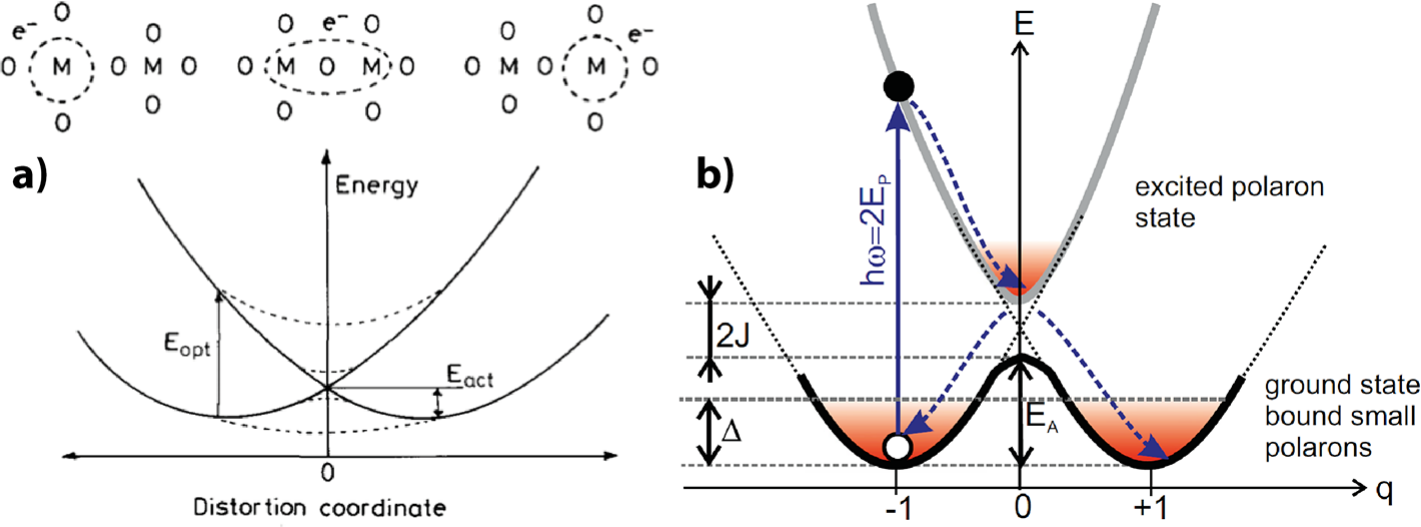
a) General picture of a configuration coordinate model. Adapted and reprinted with permission from ref 26. Copyright 2006 by John Wiley and Sons. b) Configuration coordinate model for PCMO. Adapted and reprinted with permission from ref 40. Copyright 2015 by the American Physical Society.

The polaronic nature of the charge carrier of PCMO has been described in the literature by a small polaron within the framework of the Holstein model.^40^

The Holstein model is described by a Hamiltonian,^41,42^ which consists of a sum of three terms. Each term has an energy scaling factor, which scales the individual contribution to the system’s total energy. The factors are the phonon energy *ℏ*ω, the coupling energy *g* between the electron and the phonon, and the transfer integral *J*. The Holstein Hamiltonian can be simplified by imposing conditions on the ratio of the energy scales, leading to the model of the small polaron.^42,43^ Small means that the localization of the polaron is of the size of the lattice spacing, which refers to the lattice on which the Holstein model is based and, thus, to the bonding distance. The configuration coordinate model for PCMO is illustrated in Figure 5b.

The transfer integral *J* gives the delocalization energy of the electron over the bond and represents multiplied by the amount of its nearest neighbors Z (PCMO *Z* = 6, octahedral) with *ZJ* = *D* the kinetic energy of the electron, which is half of the bandwidth.^40^ For Pr_0.5_Ca_0.5_MnO_3_ the bandwidth can, can be estimated by DFT calculations to be between 760 and 1300 meV, giving an estimate of *J* between 60 and 110 meV.^44^ Experimentally, values can be derived from measurements of the thermally activated hopping conduction, leading to *J* between 90 and 110 meV,^40^, depending on the stoichiometry.

The energy of the Jahn–Teller involved phonon mode can either be 42 or 71 meV.^31^ The lower energetic modes are connected with octahedral tilting modes, while the higher energetic modes are considered to be connected with Jahn–Teller stretching and breathing modes.^40,45^ Thus, the higher energetic Jahn–Teller modes influence the hopping by influencing the tolerance factor and, therefore, indirectly, the octahedral tilting, while the lower energetic modes are bending modes of the bonding angle. The stimulation of the 71 meV mode can cause a phase transition from insulator to conductor in the CO phase by perturbating the tolerance factor and thus reducing the distortion of the Mn–O–Mn angle, as has been experimentally seen in pump–probe measurements.^31^

Another experimental way to determine the energies of the involved phonon modes is by optical investigation of two different optical excitations, shown in Figure 5b. PCMO shows two absorption features in the near-infrared region, which relate to the Franck–Condon on-site Jahn–Teller-excitation and the polaron hopping,^40^ respectively. The width of these photon absorption peaks is caused by the variance in the ground state Δ and, therefore, relates to the energy of the involved phonon modes. By fitting the peak width, phonon energies can be determined. The on-band Jahn–Teller-excitation gives energies of the involved phonon modes of 100–120 meV, while the hopping transition leads to phonon modes between 36 and 60 meV.^40^ Interestingly, the phonon modes for hopping could be the tilting mode of the Mn–O–Mn angle.^40^ However, the mathematical calculation of the involved phonon energies from the peak width wrongly assumes *J* = 0, which could affect the precision of the determined energy value.^40^

The strongest localization of the polaron occurs under the condition that the kinetic energy of the electron is smaller than the phonon–electron coupling *J* ≪ g, and the kinetic energy of the electron is smaller than the energy of the involved phonon mode *J* ≪ ℏω.^42^ In the atomic limit J = 0, the simplified Hamiltonian can be transformed to a single oscillator picture, which defines the new particle, the small polaron. It connects each phonon mode with a lattice distortion and has a defining parameter α = *g*/*ℏ*ω,^42^ which gives the strength between the coupling of the lattice vibration and the electron. For high α, the electron is strongly coupled with the lattice vibration, and the polaron binding is high. The polaron bonding energy of this model is *E*_*p*_ = *g* ^2^/*ℏ*ω.^42^ As can be seen in Figure 5, the polaron binding energy is directly connected to the Franck–Condon optical absorption peak and can be investigated by optical conductivity measurements. From the approximation of a local parabolic energy landscape, as drawn in the configurational coordinate model of Figure 5b the equation *E*_*p*_ = *E*_photon_/2 can be geometrically derived. It relates the polaron binding energy directly to the absorption energy of the involved photon, which is only valid when the kinetic energy of the electron *D* is smaller than the energy variance in the ground state Δ.^40^ This is not the case for PCMO and therefore the photon energy of the connected absorption must be corrected by the term *E*_photon_/2 + *D* ^2^/(4*E*_*p*_) = *E*_*p*_.^40^ This allows the polaron binding energy to be determined from the frequency of the absorption peak if a correct value of the kinetic energy *D* is used.

In order to include hopping into the model, the transfer integral *J* cannot be zero. The parameter γ = *ℏω*/*J* can be defined for a mathematical description. For strong coupling α > 1, the main guiding force of the movement of the electron is the electron-coupled lattice distortion. If the delocalization energy *J* dominates (γ → 0), the lattice follows the electron, and an adiabatic description of the hopping process can be used.^40,42^ The opposite case is called antiadiabatic and would lead to a substantial increase in the effective polaron mass.^42^ Since for PCMO *J* and ℏω are of similar sizes, the adiabatic approximation is not strongly fulfilled, and PCMO is between adiabatic and antiadiabatic regime.^22,40^ This gives a constraint to the equation for polaron hopping by an electric field, which was derived under adiabatic assumptions^22^ and gives

2.11 $$\frac{\sigma \left(E,T\right)}{\sigma_{0}\left(T\right)}=\frac{E_{C}\left(T\right)}{E}\;\sinh\left(\frac{E}{E_{C}\left(T\right)}\right)$$

where σ_0_ is the ohmic conductivity dominating at small fields and *E*_*C*_(*T*) the crossover field between ohmic and nonohmic behavior

2.12 $$E_{C}\left(T\right)=\frac{2k_{B}T}{ea}$$

with the polaron hopping length α.^22^

For a finite *J*, the ratio λ = *E*_p_/*D* of the previously defined polaron formation energy *E*_p_ and the kinetic energy *D* of the electron shows how stable the polaron is against delocalization. In the adiabatic limit λ and α are the two defining parameters for the polaronic model.^42^ Strong coupling (λ > 1) is a requirement for the model of a small polaron.^40^

In the case of strong coupling, each jump of the electron relates to a localized jump of the lattice distortion and thus, to a Jahn–Teller-based rearrangement of the electronic structure. Therefore, the concept of the configuration coordination model can be specified for the polaron hopping in PCMO as shown in Figure 5b.^40^ The broadening of the symmetric state of delocalization, given by 2*J*, lowers the activation energy for hopping.^39^ In the simple configuration model with the parabolic assumption^39^ the necessary *E*_*A*_ activation energy for the polaron hopping in PCMO becomes

2.13 $$E_{A}=E_{P}/2-J$$

This equation has to be corrected for a strongly correlated Manganite by an intersite Coulomb repulsion term *E*_*C*_ to

2.14 $$E_{A}=E_{P}/2-J+E_{C}$$

At small fields, the conductivity of the above-defined term is dominated by the conductivity σ_0_(*T*), which

2.15 $$\sigma_{0}\left(T\right)=\frac{A}{T}e^{-E_{a}/k_{b}T}$$

which is thermally activated by the energy *E*_*a*_ of the small polaron hopping. The factor *A* consists of^22^

2.16 $$A=\frac{ne^{2}a^{2}\omega_{0}}{2\pi k_{B}}$$

the charge density *n*, the elementary charge *e*, the hopping length *a* and the frequency of the involved phonon mode ω_0_. The conductivity increases with the temperature, as can be seen in Figure 6a and b and can lead to the wrong assignment as semiconducting property. However, unlike in a semiconductor, the charge carrier density does not increase with temperature; instead, the mobility does.

**Figure 6:**
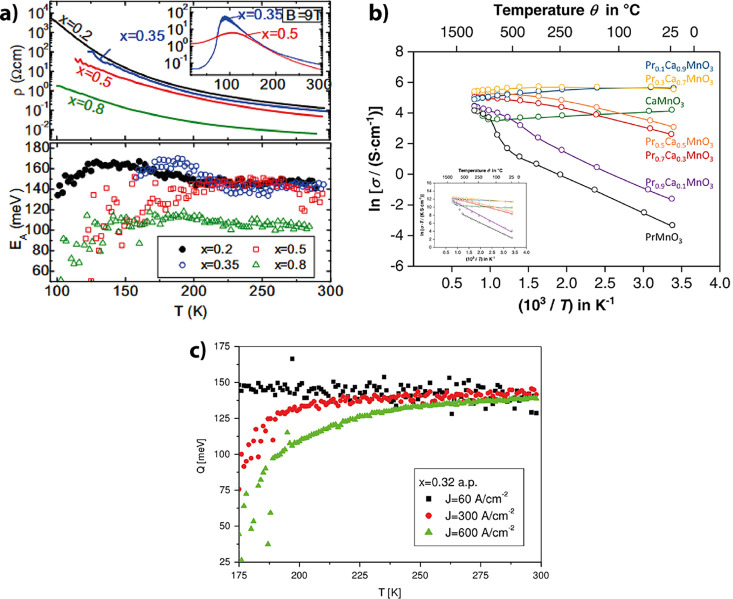
a) Temperature dependence of the resistance and the fitted activation energy of PCMO with different doping concentrations *x*. The inset graph verifies the CMR effect. Adapted and reprinted with permission from ref 40. Copyright 2015 by the American Physical Society. b) Temperature dependency of conductivity for bulk polycrystalline Pr_1–*x*_Ca_*x*_MnO_3_ with different stoichiometries. For *x* < 0.7, temperature-activated polaron hopping describes the dependency. The linear fit in the inset gives the following activation energies: *E*_a_(Pr_0.3_) = 43 meV, *E*_a_(Pr_0.7_) = 128 meV, and *E*_a_(Pr_0.9_) = 259 meV. Adapted and reprinted with permission from ref 9. Copyright 2022 by Elsevier. c) Activation energy of the small polaron of Pr_0.68_Ca_0.32_MnO_3_ for three different current densities in dependence of the temperature. Adapted and reprinted with permission from ref 22. Copyright 2008 by IOP Publishing Ltd.

It is evident from Figure 6a and b that the overall conductivity decreases with the decrease of Calcium doping for *x* < 0.7. This is probably caused by the increase of Mn^3+^ with larger ionic radius, causing a decrease in tolerance factor, bending angle, and hopping conductivity.

Fitting the temperature dependence in Figure 6a shows that the activation energy is quite independent of the stoichiometry from Ca_0.2_ to Ca_0.5_ for temperatures higher than the CO phase, and that Pr_0.65_Ca_0.35_MnO_3_ has an activation energy of 145 meV at room temperature. These values are varying in the literature, e.g. Figure 6b measured an activation energy of 128 meV for Pr_0.7_Ca_0.3_MnO_3_. This can probably be explained by a difference in current densities, as Schramm et al. showed for Pr_0.68_Ca_0.32_MnO_3_ (Figure 6c) that the variation in the activation energy increases with lower current densities.

### 2.5 PCMO Defect Chemistry and the Influence of Oxygen Vacancies

Point defects are part of the thermal equilibrium since they lower the thermodynamic potential by the increase of the configurational entropy. This can happen due to intrinsic defects, which are rearrangements of the existing material without changing stoichiometry or introducing impurities. Intrinsic defects are Schottky-defects (volume expanding vacancy generation), Anti-Schottky defects (volume-reducing pair of interstitials), Frenkel-defects (cation interstitial by cation vacancies generation), and Anti-Frenkel (anion interstitial by anion vacancy generation). For dense perovskites, the formation energy of interstitial is very high, and therefore, Schottky defects are particularly relevant,^4^ which form according to the following equation

2.17 $$2M_{M}^{X}+3O_{O}^{X}⇌M_{2}O_{3}+3\overset{¨}{V_{O}}+2V_{M}^{’’’}$$

Point defects can also be caused extrinsically by exchange reactions with the surroundings. This causes nonstoichiometry and electronic doping. Oxygen exchange reaction can also take place by field activation at cathode/anode interfaces which is the basis of resistive switching in the case of the valence change mechanism. For oxides in contact with the atmosphere, the exchange is especially the incorporation or release of oxygen as formalized below. PCMO is generalized by M_2_O_3_, where M stands for the metal cations.

The incorporation of oxygen leads to metal vacancy *V*_*M*_^’’’^ formation and hole doping

2.18 $$3O_{2}⇌4V_{M}^{’’’}+12h^{.}+6O_{O}^{x.}$$

The release of oxygen leads to the formation of oxygen vacancies *V*_*Ö*_ and electron *e*’ doping

2.19 $$O_{O}^{x}⇌\overset{¨}{V_{O}}+2e^{\prime }+\frac{1}{2}O_{2}$$

The oxygen stoichiometry, therefore, shows the type of vacancies generated by exchange with the atmosphere and the overall modification of the valence. For bulk PCMO^9^ (sintered at 1350 °C and cooled slowly below 1 K/min in oxygen atmosphere) the dominating point defects are oxygen vacancies *V̈*_O_ at a doping of *x* > 0.5 and metal vacancies *V*_*M*_^’’’^ at a doping of *x* < 0.5 as can be seen from the measurement of the oxygen nonstoichiometry shown in Figure 7a. The relative amount of both types of vacancies is increasing for *x* → 1/0.

**Figure 7:**
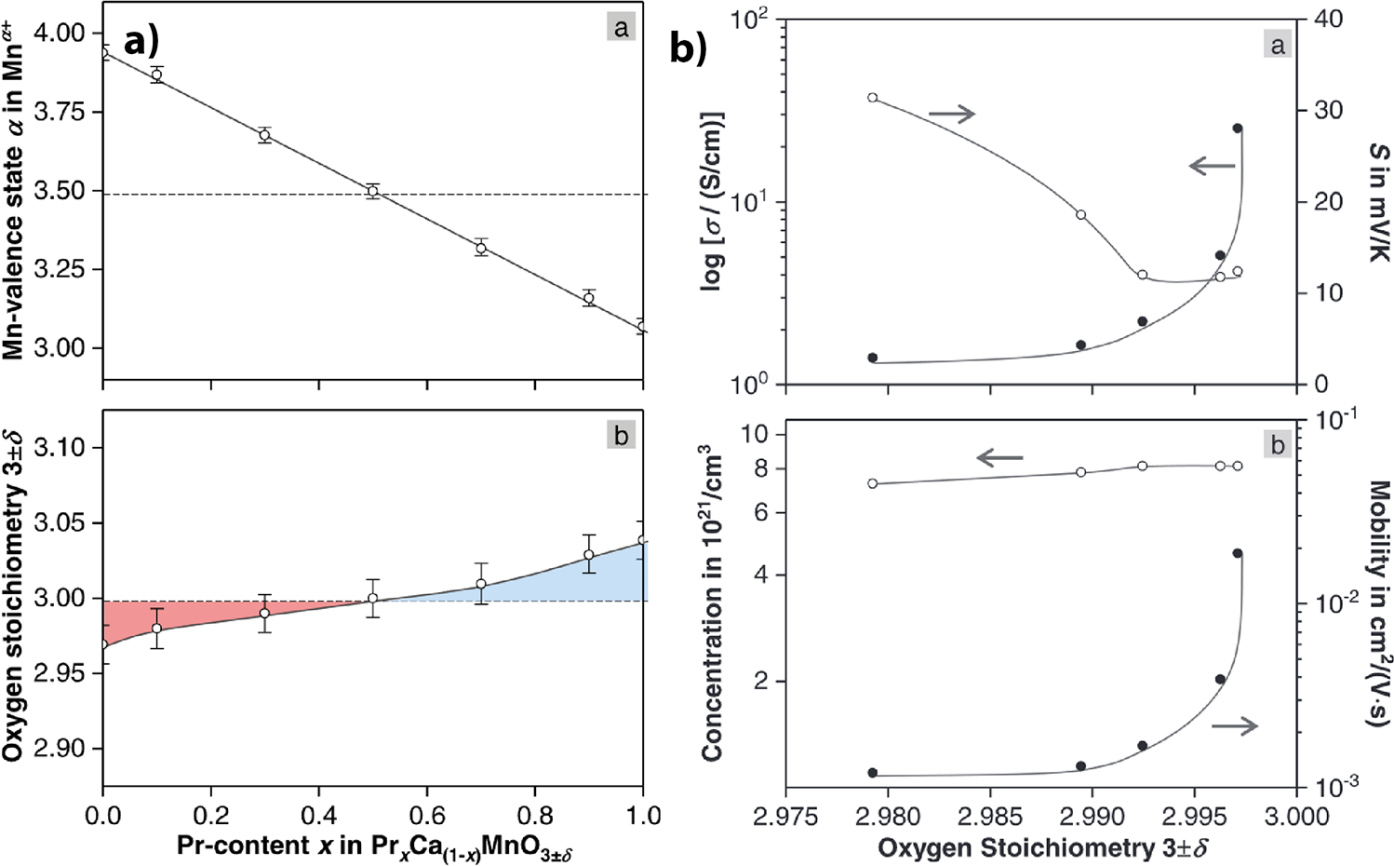
a) Oxygen nonstoichiometry of PCMO, doping dependent. Measured on bulk polycrystalline samples by iodometric titration. Adapted and reprinted with permission from ref 9. Copyright 2022 by Elsevier. b) Change of Seebeck coefficient, charge carrier density, and mobility by oxygen vacancy formation in Pr_0.7_Ca_0.3_MnO_3_. Adapted and reprinted with permission from ref 9. Copyright 2022 by Elsevier.

Oxygen vacancies *V̈*_O_ and metal vacancies *V*_M_^’’’^ can compensate each other by

2.20 $$0⇌3\overset{¨}{V_{O}}+2V_{M}^{’’’}$$

The hole doping by Ca^2+^ can be formalized by the doping with a generalized acceptor AO into the generalized metal oxide M_2_O_3_ ^46^

2.21 $$\mathrm{AO}+\frac{1}{2}O_{2}⇌2A_{M}^{’}+2h^{.}+3O_{O}^{x}$$

Since the metal vacancy contribution dominates in Pr_0.7_Ca_0.3_MnO_3_ in the thermal equilibrium at room temperature, the actual hole doping level can be considered higher than the formal hole doping level given by the calcium content.

For resistive switching, the effect of oxygen vacancies on the resistance is essential. The conductivity

2.22 σ=enμ

is the product of the charge carrier density *n*, and the mobility μ. The mobility is determined by the polaronic nature (eq 2.11, 2.15, and 2.16). Oxygen vacancies are considered to increase the resistance due to a reduction in mobility μ. In case of hole conducting PCMO, oxygen vacancies are also considered to increase the resistance due to a reduction of the charge carrier density *n*. As can be seen in eq 2.22, the reduction of the conduction is linear with a change in the charge carrier density and can be nonlinear if the effect of the vacancies on the mobility is nonlinear.

Figure 7b shows the impact of thermal reduction of Pr_0.7_Ca_0.3_MnO_3_ on conductivity and the Seebeck coefficient. An increase in the Seebeck coefficient experimentally verifies the reduction of the hole charge carriers with decreasing oxygen content. Moreover, a nonlinear decrease of the conductivity is measured. The strong nonlinear change of the conductivity in PCMO cannot be explained by the small linear change of charge carrier density as expected from the iodometric measurements. Pithan et al.^9^ further strengthen this argument by decomposing the conductivity into charge carrier density and mobility (Figure 7b) by calculating the carrier density from the Seebeck coefficient with the Heikes formula, which has some inaccuracy, as discussed in section 2.3. It can be concluded that the change in mobility is the dominating effect of the conductivity change, observed during reduction. However, this raises the question of what causes the mobility change in PCMO.

The activation energy of the polaron hopping depends strongly on the doping of the PCMO, as can be seen in Figure 6a,b. Over a broad doping range from *x* = 0.2 to *x* = 1, electron doping increases the activation energy and, therefore, the resistance.

Pithan et al.^9^ or Nian et al.^47^ assign the mobility reduction to the interruption of Mn–O–Mn chains, which are essential for polaron hopping (section 2.4). However, this cannot be the only reason since a small change in vacancy concentration, like in Figure 7, does not broadly disturb the percolation network of Mn–O–Mn chains or severely increase the effective hopping path length but already causes a mobility reduction.

Besides the influence of the doping on the activation energy, also structural arrangements of the vacancies *V*_*Ö*_ can play a role. Pithan et al.^9^ analyzed neutron diffraction pattern and concluded that the vacancies preferably form in the manganese planes of the perovskite.^9^ This causes a stripe or superstructure formation in the TEM, which can be manipulated and moved by the application of an electric field, as shown by Liao et al.^48^ (Figure 8).

**Figure 8:**
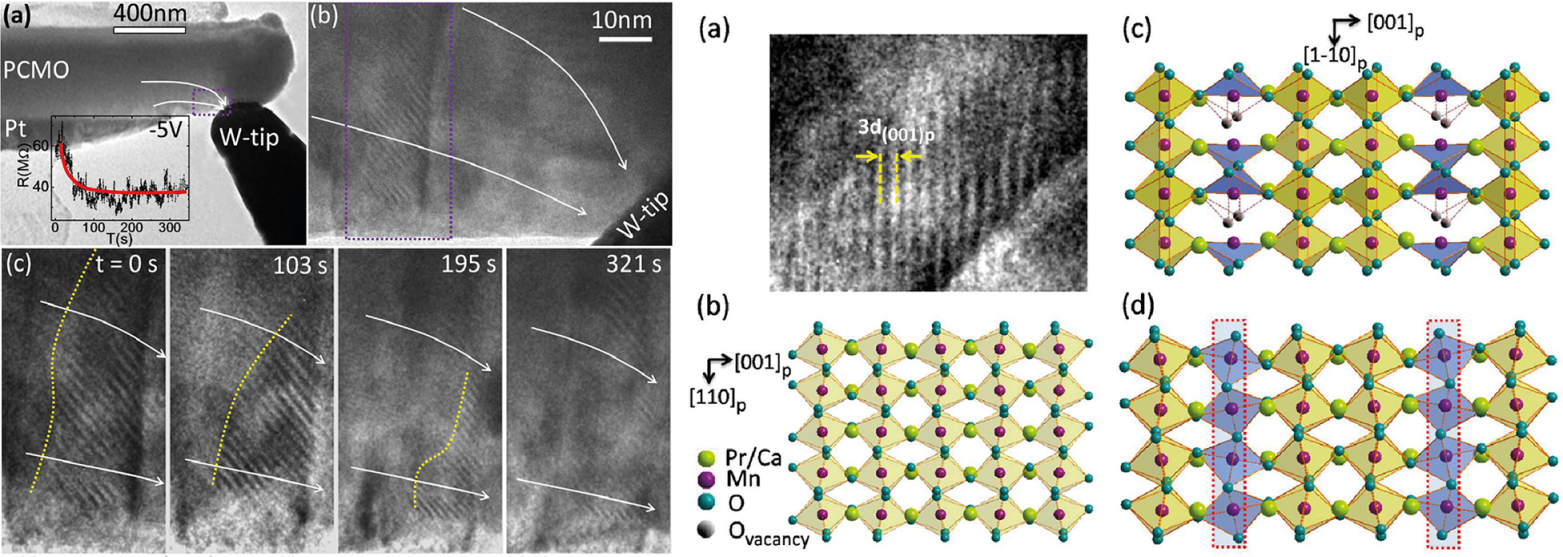
Left series of images shows the time-dependent movement of the oxygen vacancy stripe pattern by application of −5 V on the W tip, in situ, observed in TEM. The right series of images shows the proposed alignment of the oxygen vacancies in PCMO in the Mn-based planes, forming stripes. Adapted and reprinted with permission from ref 48. Copyright 2012 by AIP Publishing.

Jooß et al.^49^ also overserved this superstructure in the vicinity of the metal/oxide interface after a initializing process applied to a bulk PCMO by a Pt/Ir tip in a TEM, which is related to a resistance increase. They explained the structural change by a polaron order–disorder transition within the PCMO, which localizes the polarons. They further argued that this transition could be especially pronounced when a contact causes a down-bending of the bands in the PCMO because the hole depletion, similar to decrease of the Ca doping as can be seen in Figure 1, causes an increase in the octahedral tilting and therefore a stronger polaron localization.

Also, the increase of disorder in the Coulomb potential by high vacancy concentrations can lead to a mobility reduction as discussed in the framework of Anderson localization.^16^ This effect can especially be considered for narrow bandwidth manganites such as PCMO. Particularly since the mixed valence doping already causes disorder on the A site. Further, the orthorhombic tilting leads to an extended unit cell within which the Mn–O–Mn bonding angles and distances show small variations.^14^ This further increases the disorder in the electrostatic potential. The potential disorder can cause a separation of localized and delocalized charge carriers at the mobility edge, an energy level in the density of states.^16,26^ This idea is supported by DFT calculation can be seen in Figure 10b.^50^

To use this knowledge to gain deeper insights into the mechanism of resistive switching, it is important to know the concentration of oxygen vacancies in the interface region and its change during switching. If the field-driven vacancy concentrations in the interface regions are much higher than the *V̈*_*O*_ concentrations achieved by thermal annealing in a bulk sample, it will be important to clarify if their impact on the conductivity remains the same.

For a strong thermal reduction, Pithan et al.^9^ observed the appearance of additional phases PrMnO_3_ and Ca_2_Mn_2_O_4_ identified by additional Bragg reflections. The Ca_2_Mn_2_O_4_ phase start to appear at an oxygen substoichiometry of O_2.902_ and the PrMnO_3_ phase at an oxygen substoichiometry of O_2.786_. This shows that strong electrochemical reduction could even lead to phase separation.

Lee et al.^30^ conducted spectroscopic measurements on PCMO before and after electrochemical reduction induced in a scanning force microscope by a grid scan with a tungsten tip. They applied +4 V to extract the oxygen from the interface region at each contact point with the surface. This area of higher resistance is called HRS in Figure 9. The reference virgin surface with lower resistance is named LRS. Since this type of performed reduction reaction is similar to the oxygen extraction within a switching device, the observed spectroscopic changes can be assumed to be similar to the vacancy concentrations relevant for resistive switching.

**Figure 9:**
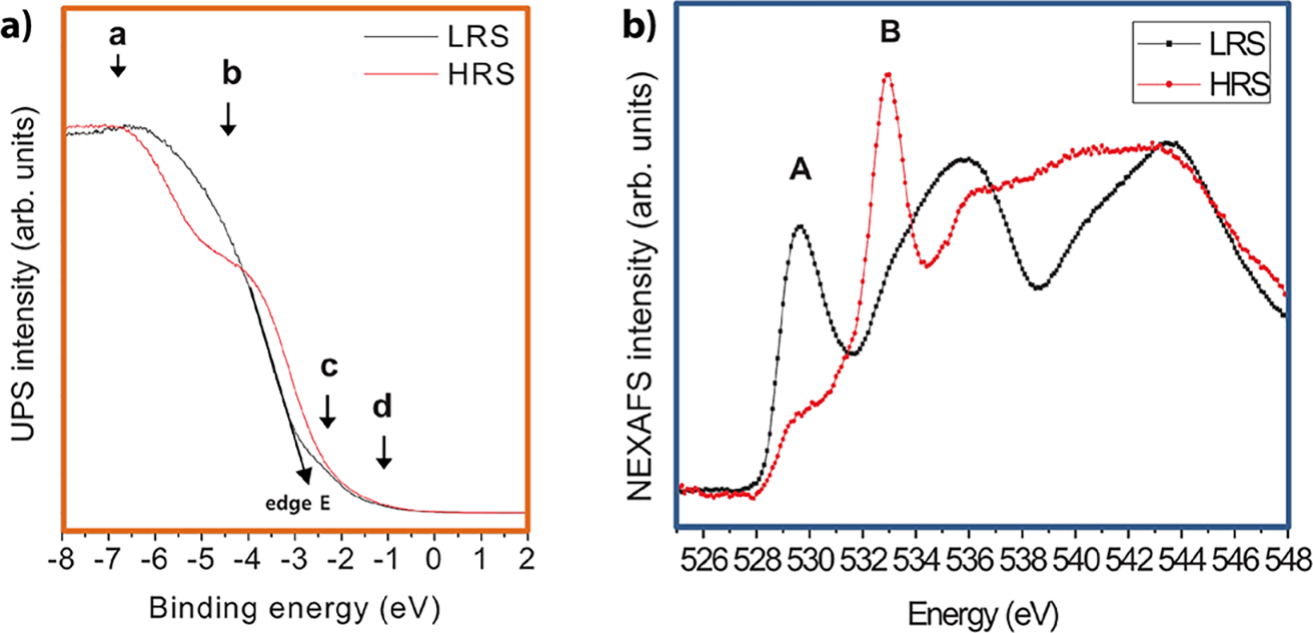
Influence of oxygen vacancies on Pr_0.7_Ca_0.3_MnO_3_ measured by UPS and XAS. LRS represents the as-deposited PCMO, and HRS represents the electronically reduced PCMO. a) shows the change in the UPS signal, and b) shows the change in the XAS signal. Adapted and reprinted with permission from ref 30. Copyright 2013 by Springer Nature.

The influence of oxygen vacancies on the charge carrier density can be seen spectroscopically in ultraviolet photoelectron spectroscopy (UPS) and X-ray absorption spectroscopy (XAS) measurements (Figure 9). The UPS shows for the reduced (HRS) PCMO a clear drop in the peak b corresponding to the reduction of O 2p binding states and an increase in the edge energy, corresponding to a filling of the e_g_ band.^30^ Further, the X-ray absorption near-edge structure (NEXAS) signal from the excitation from the O 1s state shows a decrease of the peak A with reduction. Since the peak A corresponds to the transition from O 1s into an empty e_g_ state, the decrease of peak A shows the filling of the holes by oxygen vacancy-induced n-type doping.

Lee et al.^30^ conclude from the spectroscopic measurements that the decrease of the resistance due to the reduction is caused by a metal–insulator transition based on the filling of the e_g_ band.

They further observed an increase in the band gap between the e_g_^1^ and e_g_^2^ bands. Experimental evidence is the decrease of peak A and the increase of peak B. The increase of peak B is interpreted as an energetic increase of the e_g_^2^ levels. An increase of the band gap also has been shown by UV/vis studies of Asanuma et al.^32^ Figure 10a shows that the band gap increases for PMO (0.4 eV), CMO (0.4 eV), and Pr_0.5_Ca_0.5_MnO_3_ (0.2 eV) after thermal reduction. Thus, a band gap increase by reduction happens for all filling levels of the e_g_^1^ band.

**Figure 10:**
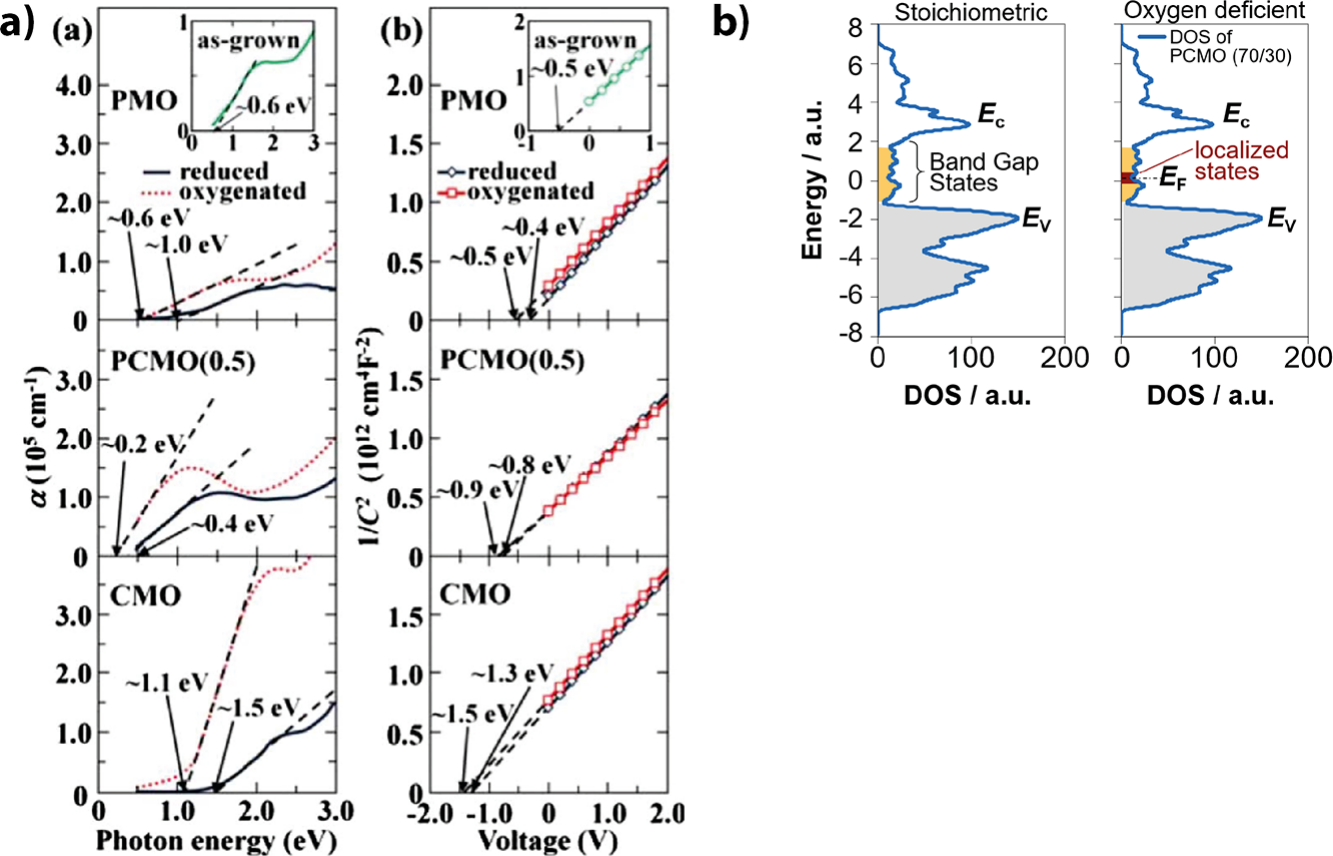
a.a) Change of the bandgap and a.b) 1/C^2^–V characteristic of PMO, CMO, and Pr_0.5_Ca_0.5_MnO_3_, oxygenated and reduced. Adapted and reprinted with permission from ref 32. Copyright 2012 by AIP Publishing. b) Proposed localization of electrons by oxygen vacancies in PCMO. Based on unpublished but presented DFT calculations from Rene Meyer.^50^

Lee et al.^30^ claim further that an increase in energetic separation between the e_g_^1^ and e_g_^2^ can be considered as an increase in Coulomb interaction interpreted within the framework of the Hubbard model. They conclude that the low resistance caused by the band filling relates to a Mott metal–insulator transition based on strong correlation effects. This argumentation can be seen critical, since the separation of the e_g_^1^ and e_g_^2^ levels is caused by the Jahn–Teller-splitting and the occupying electrons of the e_g_^1^ and e_g_^2^ levels have the same spins because of the strong Hund’s coupling. Therefore, their energetic separation cannot be connected to intersite Coulomb repulsion.

It can be summarized that oxygen vacancies increase the resistance and the band gap of the PCMO. Both can influence the heterojunction of a resistive device.

### 2.6 Work Function of PCMO

Since the current transport within PCMO-based memristive devices is influenced by the band bending at the interfaces, the work function of PCMO is an important parameter. The literature values of work functions can differ for different measurement methods and between differently deposited PCMO layers. A direct method of measurement is UPS or Kelvin-Probe measurements. Indirect measurements are based on the transport characteristics of heterojunctions like MOS (metal/oxide/semiconductor), pn-junctions, or Schottky diodes and the fitting of the transport characteristics by a model. The work function values, which can be found in the literature, are summarized in Table 1.

**Table 1:** Literature Values of PCMO Work Function

method	work function in eV	stoichiometry	source
Kelvin-Probe	4.896	Pr_0.7_Ca_0.3_MnO_3_	^51^
STO/PCMO pn-junction characteristics	4.4	PrMnO_3_	^32^
	4.6	Pr_0.7_Ca_0.3_MnO_3_	^33^
	4.8	Pr_0.5_Ca_0.5_MnO_3_	^32^
	5.4	CaMnO_3_	^32^
PCMO/ZrO_2_/SiO_2_/p-Si-MOS characteristics:	5.43	Pr_0.7_Ca_0.3_MnO_3_	^52^

As can be seen, the work function of PCMO depends on its doping. The MOS-characteristic measurement shows a higher value as expected from the other measurements, which is probably caused by a difference in growth conditions. The PCMO used for the MOS characteristics had been sputtered and postannealed for crystallization while in the other papers the films were grown by PLD directly at crystallizing temperatures. More details on the different work function measurements and the comparison of different valent manganites are discussed below.

Direct work function measurements by the Kelvin probe method for the different mixed valent manganates with the same doping Ln_0.7_D_0.3_MnO_3_ but with different Lanthanides La and divalent cations D with significant differences in the conductance show quite similar work functions (Figure 11a). All the combinations LaCa, LaSr, LaPb, PrCa, PrSr, and NdSr have work functions between 4.807 and 4.918 eV with Pr_0.7_Ca_0.3_MnO_3_ at 4.896 eV.^51^

**Figure 11:**
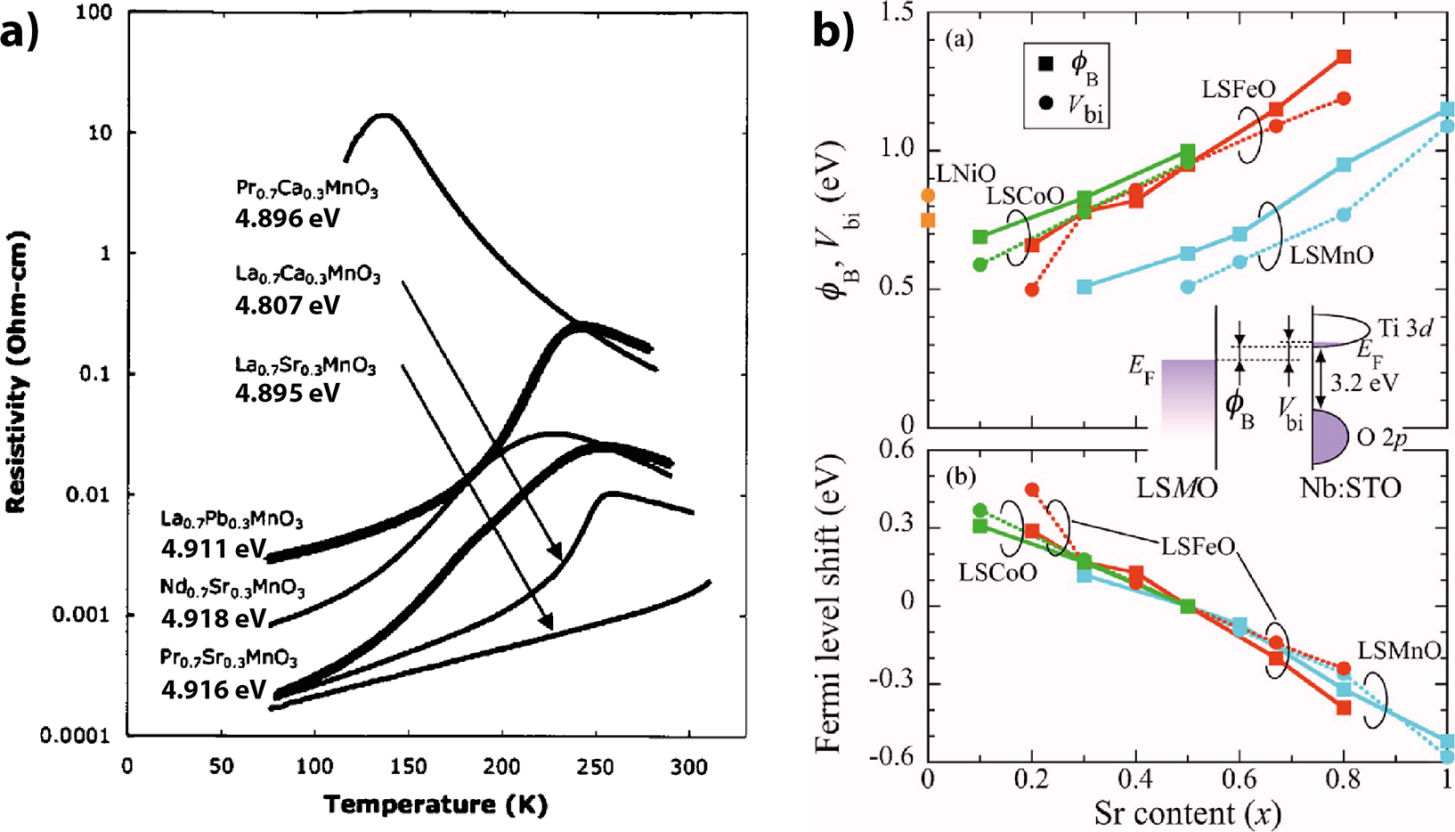
a) Temperature dependence of the resistance of different manganites with the same doping but different lanthanides and divalent ions. The resistance shows many orders of magnitude of variation. Kelvin probe measurements of the work function show low variation. Adapted and reprinted with permission from ref 51. Copyright 2004 by AIP Publishing. b) Shift of Fermi level in dependency of the doping in La_1–*x*_Sr_*x*_MO_3_, with M = (Mn, Fe, and Co), determined by the Schottky junction with Nb:STO, from the *I*–*V* (forward direction) characteristics and 1/*C* ^2^ – *V* (reverse direction) characteristic. Adapted and reprinted with permission from ref 53. Copyright 2007 by AIP Publishing.

Measurements of the interface between n-type doped SrTi_0.99_Nb_0.01_O_3_ and high charge carrier La_1–*x*_Sr_*x*_MO_3_ (where M is Mn, Fe, or Co) showed that it behaves like a Schottky junction.^53^ Determination of the Schottky barrier from the forward bias behavior, as well as determination of the built-in voltage from the *1/C* ^*2*^ *–V* characteristic in the reverse direction, shows that the work function of all three transition metal oxides increases linearly with the hole doping *x* (Figure 11b). The linear increase demonstrates that no Fermi-level pinning takes place at the interface.

Sawa et al.^33^ claims from the comparison between the junction of Pr_0.7_Ca_0.3_MnO_3_ and La_0.7_Sr_0.3_MnO_3_, respectively, with Nb:STO that the description of the highly rectifying junction with PCMO fits better to a description of a pn junction as to the description of a Schottky junction. An argument is that the PCMO junction does not show a temperature dependence of the leakage current up to an applied voltage of 100 V in the reverse direction compared to La_0.7_Sr_0.3_MnO_3_, where the leakage current increases with decreasing temperature. Further, they determined the built-in voltage from the temperature dependence of the *J*_0_ of the *J–V* characteristics in the forward direction. In a Schottky junction model, *J*_*0*_ has a linear temperature dependence in a *J/T – 1/T* plot, and in the model of a pn-junction, the linear dependence has to be shown in a *J/T* ^*2*^ *– 1/T* plot. Since *J*_*0*_ of PCMO and La_0.7_Sr_0.3_MnO_3_ have a linear dependence (Figure 12) in both models and give with 0.70 V (PCMO), 0.63 V (La_0.7_Sr_0.3_MnO_3_) (Schottky-model) and 0.72 V (PCMO), 0.65 V (La_0.7_Sr_0.3_MnO_3_) (pn-model) similar results for the built-in voltage, the temperature dependence does not exclude the description with one of the models. This value was compared with the built-in potential determined from the *1/C* ^*2*^ *– V* dependence in the reverse direction (Figure 12), which is 0.7 V for PCMO and 0.5 V for La_0.7_Sr_0.3_MnO_3_. Since both ways of determining the built-in voltage led to the same value for PCMO, Sawa et al. concluded that the model of a pn junction describes the PCMO-based junction.^33^ Since both ways lead to the same built-in voltage of 0.7 V, a work function of 4.6 eV can be derived for Pr_0.7_Ca_0.3_MnO_3_ while assuming 3.9 eV as the electron affinity of STO.

**Figure 12:**
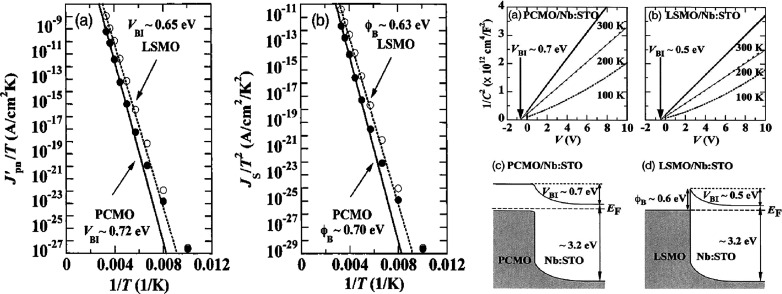
Built-in voltage determination from the Pr_0.7_Ca_0.3_MnO_3_ and La_0.7_Sr_0.3_MnO_3_ junction. Analysis of the temperature dependency of the *J*-*V* characteristics in the forward direction and of the temperature dependency of the 1/*C* ^2^ – *V* characteristics. Adapted and reprinted with permission from ref 33. Copyright 2005 by AIP Publishing.

Based on the same analysis, Asanuma et al.^32^ investigated the *1/C* ^*2*^ *–V* characteristics of the Nb:STO junction with CaMnO_3_, Pr_0.5_Ca_0.5_MnO_3_ and PrMnO_3_ (Figure 10). The work function increase by Ca hole doping by represented by an increase of the built-in voltage. This behavior is the same as for for La_1–*x*_Sr_*x*_MO_3_ (Figure 11b).

To investigate the influence of oxygen vacancies, the samples were first thermally reduced by annealing 1 h at 600 °C in ∼10^–7^ Torr and subsequently measured after annealing 1 h at 600 °C in 1 atm O_2_. As a result, the work function decreases by the electron doping from oxygen vacancy formation during annealing in reducing atmospheres, which is the opposite effect of Ca hole doping. The built-in potential decreases from 0.5 to 0.4 V (PrMnO_3_), 0.9 to 0.8 V (Pr_0.5_Ca_0.5_MnO_3_), and 1.5 to 1.3 V (CaMnO_3_). Assuming 3.9 eV as electron affinity of STO leads to the following work functions: 4.4 eV/4.3 eV (PrMnO_3_), 4.8 eV/4.7 eV (Pr_0.5_Ca_0.5_MnO_3_), and 5.4 eV/5.2 eV (CaMnO_3_) with oxygenated/reduced.

A value of 5.43 eV for the work function of Pr_0.7_Ca_0.3_MnO_3_ was found by Bi et al.^52^ analyzing the MOS capacitance characteristics of PCMO/ZrO_2_/SiO_2_/p-Si,^52^ where PCMO is serving as the metal. In this study, the flat band voltage of the MOS was determined for different SiO_2_ and ZrO_2_ thicknesses. It was shown that the influence of charges within ZrO_2_ and especially within SiO_2_ could be neglected. Further, the thickness dependence enabled Bi et al.^52^ to separate the influence of the interface charges and determine the work function.

The calculated work function of 5.43 eV of Pr_0.7_Ca_0.3_MnO_3_ and is much higher than the previously discussed work function of 4.896 eV from the Kelvin method^51^ or 4.6 eV from the Nb:STO junction characteristics.^33^ The authors discuss this issue using supportive DFT calculations, which investigate the influence of oxygen enrichment in the interface region. Their DFT calculation shows that oxygen enrichment at the PCMO interface increases the work function. This fits the previously discussed observation that oxygen vacancies reduce the work function. Bi et al.,^52^ therefore, explain the high work function with an increase of oxygen at the PCMO/ZrO_2_ interface.

An alternative explanation for the difference in work function could be the difference in crystalline quality at the measured interfaces caused by the difference in the growth conditions. In all three publications where lower work functions were found, PCMO was grown epitaxially by pulsed laser deposition on either LaAlO_3_ or Nb:STO at high temperatures of 800 °C,^51^ 700 °C,^33^ and 760 °C^32^ and with oxygen as the background gas. Bi et al.,^52^ in comparison, used RF-sputtering at 490 °C. Since the sputter deposition did not lead to full crystallinity, an additional thermal treatment of 5 min in a nitrogen atmosphere at 600 °C was used. However, it can be expected that the crystalline quality is better for epitaxial growth, causing a reduction of the work function.

### 2.7 Relative Permittivity of PCMO

The relative permittivity *ε*_*r*_ of PCMO influences the effect that charge has on the electrical potential and is, therefore, an important material property for band bending considerations. The higher the permittivity, the more charge will be shielded, and the lower its effect on the potential. The typical way to measure the dielectric constant is by impedance spectroscopy. Since the capacitance

2.23 $$C=\varepsilon_{r}\varepsilon_{0}\frac{A}{d}$$

of a parallel-plate capacitor with PCMO as dielectric increases with its relative permittivity, *ε*_r_ can be calculated by fitting the dielectric response with an equivalent circuit. The permittivity values of PCMO vary in the literature. Borgatti et al.^54^ performed impedance spectroscopy of PCMO-based memristive devices before and after forming and used *ε*_r_ = 110 for fitting the contribution from an oxygen-depleted Pr_0.5_Ca_0.5_MnO_3_ layer. Sheng et al.^55^ performed impedance measurements on Pr_0.5_Ca_0.5_MnO_3_ – Nb:STO junctions and determined a static dielectric permittivity of *ε*_r_ = 39 at room temperature. There exist several reports of a colossal dielectric response of 10^3^ to 10^5^ for PCMO at low temperatures. However, Biškup et al.^56^ claim that, quite probably, these findings do not originate from a change of the bulk properties but are caused by a contact capacitance. According to their opinion, the permittivity of bulk PCMO can considered to be *ε*_*r*_ = 30.

## 3 PCMO-Based Memristive Heterostructures

### 3.1 General Consideration on PCMO Based Switching Devices

Different types of stacks are used to fabricate PCMO-based memristive devices. This comprises epitaxially grown, polycrystalline, or amorphous PCMO.

In memristive stacks, PCMO is sandwiched between an active switching interface and an ohmic contact. Noble metals, such as Pt, that do not oxidize at the PCMO interface are used for ohmic contact. For the epitaxial growth of PCMO, SrRuO_3_ (SRO) can be used as a lattice-matched conductive bottom electrode.

The switching interface consists of an insulating, mixed conducting oxide and a reduced PCMO interface region. Some reports consider a space charge zone in the PCMO. The impact of the oxygen exchange on the current transport is most pronounced at the switching interface. Since the insulating oxides that are used have, as a result of the large bandgap, no significant amount of electrons in the conduction band, this layer is called tunnel oxide (TO) for simplicity in this section. A metal layer electrically addresses the tunnel oxide. Since the structure on the active side has some similarities with a MOS structure, it is further called MOP (metal/tunnel oxide/PCMO).

Different variants of MOP’s can be found in the literature. The stacks can be divided into stacks where the tunnel oxide is a result of a redox-reaction between the metal and the PCMO and cases where the TO is grown by a vapor deposition technique. In the latter case, often noble metals are used to avoid the formation of an additional oxide layer at the metal/TO interface.

In order to make use of the interface reaction to form the TO, metals that are easy to oxidize are chosen. Typical metals for this stack are Al, W, Ti, or Ta, as will be discussed in section 3.2. This stack can be prepared in two different ways. The oxidation of the metal by reduction of the PCMO can happen during the deposition, especially when the deposition happens at elevated temperatures. An electrical biasing step can further progress the interface oxide formation after the deposition, called the conditioning step, for which a positive bias is applied to the metal. The difference between the deposition of the TO and the formation of the TO by reduction of PCMO is that the amount of oxygen vacancies in the interface region of the PCMO is higher in the latter case. Further, the boundary between the tunnel oxide and the metal is due to the self-limitation of the interface reaction more continuous when the TO is formed by the interface reaction with PCMO.

### 3.2 Influence of the Gibbs Energy on Valve Metal Based Memristive Stacks

In this section, the formation of an interface oxide and its necessity for switching will be discussed. Therefore, the investigated metal/PCMO stacks can be categorized according to their tendency to form an interfacial oxide layer. This tendency can be assessed by comparing the standard formation energy Δ*G*_0_ of the different oxides, as shown in Figure 13. Δ*G*_0_ is given by

3.1 $$\Delta G_{0}=\Delta H_{0}-T\Delta S_{0}$$

while Δ*H*_0_ is the change in enthalpy and Δ*S*_0_ the change in entropy during the oxidation reaction. The temperature *T* is 298 K at standard temperature in the chosen literature.^57^ Δ*G*_0_ characterizes the change of the Gibbs free energy of a metal *M* during the oxidation reaction

3.2 $$xM\left(s\right)+O_{2}\left(g\right)\to M_{x}O_{2}\left(s\right)$$

**Figure 13:**
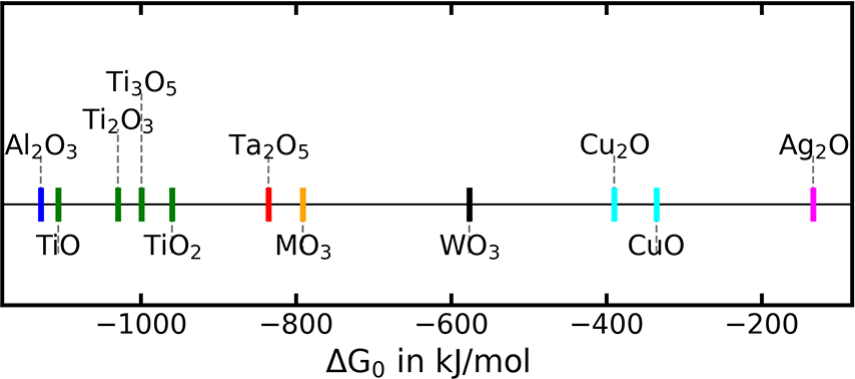
Change of Δ*G*_0_ of the metal during the oxidation reaction, normalized on the binding of one *O*_2_ molecule. All data have been taken from ref 57.

Δ*G*_0_ of metal oxides with different stoichiometries, the Δ*H*_0_ and Δ*S*_0_, the values taken from ref 57 have been normalized according to eq 3.2 to the change of free Gibbs energy of a metal during oxidation by one mole of oxygen molecules O_2_.

The redox reaction between PCMO and the metal can be divided into two partial reactions, namely, the reduction of the PCMO Δ*G*_red_ and the oxidation of the metal Δ*G*_0_. If the sum of the change in free Gibbs energy Δ*G*_red_ + Δ*G*_0_ < 0 is negative, the reaction can happen spontaneously. There are few papers that quantify Δ*G*_red_ of PCMO. Liao et al.^58^ claim Δ*G*_red_ to be 366 kJ/mol, by estimating it from the formation enthalpy of an intrinsic oxygen vacancy generation in La-doped BaTiO_3_,^59^ which can only be seen as a rough estimation. This reasoning based on the thermodynamic potential, however, has limitations since kinetic limitations such as diffusion barriers are not considered. Kinetic limitations can be overcome during the deposition of the metal, for example, due to the high energetic impact of the plasma during sputtering. Moreover, the literature values of Δ*G* are measured on crystalline bulk materials. Therefore, these values do not necessarily apply to amorphous interfacial oxides in the order of nm. However, for all metals shown in Figure 13 where Δ*G* > 500 kJ/mol, the formation of the interfacial oxides (Figure 14), has been shown experimentally, as will be discussed in the following text.

**Figure 14:**
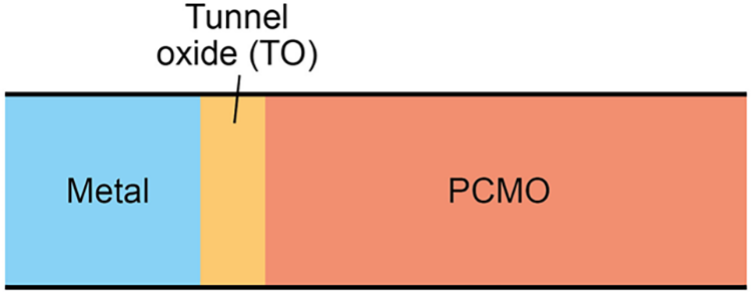
Interfacial tunnel oxide formation resulting in a MOP stack.

Gutsche et al.^60^ showed by XPS measurements that the combination of Al with PCMO forms an interlayer of native AlO_*x*_ at the interface between the PCMO and the deposited Al metal. They compared the X-ray photoelectron spectroscopy (XPS) of PCMO before and after the deposition of 7 nm Al (Figure 15a). The existence of the AlO_*x*_ layer, caused by the oxidation of the Al by reduction of the PCMO, has been shown by the appearance of Mn^2+^ shakeup peaks in the Mn 2p region after deposition. Furthermore, the increased intensity of an Al 2p oxide peak at 75 eV for a higher takeoff angle, corresponding to a higher depth sensitivity, shows the presence of the oxide layer.

**Figure 15:**
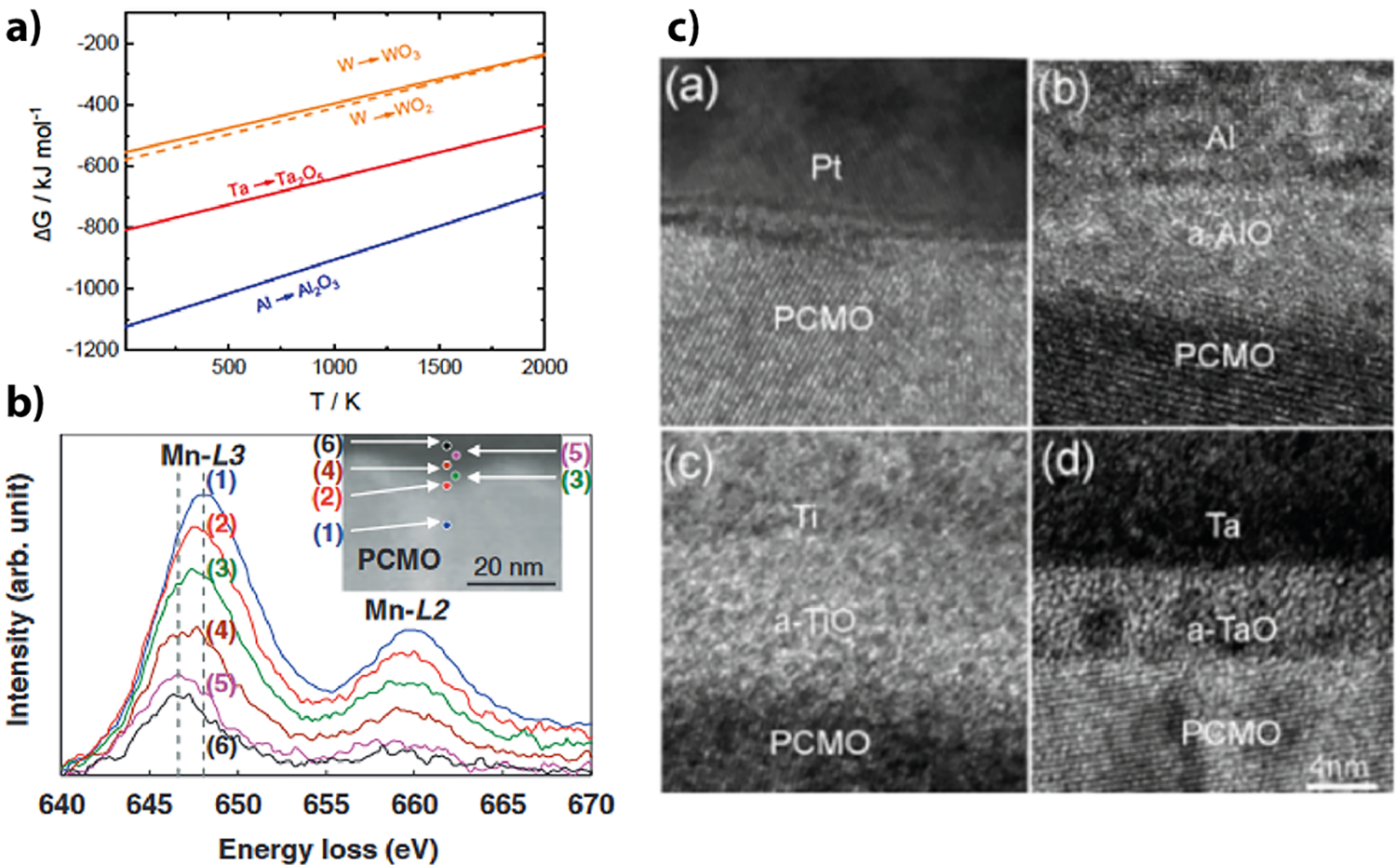
a) Mn^2+^ shakeup peaks after deposition Al deposition. Increased intensity of Al 2p oxide peak for higher takeoff angle. Adapted and reprinted with permission from ref 60. Copyright 2022 by the American Physical Society. b) Native TiO_*x*_ that has been formed by the deposition of Ti on top of a PCMO layer. Adapted and reprinted with permission from ref 61. Copyright 2008 by IOP Publishing Ltd. c) HRTEM image of the native oxide at the interface between the PCMO and Al, Ti, and Ta. No native oxide can be seen for the PCMO/Pt interface. Adapted and reprinted with permission from ref 58. Copyright 2009 by AIP Publishing.

Different papers report the formation of an interfacial oxide layer for oxidizable metals in contact with PCMO. Herpers et al.^54^ confirmed the formation of TiO_*x*_ at the interface using HAXPES. They further claimed that the first application of a voltage to the device leads to a homogenization of the TiO_*x*_ interface layer. This homogenization during the first cycle can also be found for PCMO/Al by Gutsche et al.^62^ Beside this, Shono et al.^61^ showed by TEM measurements the formation of a 10 nm amorphous-TiO_*x*_ layer between a PCMO and a Ti metal layer. Shono et al. showed by electron energy loss spectroscopy that the oxygen in the a-TiO layer stems from the PCMO layer, as can be seen in Figure 15b.

The impact of the interface oxide on the switching ability of PCMO-metal devices was demonstrated by Liao et al.^58^ They showed that only metals that form, such as Al, Ti, and Ta, that form an oxide at the interface exhibit resistive switching. Other studies also reported that Mo and W form an oxide interface layer in between the PCMO and the metal.^63^ In contrast, Laio et al.^58^ reported that more noble metals like Pt, Ag, Au, and Cu do not form an oxide at the PCMO interface, which is consistent with Figure 13. Laio et al.^58^ suggested that this native interface oxide is important for the resistive switching behavior of the PCMO/metal stack. They showed that without these oxide layers at the interface, no switching occurs, whereas the stacks with the native oxide layer show reliable resistive switching (Figure 15c). It can clearly be seen in Figure 16 that the PCMO/Ti, PCMO/Ta, and PCMO/Al stacks show a hysteresis, while the PCMO/Ag, PCMO/Au, PCMO/Pt, and PCMO/Cu stacks without the native oxide layer do not show any resistive switching hysteresis in the *IV*-curve. In Figure 17, it was shown that the initial resistance of the metal PCMO stack decreases with negative increasing free energy.

**Figure 16:**
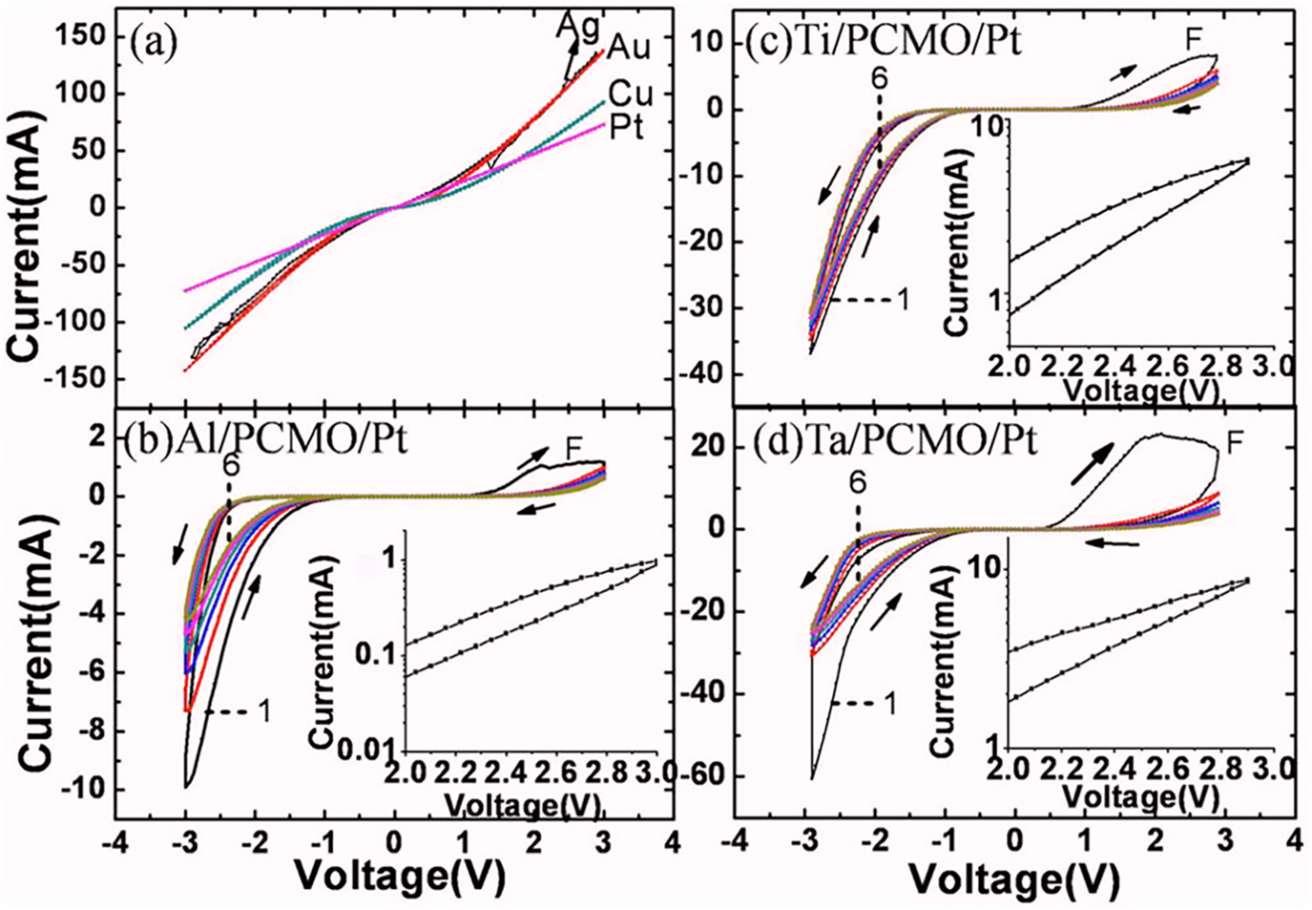
*I*–*V* characteristics of Pr_0.7_Ca_0.3_MnO_3_ junction with different types of metals. a) noble metals and b–d) non-noble metals. Adapted and reprinted with permission from ref 58. Copyright 2009 by AIP Publishing.

**Figure 17:**
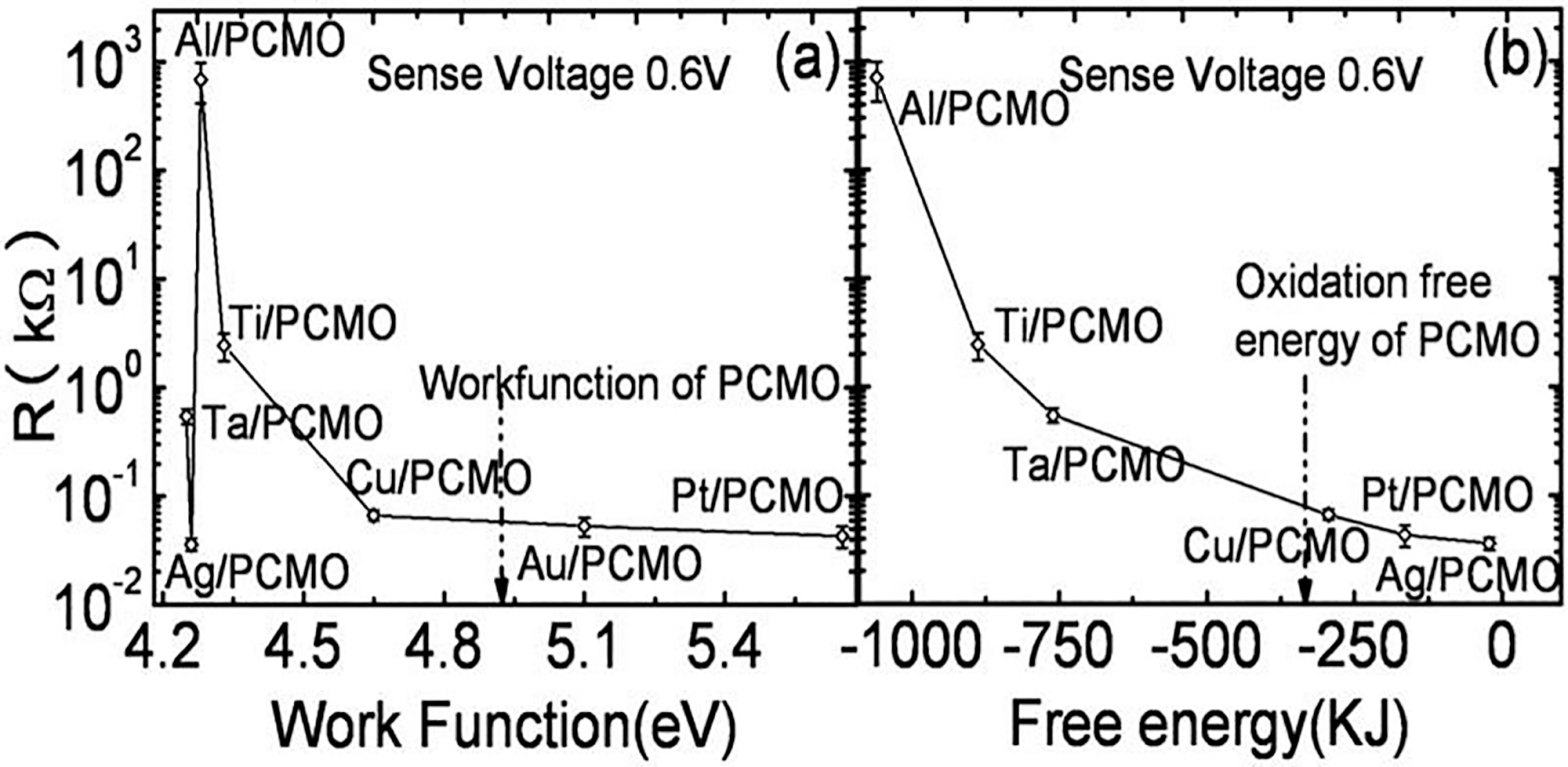
Comparison of work function and Gibbs energy for oxidation between PCMO and different metal contacts. Adapted and reprinted with permission from ref 58. Copyright 2009 by AIP Publishing.

This can be attributed to stronger oxidation at the interface. Liao et al.^58^ assumed oxidation free energy of PCMO is marked with a dashed line and all the stacks that show resistive switching are at higher negative energy, see Figure 17. This further confirms that the oxidation of the metal at the interface and the reduction of the PCMO is crucial for the resistive switching process.

The question arises of whether the oxidation of the metal or the reduction of the PCMO is more important for the resistive switching process. Different studies show that a directly deposited oxide layer also leads to resistive switching behavior. For example, PCMO stacks with TaO_*x*_, WO_*x*_ and Al_2_O_3_ show resistive switching.^64^ In these cases, one can assume that the PCMO layer is not reduced during the deposition. We can, therefore, conclude that an oxide layer seems to be crucial for resistive switching and not the reduced PCMO interface layer. Furthermore, it was also shown that PCMO, in combination with TiN, shows resistive switching.^65^ Also, here, an interfacial TiN_*x*_O_*y*_ layer gets formed and modulated during switching.^66^ The crucial role of the interface oxides will be discussed later in section 3.4.

### 3.3 Space Charge Region in the MOP Structure

Since the active switching MOPstructure (Figure 18) has similarities with an MOS, a similar band diagram can be expected.

**Figure 18:**
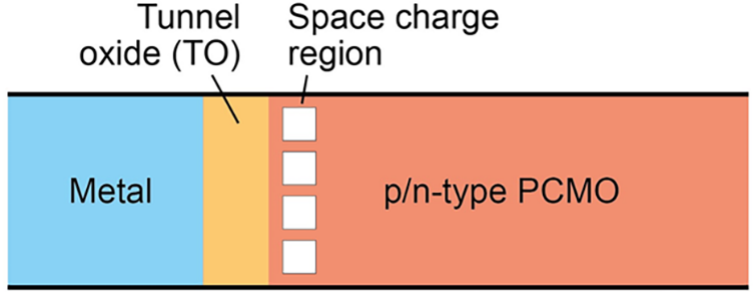
MOP stack with significant space charge region.

A built-in potential *V*_b_ = |Φ_P_ – Φ_M_| is caused by the work function difference between the metal Φ_*M*_ and the PCMO Φ_*P*_. Depending on the built-in potential, a space charge forms, with a narrow space region in the metal and a broader space charge region in the PCMO and, thus, a linearly tilted band in the tunnel oxide. The depth of the space charge region in PCMO is under discussion since it has a higher charge carrier density ρ than typical MOS. The thickness *d* of the space charge region can be estimated by the equation which is commonly used for the thickness of the depletion zone width in MOS^67^

3.3 $$d=\sqrt{\frac{2V_{b}\varepsilon_{P}\varepsilon_{0}}{e\rho }}$$

where *ε*_*P*_ is the permittivity of PCMO, *ε*_0_ the vacuum permittivity and *e* the elementary charge. To estimate the thickness of the space charge region, the thickness is plotted in (Figure 19) in dependence on a work function difference *V*_*b*_ up to 2 eV. The density of states *ρ*_*eg*_ in the e_g_ band is formally

3.4 $$\rho_{eg}\left(x\right)=\frac{\rho_{M}\left(x\right)}{M\left(x\right)}N_{A}$$

given by the molar mass *M* (*x*)­

3.5 M(x)=(1−x)M[Pr]+xM[Ca]+M[Mn]+3M[O]

and the mass density *ρ*_M_(*x*) of PCMO in dependence on the Ca doping *x*. M­[Pr] = 141 u, M­[Ca] = 55 u, M­[Mn] = 40 u, and M­[O] = 16u are the used molar masses of the single elements. To get an estimation for different scenarios of doping, the density of states *ρ*_eg_ was calculated for *x* = 0.1, 0.5, and 0.9 using mass densities of *ρ*_M_(0.9) = 4.83 g/cm^3^, *ρ*_M_(0.5) = 5.79 g*/*cm^3^ and *ρ*_M_(0.1) = 6.59 g/cm^3^ taken from Pithan et al.^9^ This results in density of states *ρ*_eg_ of *ρ*_eg_ (0.1) = (1.7 * 10^22^/cm^3^, *ρ*_eg_(0.5) = (1.8 * 10^22^/cm^3^ and *ρ*_eg_(0.9) = (1.9 * 10^22^/cm^3^ Since the decrease of the molar mass with Ca doping is compensated by an increase in the mass density, the formal density of states remains fairly constant over the doping range. Multiplying with the amount of doping results in the formal carrier density of holes *ρ*_p_ and electrons *ρ*_e_ for the different doping with *ρ*_p_ (0.1) = 1.7 * 10^21^/cm^3^, *ρ*_n__*/*p_ (0.5) = 9.0 * 10^21^/cm^3^ and *ρ*_n_ (0.9) = 1.9 * 10^21^/cm^3^.

**Figure 19:**
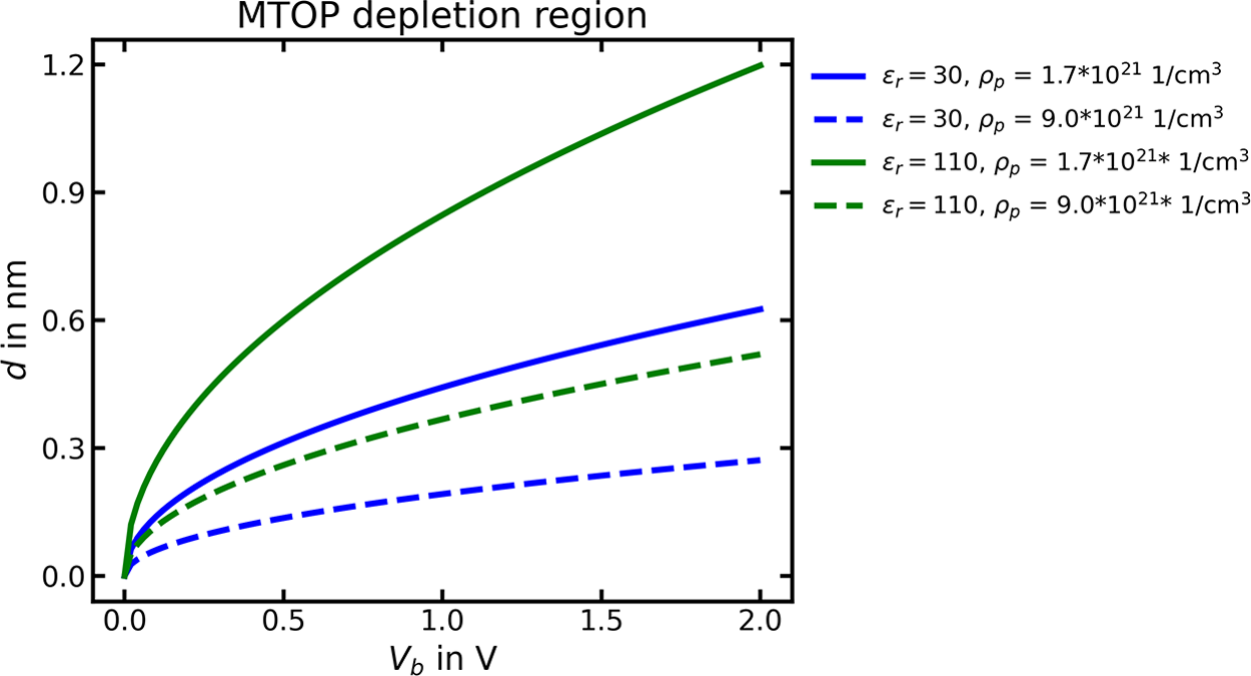
Plotting of the approximated size of the depletion region in dependence on the work function difference for different permittivity values and charge carrier concentrations.

Since *ρ*_p_ (0.1) and *ρ*_n_ (0.9) are quite similar only *ρ*_p_ (0.1) and *ρ*_n/p_ (0.5) have been used for the calculations shown in Figure 19.

The relative permittivity of PCMO had been chosen according to section 2.7, namely *ε*_r_ = 30 is used to represent PCMO and *ε*_r_ = 110 is used to consider a possible increase of permittivity after reduction in the interface layer.

The range of possible built-in potentials within MOP stacks can be considered by using the boundary values of the work function (section 2.6) of 5.4 eV (Pr_0.7_Ca_0.3_MnO_3_) for a junction with p-type PCMO and 4.8 eV (Pr_0.5_Ca_0.5_MnO_3_) for a junction with n-type PCMO. For the case of p-type PCMO, a build-in potential of up to 1.2 eV can be achieved by combining it with a metal with a low work function, like Ag, with around 4.2 eV (Figure 17). For the case of n-type PCMO, one can achieve a difference of 0.9 eV by combining it with a metal with a high work function, like Pt, with around 5.7 eV (Figure 17). Therefore, in Figure 19 we used work function differences of up to 2 eV.

Considering Figure 19 for the more realistic value of *ε*_r_ = 30, it is unlikely that the size of the space charge region exceeds a few Ångström. For the case of high permittivity *ε*_r_ = 110and low carrier charge densities the space charge region can spread up to 1 nm into the material (Figure 19).

The narrow width of the space charge region in Pr_0.66_Ca_0.34_MnO_3_ due to the high charge carrier density has also been shown by simulations of the junction Pr_0.66_Ca_0.34_MnO_3_/ SrTi_1–y_Nb_*y*_O_3_ (y = 0.002), which results in a space charge region of around 0.2 nm in PCMO. Combined EBIC measurements confirmed the results of the simulation indirectly by measuring the simulated space charge region within STO. They excluded the space charge region in the PCMO as being of a few nm in size without giving a precise value in the single nm range because of the limit in the resolution.^68–71^

However, Gutsche et al.^60^ measured the space charge region at the Al/AlO_*x*_/Pr_0.7_Ca_0.3_MnO_3_ interface by comparing X-ray photoelectron spectroscopy (XPS) measurements of PCMO before and after the deposition of 7 nm Al. As expected for the work function difference, the band down bending can be seen by a shift of 0.9 eV of the Pr 3d 5/2 region to higher binding energies after the deposition of Al (Figure 20a). The band diagram of the Al/AlO_*x*_/PCMO MTOP structure is drawn in Figure 20. The negative space charge in the PCMO, with an electrostatic potential of 0.9 V, causes a linear field over the AlO_*x*_ with a total potential difference of 0.9 V. Interestingly, this increase in the binding energy of the Al 2p oxide was observed. The measured peak is around 75 eV (Figure 15a), while the literature value of the peak position for Al 2p 3/2 of corundum is around 74.1 eV.^60^

**Figure 20:**
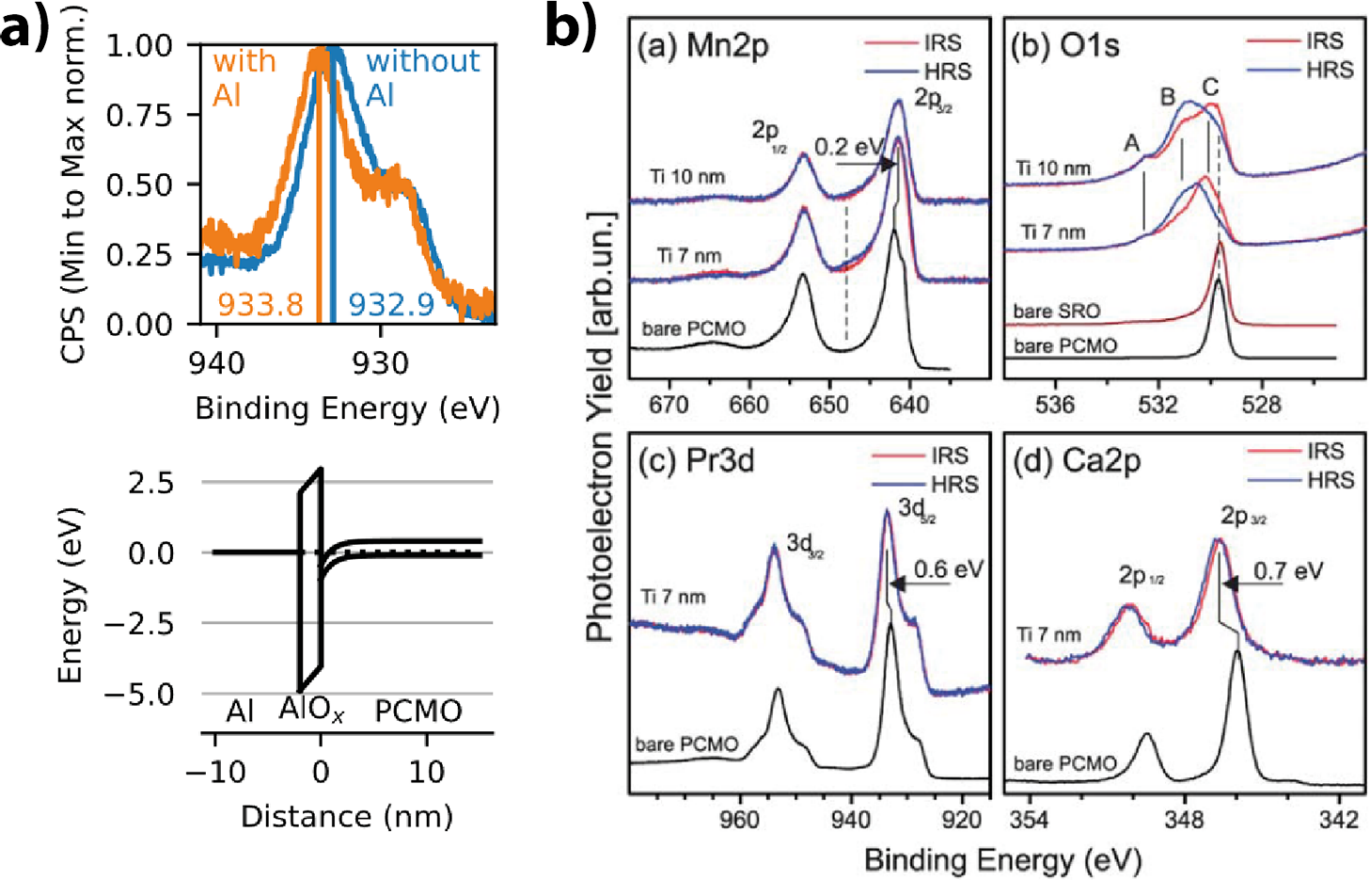
a) Shift in the Pr 3d 5/2 peak from the band down bending and concluded band diagram for the PCMO/AlO_*x*_/Al system. Adapted and reprinted with permission from ref 60. Copyright 2022 by the American Physical Society. b) Core level shift in PCMO between bare PCMO and PCMO after Ti deposition. Adapted and reprinted with permission from ref 72. Copyright 2009 by Royal Society of Chemistry.

Borgatti et al.^72^ measured the space charge region for the system PCMO/TiO_*x*_/Ti by the shift to higher binding energies of 0.7 and 0.6 eV in the Ca 2p and Pr 3d core level region, respectively (Figure 20b). This is expected for the combination of PCMO with the low work function metal Ti 4.3 eV (Figure 17).

Since in both publications Gutsche et al.^60^ and Borgatti et al.^72^ an interfacial oxide was created by the reduction of the PCMO, the measured space charge region could even be increased, by the combined change of band gap and work function of reduced PCMO as discussed in section 4.4.2. Also a possible increase of permittivity and reduction of charge carrier density, as discussed in section 2.5, 2.7, 4.3.4, and 4.4.2, could increase this effect.

It is evident, from spectroscopy, that the space charge region exists and can be measured, but since the region can be expected to be very narrow (Figure 19), the question arises if the space charge region has an influence on the current transport at the interface. A direct way to investigate the influence is to investigate metal/PCMO junctions (Figure 21), which form a rectifying Schottky junction, in the case of a significant space charge region. In contact with a noble metal with a lower work function than p-conducting PCMO, the forward direction of the Schottky junction would be at negative voltages with respect to the metal contact. The opposite (positive) voltage would be expected as the forward direction in the case of a high work function metal electrode and an n-conducting PCMO.

**Figure 21:**
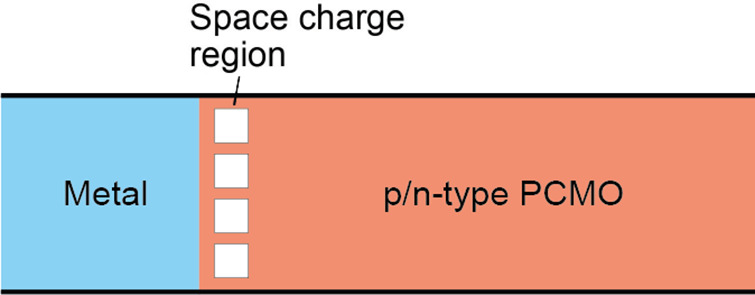
Schottky junction at PCMO/metal contact, assuming a significant space charge region.

Borgatti et al. observed symmetric *IV*-curves for the contact between Pr_0.5_Ca_0.5_MnO_3_ and the high work function metal Pt as well as for the contact with the low work function Ti before electronic forming (Figure 22a).

**Figure 22:**
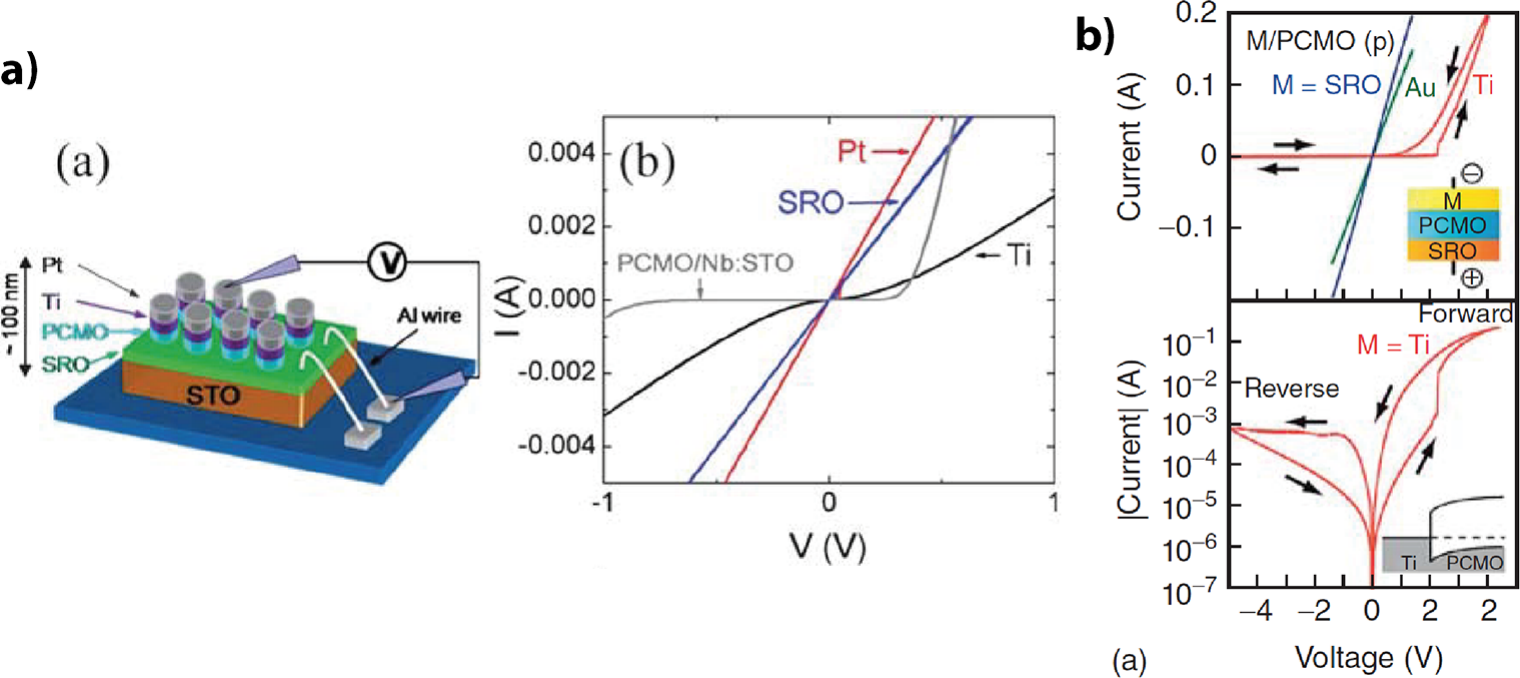
a) IV curves for PCMO devices (structure shown in a) with different top electrodes. To analyze the junctions’ characteristics, the shown voltage is the voltage applied to the top electrode. The junction with the Ti electrode is without previous forming treatment by electric biasing and has, therefore, an interfacial Ca-TiO_*x*_ oxide layer, which is less influential to the electric characteristic. Adapted and reprinted with permission from ref 72. Copyright 2009 by Royal Society of Chemistry. b) Measurement of the M/PCMO/SRO junction for three different contacts M with different work functions (Ti 4.1 eV, Au 5.1 eV, and SRO 5.3 eV). Adapted and reprinted with permission from ref 5. Copyright 2008 by Elsevier.

Interestingly, in contrast to Borgatti, Sawa et al.^5^ (Figure 22b) observed a rectification in the *IV*-curve of PCMO-Ti junction. Probably for Sawa’s measurements a thicker interfacial oxide layer was formed and the measured asymmetry results from the current transport across a tunnel junction with different work functions of the contact electrodes, as explained in section 3.4.1.

The same observations as Borgatti et al. was been made by Liao et al.^58^ by combining PCMO with the metals Ag (4.26 eV), Au (5.1 eV), Cu (4.65 eV) and Pt (5.7 eV). All these junctions have symmetric *I–V* characteristics (Figure 16).

It can, therefore, be concluded that the influence of the space charge region on the current transport of the metal/PCMO junction without an interfacial oxide layer can be neglected. This raises the question of whether this is also the case for transport over the MOP stack. Here, the charge transport over the tunnel oxide dominates the resistance, as can be clearly seen for PCMO/TO structures in which the tunnel oxide thickness is controlled by the deposition time (Figure 24a). There is a resistance difference of around 1 order of magnitude between the stack Pt/PCMO/ZrO_2_/Pt with PCMO and the MIM stack with only the tunnel oxide (MIM). Meyer et al. assigned the resistance difference to the space charge region in PCMO.^5^ How the current transport over the MOP stack can be described and what influence the space charge region has will be discussed in the next section.

### 3.4 Current transport across the MOP

#### 3.4.1 Direct Tunneling

The electronic conductivity of the PCMO based devices is dominated by the conductivity over the tunnel oxide, as is evident from Figure 24a.^73^ To describe the conduction over the tunnel oxide, different models can be used.

Herpers et al.,^54^ as well as Arndt et al.^74^ used the tunnel model of Simmons,^75^ which has been developed for direct tunnelling through an insulating barrier (Figure 23). Simmons^75^ describes the asymmetry of the tunnelling current through an insulating current for two contact metals with different work functions.

**Figure 23:**
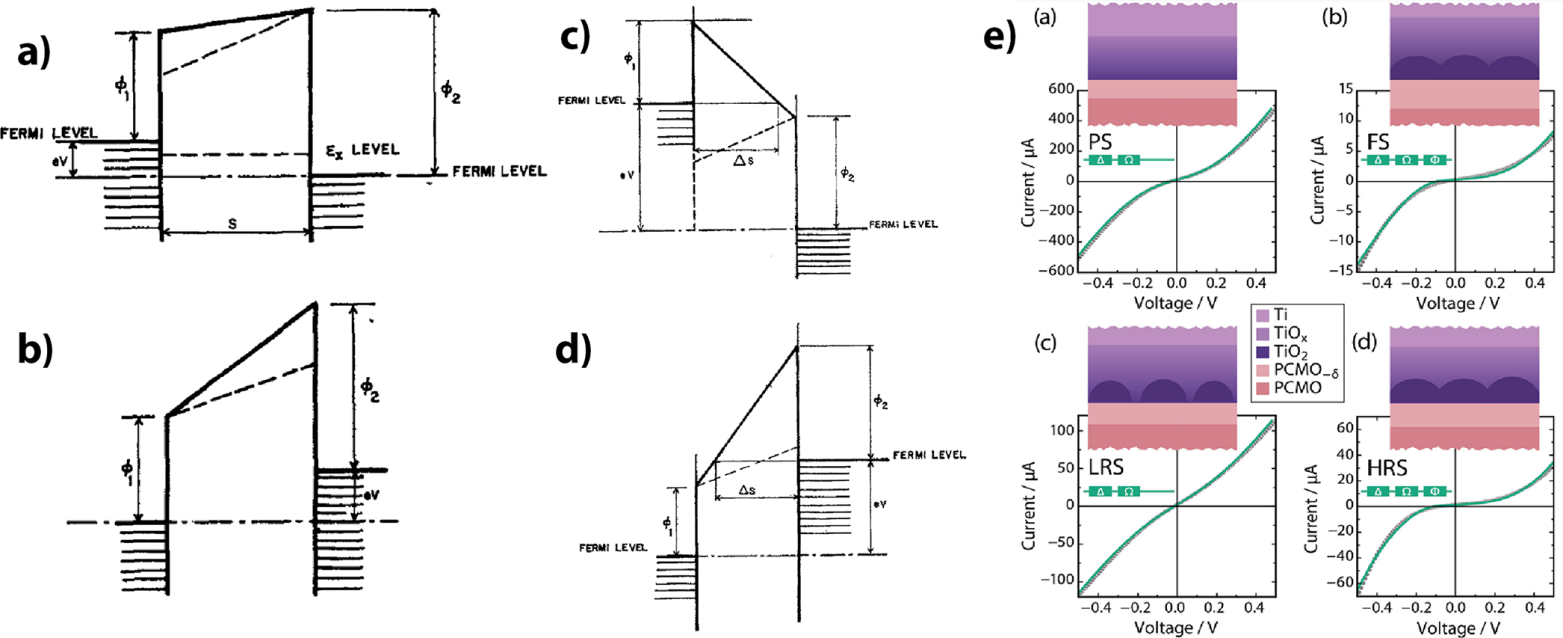
In a) and b), the tunneling scenario relative to the tunnel barrier’s low applied voltage is shown. Here, biasing the low work function metal with a positive bias b) leads to a higher tunneling current. The right panelss c) and d) show the scenario for high applied voltages, which leads to an effective triangular barrier. Here, biasing the electrode with low work function negative c) gives the higher tunneling current. a) until d) all adapted and reprinted with permission from ref 75. Copyright 2004 by AIP Publishing. e) Fitting of the current characteristic in the low readout voltage regime of the PCMO/TiO_*x*_/Ti device by two circuit elements (e.a) pristine device, e.c) device in LRS) and three circuit elements (e.b) formed device and e.d) device in HRS). The two circuit elements are a constant resistance and the PCMO polaron hopping. The third resistance is the Simmons tunneling over the tunnel barrier. Adapted and reprinted with permission from ref 54. Copyright 2014 by John Wiley and Sons.

The Simmons model distinguishes between low and high applied voltage regimes. Low means that the voltage drop across the tunnel oxide is lower than the average height of the tunnel barrier $\phi =\frac{\phi_{2}-\Phi_{1}}{2}$ . In this voltage regime, the tunnelling barrier is trapezoidal, and the tunnelling current is higher for positive biasing at the low work function electrode (Figure 23a,b). High voltage means that *V* > ϕ. In this case, the tunnel barrier becomes effectively triangular (Figure 23c*,*d), and the tunnelling current is controlled by the work function of the metal, which is negatively biased. Thus, the tunnelling current is higher when the negative voltage is applied in the low work function metal.

This asymmetric behavior is the same as would be expected for the forward direction of a Schottky barrier with PCMO as p-conducting material and a low work function metal. This must be considered, if one wants to conclude from the asymmetry of the *I–V*-curves to the existence of space charge.

Simmons^75^ developed two analytical descriptions to calculate the tunnelling current. The simpler form does not include the barrier lowering by the image force effect. Herpers et al.,^54^ as well as Arndt et al.,^74^ used this form to describe the tunnelling current across the TO. It converges to the Fowler-Nordheim tunnelling when the voltage across the TO is larger than the difference between the two work functions. Then, the tunnelling current is described by

3.6 $$J=\frac{BE^{2}}{\phi }e^{-\lambda \phi^{3/2}/E}$$

where *E* = *V*/*d* is the electric field, *V* the applied voltage, *d* the TO thickness, *B* = 1.1*e* ^3^/(4*πh*) and $\lambda =23\pi \sqrt{m^{*}}/\left(6he\right)$ with the effective mass m* within the tunnel oxide.

Arndt et al.^74^ described the current transport of Pr_0.48_Ca_0.52_MnO_3_/YSZ/Pt devices and showed an exponential dependence of the tunnelling current on the tunnel oxide thickness (Figure 24b) as predicted by equation 3.6.

**Figure 24:**
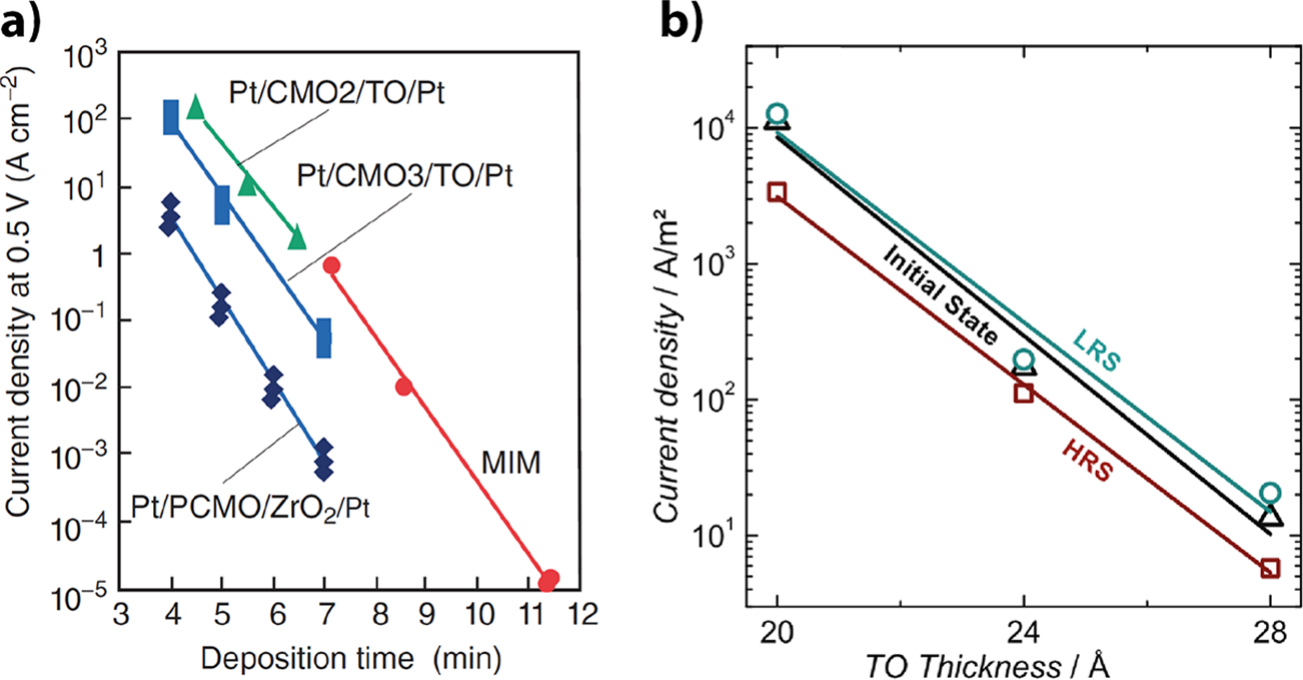
a) Thickness dependence of the current transport of PCMO device with different ZrO_2_ tunnel oxide thicknesses. In comparison with other higher conducting metal oxides (CMO2 and 3) and a stack without CMO. Adapted and reprinted with permission from ref 73. Copyright 2016 by John Wiley and Sons. b) Exponential dependency of the current density of the PCMO/YSZ/Pt system from the YSZ thickness. Taken from ref 74.

Herpers et al.,^54^ fitted the current–voltage curves of the PCMO/TiO_*x*_/Ti stack in the read-out voltage regime in four different states. In the pristine state (PS) and in the LRS, the TiO_*x*_ is assumed to be so leaky that a transport via the conduction band can be assumed, which results in symmetric *I–V*-curves (Figure 23e.a+e.b). After the first biasing, which induces the formation of a TiO_2_ tunnel barrier (FS) and in the HRS, the current transport can be described by the Simmons model, which causes asymmetric *I–V* curves in both cases (Figure 23e.b+e.d). The asymmetry in the HRS could even be more pronounced if the band gap and work function changes for reduced PCMO, as discussed in section 4.4.3.

However, the application of the Simmons model for the transport over a MOP stack has several limitations. It completely ignores conduction through trap states in the band gap, and it incorrectly assumes PCMO is a metal. Therefore, it does not consider the influence of the density of states near the Fermi level or the impact of the influence of a space charge region. Further, direct tunneling cannot explain the high current levels often observed for tunnel oxide thicknesses above 3 nm.

#### 3.4.2 Trap Assisted Tunneling in the TO

Oxygen vacancies can form defect states^60,76^ within the band gap of the tunnel oxide. If these defects states lie above the Fermi level, they are considered as trap states and must be included into the transport mechanisms across the tunnel oxide (Figure 25).

**Figure 25:**
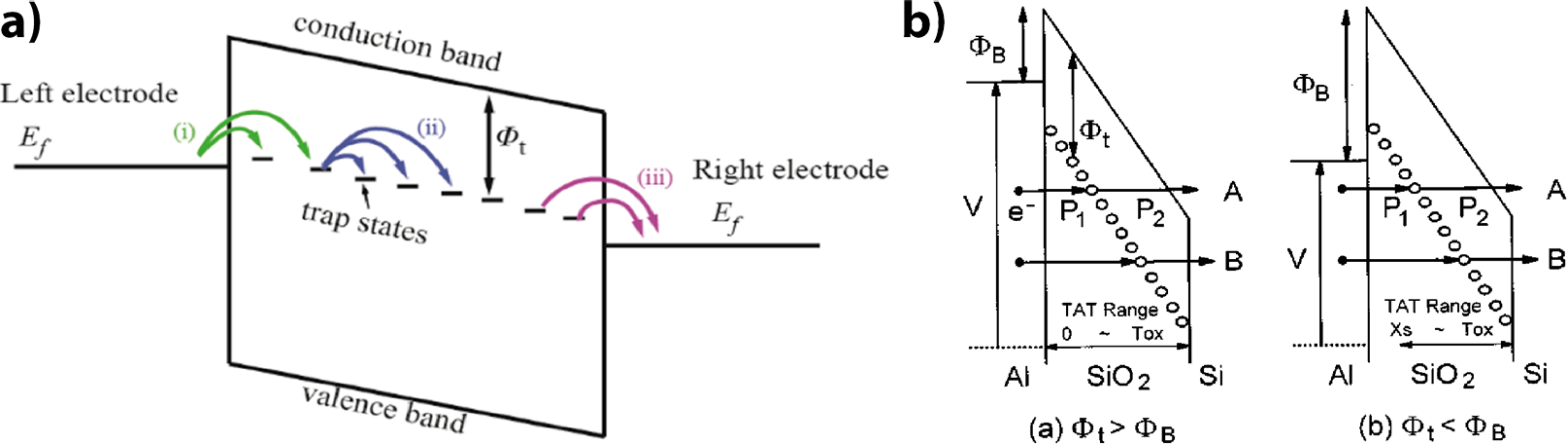
a) Schematic drawing of the elementary tunneling steps during trap-assisted tunneling. Taken from ref 76. b) Two scenarios of trap-assisted tunneling. b.a) if the tunnel barrier is smaller than the trap level depth. b.b) if the trap level depth is smaller than the tunnel barrier. In both scenarios, two different tunneling processes exist. A, which is tunneling through a triangular barrier and B tunneling through a trapezoidal barrier. Adapted and reprinted with permission from ref 77. Copyright 1999 by AIP Publishing.

Funck et al.^76^ compared different transport models and the role of defect states. Trap-assisted tunneling (TAT) consists of up to 3 steps (Figure 25a).^76^ In the first step (i), the electron tunnels from the conduction band of one material into a trap or defect state within the band gap of the tunnel oxide. Then, depending on the thickness of the TO in an optional (ii) second step, the electron can tunnel between different trap states. In the final step (iii), the electron tunnels out of the tunnel oxide into the second material. The mathematical description of this process depends highly on the exact structure of the energetic landscape and is, therefore, not covered by a single analytical expression.

This process is considered e.g. by Mayer et al.^5,73^ for the transport across the Pt/ZrO_2_/PCMO stack, for which an exponential dependence on the tunnel oxide thickness is observed (Figure 24a).

As reviewed by Funck et al.,^76^ an often-used equation for TAT is given by Phon et al.,^77^

3.7 $$J\propto e^{-k\phi_{t}^{3/2}/E}$$

where *Φ*_*t*_ is the in Figure 25b shown defect level and $k=4\sqrt{2em^{*}}/\left(3\hbar \right)$ with m* as the effective mass in the tunnel oxide. This equation describes the simplified situation in which the tunneling takes place over only one trap state, with the trap depth *Φ*_*t*_ smaller than the tunneling barrier *Φ*_B_ (scenario (a) in Figure 25b. Further, it is assumed that the field is high, so tunneling process A (depicted in Figure 25b) across the triangular barrier dominates. This equation shows the same exponential dependence with the tunnel oxide thickness of the Fowler-Nordheim equation for direct tunnelling. Therefore, just from finding an exponential thickness dependence (e.g., Mayer et al. (Figure 24a) and Arndt et al. (Figure 24b)), it cannot be discriminated if the mechanism is trap-assisted or direct tunneling.

### 3.5 Space-Charge Limited Current

Space-charge limited current (SCLC) is a conduction mechanism, which is based on the space charge distribution of the equilibrium flow of excess charge carrier in material. This model was first developed to explain the current flow in vacuum and was then adapted to describe electric transport in insulators with a certain electronic mobility by the so-called Mott–Gurney law for SCLC.^76^ This transport in an insulator gives a *I ∝ V* ^*2*^ characteristic, which is often used to identify SCLC as conduction mechanism in *I–V* curves.

However, applying SCLC current to a material, certain requirements have to be fulfilled, such that the transport in the material happens by excess charge emitted by the electrodes. Only finding an interval with *I ∝ V* ^*2*^ characteristic in the *I–V* loop is not enough, as nonlinearity of the *I–V* characteristic can have multiple reasons.

The application of the SCLC model to PCMO, which is suggested in numerous papers in the literature,^78–82^ should be considered with caution, as the requirements are not fulfilled. The SCLC model assumes, as reviewed by Funck et al.,^76^ assumes a free excess electron in the material whose charge distribution limits the current. But PCMO does not require the injection of excess charge for electronic conduction, since it has due to the high doping concentration large charge carrier densities. In addition, the *e*_g_ electron does not behave like a free electron, as its mobility is severely restricted and field dependent, as discussed in section 2.4. Nonlinearities in the *I–V* loop can be explained by the nonlinearity of the polaron hopping.

The conduction based on SCLC can be further modified by the electronic traps in the material. There are several ways to respect them. Funk et al. reviewed, that traps can be reflect by a change to an *I ∝ V* ^*α*^ characteristic, where α varies with the trap density. However, other approaches are to reflected traps by a factor θ to the current *I∝ θI*_trap free_.

## 4 Switching

### 4.1 Area-Dependent Switching

In contrast to filamentary switching VCM devices, the current transport in area-dependent devices is homogeneous across the whole device area in both HRS and LRS. As a result of this, the current in both states scales with the device area. The difference to the filamentary type of devices is most pronounced in the LRS, where the current is dominated by transport within the filament.^4^ A common way to validate the area dependence is to plot the device resistance in both resistive states against the device area *A* in a double logarithmic plot, as exemplary shown in Figure 26 for AlO_*x*_/PCMO, TaO_*x*_/PCMO and WO_*x*_/PCMO. A linear fit of the device resistance with a slope of −1 verifies that the resistance is scaled by *1/A*. Moon et al.^83^ showed for Mo/PCMO and Park et al.^84^ for TiN_*x*_/PCMO that the area dependence scales down to devices of 150 nm diameter (Figure 27). A limit to the scaling behavior has not yet been shown. Public statements^85^ of the company 4DS Memory, which claim to have achieved 60 nm device sizes and are aiming for 20 nm device sizes, support that further scaling of PCMO-based devices may be possible.

**Figure 26:**
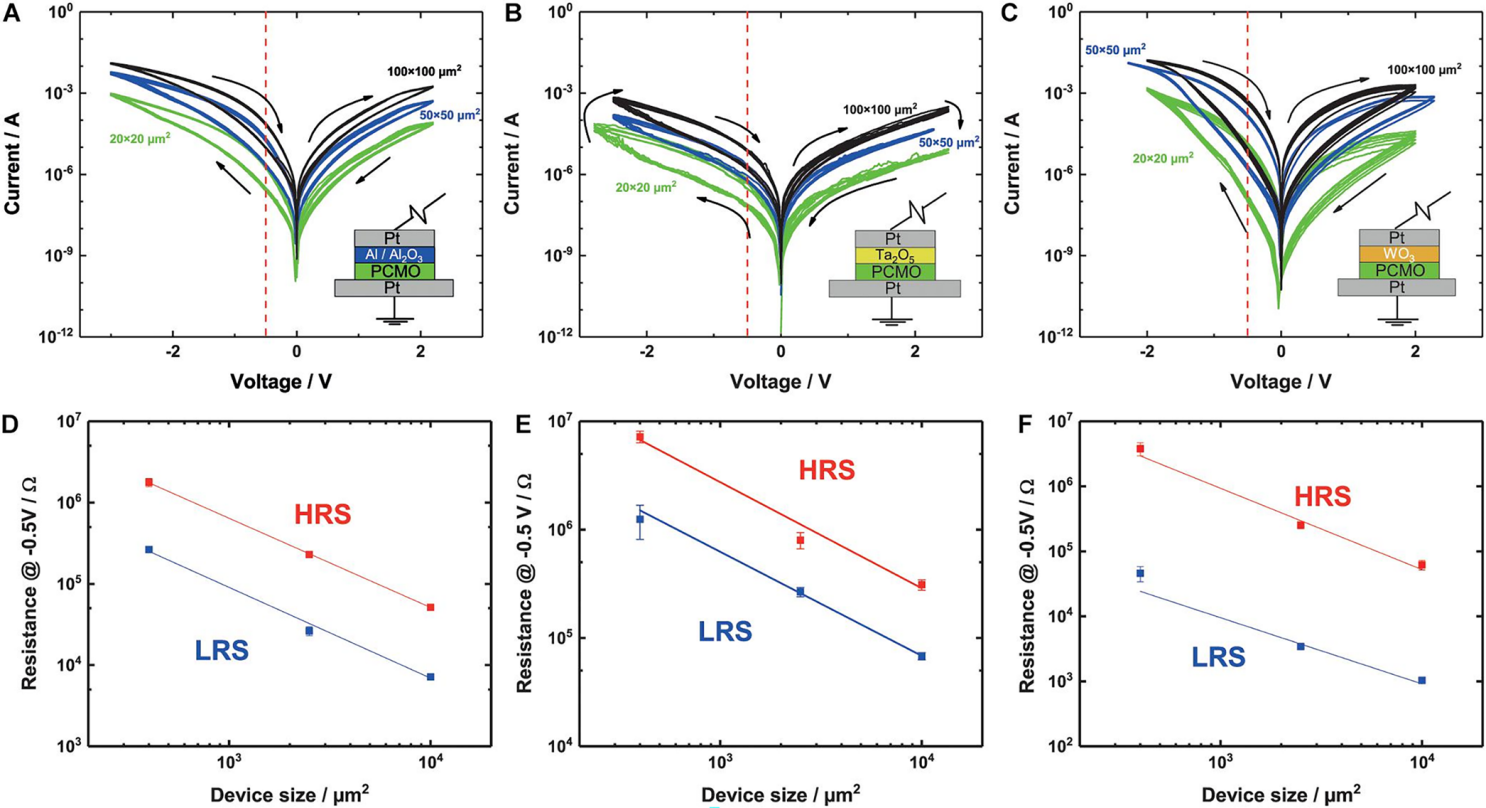
Switching curves of PCMO based devices with A) AlO_*x*_, B) TaO_*x*_ and c) WO_*x*_ and different pad sizes of 100 × 100, 50 × 50, and 20 × 20 μm.^2^ D) to F) show the area dependence of both resistive states, for a readout at −0.5 V, and their linear fit, with slopes close to −1 Ω/μm^2^. The values for HRS/LRS are D) −1.09 ± 0.01/–1.12 ± 0.07, E) −0.98 ± 0.18/–0.95 ± 0.05 and F) −1.25 ± 0.18/–1.02 ± 0.22. All values and figures are taken from ref 62.

**Figure 27:**
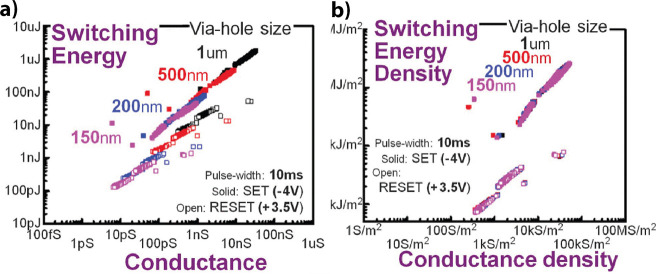
a) Scaling of switching energy with conductance for devices of different sizes. b) Scaling of switching energy density with conductance density for devices of different sizes. Both figures are taken from ref 83.

Moon et al.^83^ investigated the dependence of switching energy on conductance level for devices of different sizes and verified the expected decrease in switching energy with conductance (Figure 27a). They also verified the scaling of switching energy with device size by examining the dependence of switching energy density on conductance density (Figure 27b). Moon et al.^83^ suggested that for 25 nm devices with 10 ns switching times, switching energies in the order of femtojoules may be possible. The potential to achieve these low switching energies at small device sizes could give PCMO-based devices an significant advantage over filamentary devices. Moreover, the high resistance values of PCMO-based devices even in the LRS are advantageous for the implementation of these devices in large crosss-bar arrays since parasitic effects caused by the line resistances might be circumvented.

In the ideal case of area-dependent switching, the atomic arrangement is homogeneous in every cross-section along the entire stack and does not change during the application of an electric field. Therefore, also the electric field and the resulting ionic processes are homogeneous across the entire interface. However, interfaces of deposited layers always have a certain degree of roughness and imperfection. The electric field might also be inhomogeneous, as can be seen in the simulation shown in Figure 28a. Here, the continuity equation for the electric current has been solved for a stack of 20 nm metal/5 nm tunnel oxide/30 nm PCMO/20 nm metal. The inhomogeneity simulated here is a general inhomogeneity in the resistance of the tunnel oxide. For that purpose, the TO is described by a checkerboard pattern with single fields of 20 nm x 20 nm size. The PCMO is included by an arbitrary conductivity of 10^3^ S/m, and one subset of the tunnel oxide areas is considered by a fixed low conductivity of 1 S/m. The conductivity varies from Figure 28 a) to d) from the second subset of the TO pattern, with decreasing conductivity of a) 10^5^ S/m, b) 10^3^ S/m, c) 32 S/m, and d) 1 S/m. If the resistance of the TO dominates (seen c) and d)), the whole field is located in the tunnel oxide. As soon as the resistance of the TO becomes similar to the resistance of the PCMO b), or is even lower a), the field strays into the PCMO. This increase of resistance from a) to d) relates to the change from a metal to an oxide during the conditioning for metal/PCMO stacks, as discussed in section 3.2.

**Figure 28:**
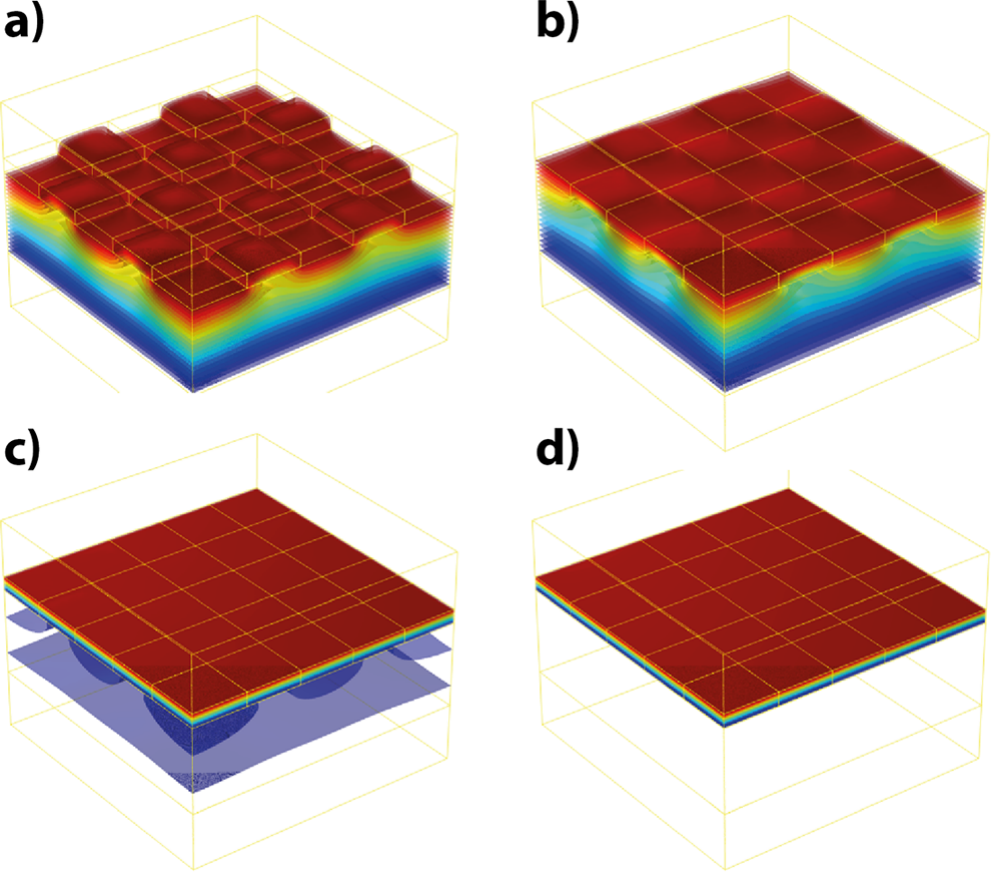
Equipotential lines color-coded of the electric potential distribution (red 2 V two blue 0 V) for a tunnel oxide with inhomogeneous conductivity and PCMO with a fixed conductivity of 10^3^ S/m. The inhomogeneities of the conductivities in the tunnel oxide are distributed in a checkerboard (20 nm × 20 nm) pattern. Where the insulation area has a constant conductivity of 1 S/m, and the variable area has a conductivity of a) 10^5^ S/m, b) 10^3^ S/m, c) 32 S/m, and d) 1 S/m. The conductivity of the metal contacts is 10^6^ S/m, and the applied voltage is 2 V.

The simulations further show that the stray of the field into the PCMO in the area where the TO is high conducting becomes shielded by the field confined in the low resistive areas of the TO because of the continuity of the field lines. Thus, it can be concluded that this effect is stronger if the area of low conductivity in the tunnel oxide is less granular.

Since both field and resistance inhomogeneities, as well as surface inhomogeneities, can result from the device fabrication, there has to be feedback mechanisms that compensate for the field inhomogeneities through the movement of oxygen. Otherwise, the area-dependent configuration would not remain stable over many switching cycles.

The most evident case of a feedback mechanism is the formation of the tunnel oxide at the valve metal-oxide interface during the conditioning process, as simulated by the resistance increase from a) to d) in Figure 28. Here, the formation of the tunnel oxide is caused by the movement of oxygen anions from PCMO into the non-noble metal. It is evident that in the area where the tunnel oxide has already been formed, the field drop across the PCMO is much lower as the voltage is divided between the high resistive oxide formed and the PCMO. Therefore, the growth rate of the tunnel oxide is increased in areas with less tunnel oxide if the resistance of the not fully formed tunnel oxide *R*_TO_ is lower than the resistance of the PCMO (*R*_TO_ < *R*_PCMO_). Thus, the inhomogeneity of the electric field causes an oxygen movement, which equalizes the inhomogeneity in the electric field.

The field homogenizing movement of oxygen during switching cannot be explained by this simplified simulation because the resistance of the tunnel oxide dominates the voltage divider *R*_TO_ ≫ *R*_PCMO_ (Figure 28d). Therefore, a more detailed geometry of the interface region has to be studied. A successful model must include the nonlinear field dependence of the resistance in the PCMO and in the tunnel oxide, the nonlinear dependence of the oxygen drift velocity, and the spatial variation of oxygen within both PCMO and the TO.

The above considerations describe inhomogeneity-compensating fluxes through fixed static interfaces of different materials. Also, the movement of oxygen vacancies within one material might have an influence on homogeneity. A generalizing approach considering the curvatures of interfaces of phase growth within the same matrix material has been described by Dittmann et al.^4^ There, the phase growth is generalized to an arbitrary kind of phase growing through a generalized flux caused by a generalized thermodynamic potential. In order to obtain a roughness-reducing flux, the thermodynamic potential that drives the flux in the source phase has to be of the nature that its gradient decreases in the region of the convex phase boundary and increases in the region of the concave phase boundary (Figure 29c).

**Figure 29:**
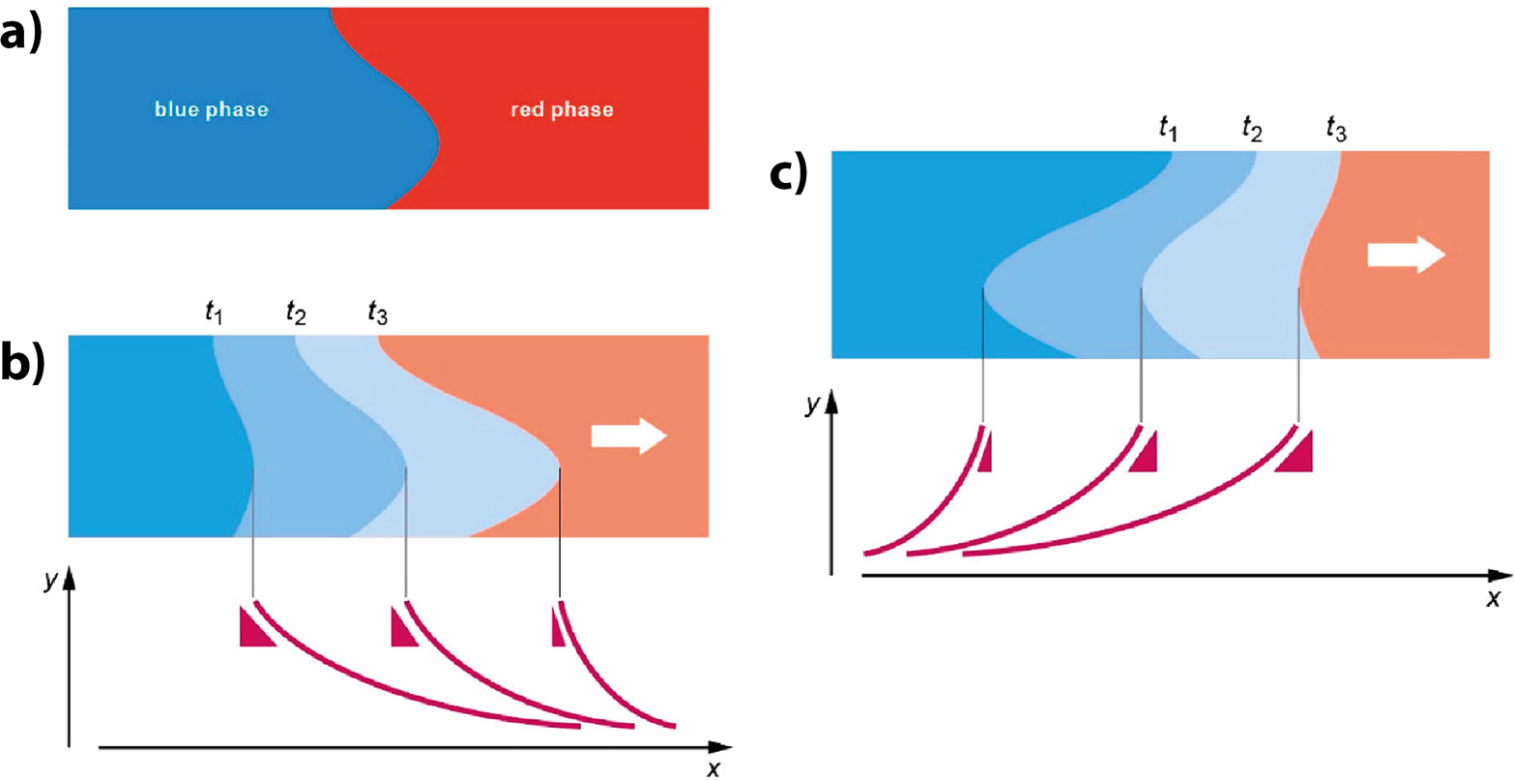
Curvature increasing flux b) and reducing flux c) fluxes of phase growth in a matrix as depicted in a). Adapted and reprinted with permission from ref 4. Copyright 2022 by Taylor & Francis.

### 4.2 Switching Mechanism Overview

The switching mechanisms discussed in the following sections are based on the valence change mechanism (VCM),^4^ which is caused by the movement of oxygen vacancies from the PCMO into the tunnel oxide during the SET and vice versa for the RESET, as depicted in Figure 30.

**Figure 30:**
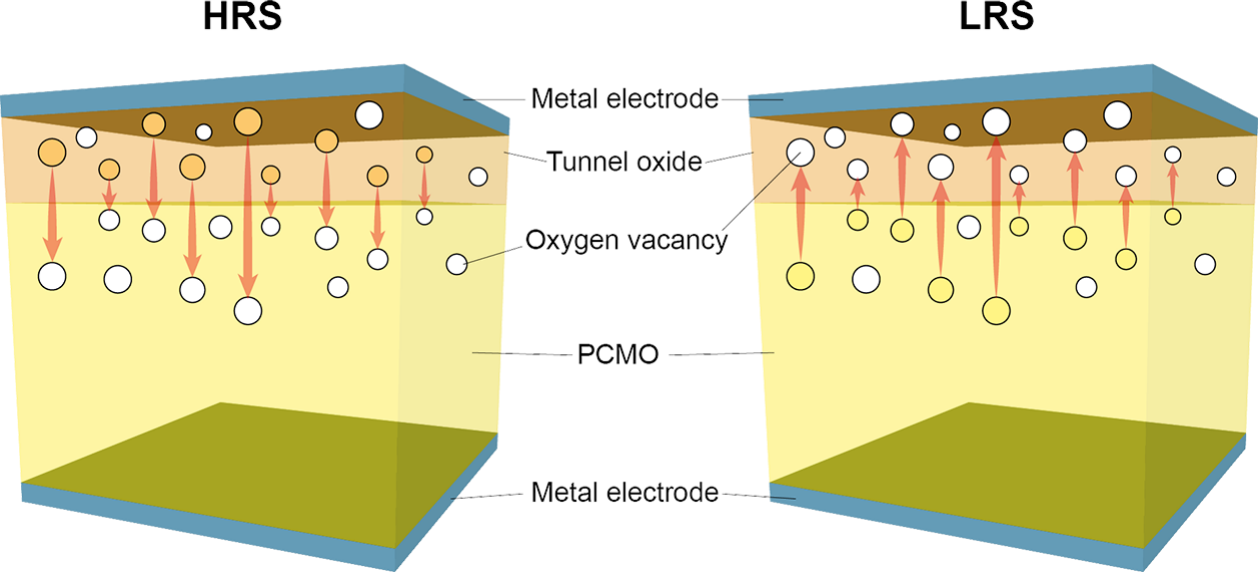
VCM mechanism - field induced movement of oxygen vacancies, between PCMO and TO. The figures show the stack in two different resistive states, left HRS and right LRS. The arrows show the movement which the oxygen vacancies did, to reach the resistive state.. The filling of the white cycles symbolizes the refilling of the vacancy with oxygen.

Several mechanisms have been proposed in the literature to explain the change in current transport with vacancy migration. The mechanism can be roughly divided into effects that the oxygen vacancies can have on the tunnel oxide and effects on the PCMO. Figure 31 gives an overview of the possible changes in the tunnel oxide, comprising a) the variation of thickness (section 4.3.1), b) the variation in trap states (section 4.3.1), c) the variation of space charge in the tunnel oxide (section 4.3.2) and the d) variation of the band gap (section 4.3.3). The variation of the dielectric constant (section 4.3.4) can also be considered as a potential model, leading to a change in the band bending.

**Figure 31:**
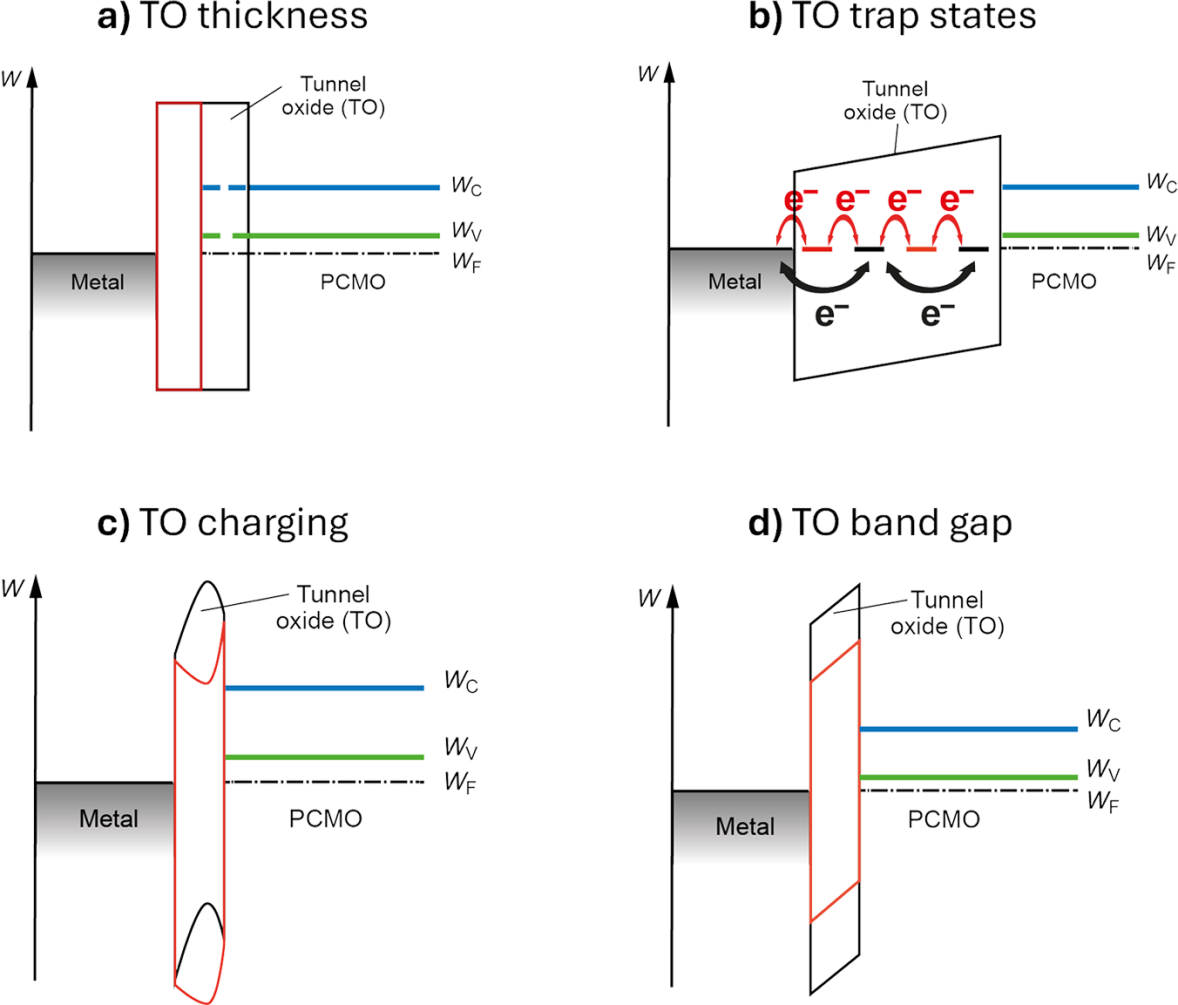
(a–d) Schematic overview of switching mechanisms based on the variation of the tunnel oxide barrier by oxygen vacancies. The LRS is depicted in red and the HRS in black.

In addition Figure 32 gives an overview of the switching mechanism in the PCMO-TO devices. These are a) the reduction in electronic mobility (section 4.4.1), b) the variation in carrier density and hence the broadening of the space charge region (section 4.4.2), c) the change in the work function (section 4.4.3) and d) the modification of the band gap (section 4.4.3). All of these mechanisms have been proposed in the literature and are discussed in the corresponding sections.

**Figure 32:**
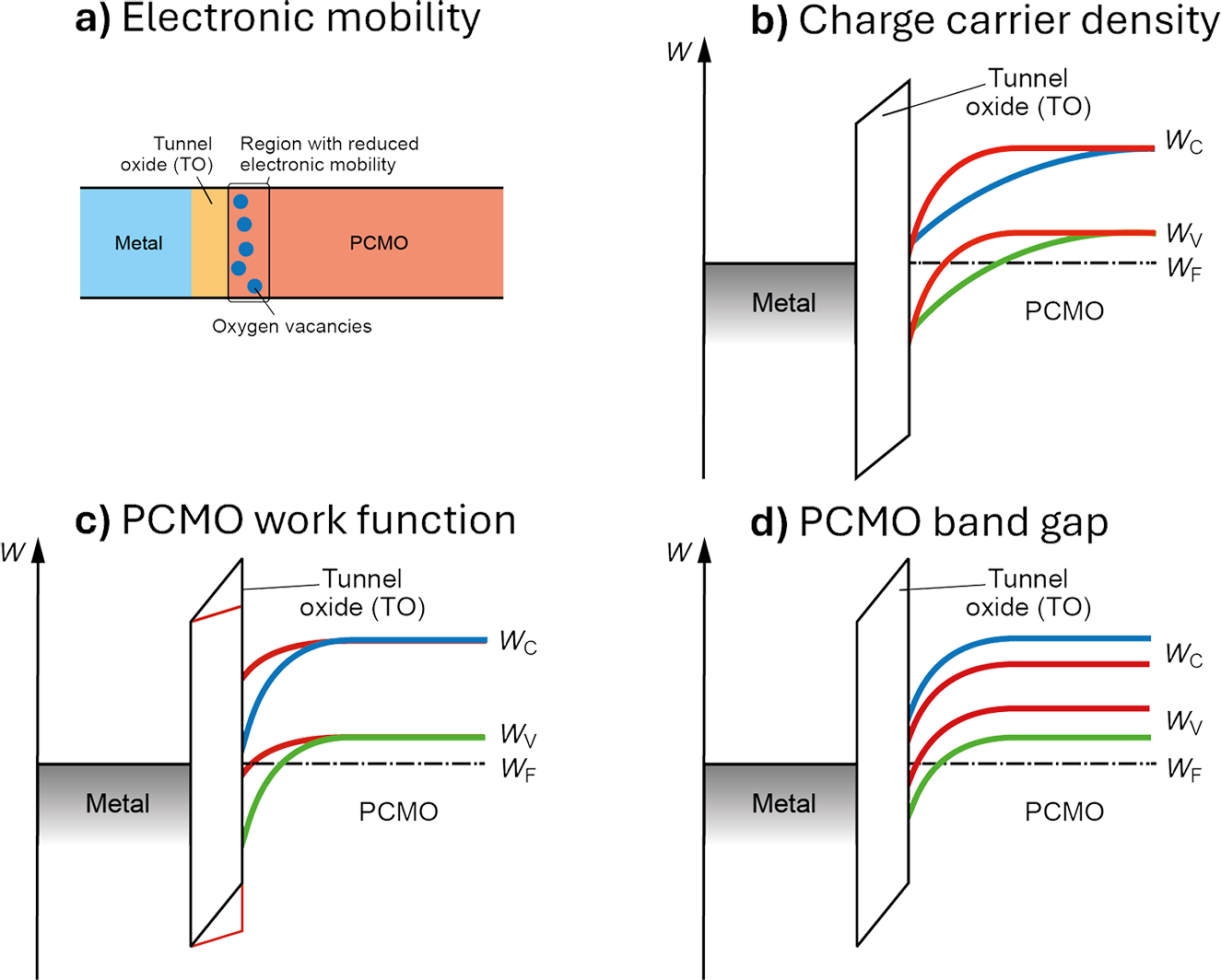
(a–d) Schematic overview of switching mechanisms based on the variation of the PCMO by oxygen vacancies. The LRS is depicted in red and the HRS in black.

### 4.3 Switching Mechanism Based on Changes of the Tunnel Oxide

#### 4.3.1 Resistance Change by Modifications of Trap Density and Barrier Thickness

As discussed in section 3.4.2, the introduction of defect states in the TO can facilitate the charge transport by trap assisted tunneling and thus reduce the tunneling current. This mechanism depends highly on the location of the defect states in the band gap of the tunnel oxide and thus depends on the tunnel oxide material.^76,86^ Gutsche et al.^60^ included this mechanism in their switching model for Al/PCMO-based devices.

Furthermore, as discussed in section 3.4, the tunnel current is exponentially dependent on the tunnel oxide thickness. Thus, reducing the TO thickness by oxygen extraction during the set reduces the resistance. Baek et al.^66^ showed by in situ TEM observation that the growing and shrinking of an a-TiO_*x*_N_*y*_ interlayer at the TiN/PCMO interface controls the area-dependent switching (Figure 33). Herpers et al.^54^ also proposed the decrease of the interfacial oxide TiO_*x*_ oxide in PCMO/Ti devices as a switching mechanism (discussed in section 3.4.1). Asanuma et al.^32^ observed an interfacial TiO_*x*_ oxide layer for PrMnO_3_/Ti, Pr_0.5_Ca_0.5_MnO_3_/Ti, and CaMnO_3_/Ti devices after electronic biasing, however only the PrMnO_3_/Ti and Pr_0.5_Ca_0.5_MnO_3_/Ti exhibit hysteretic switching. Further, Arndt et al.^74,87^ found for PCMO/YSZ/Pt resistive switching without change of the oxide thickness. Therefore, the modulation of the TO thickness is not suitable to explain the switching in all kinds of PCMO-based stacks.

**Figure 33:**
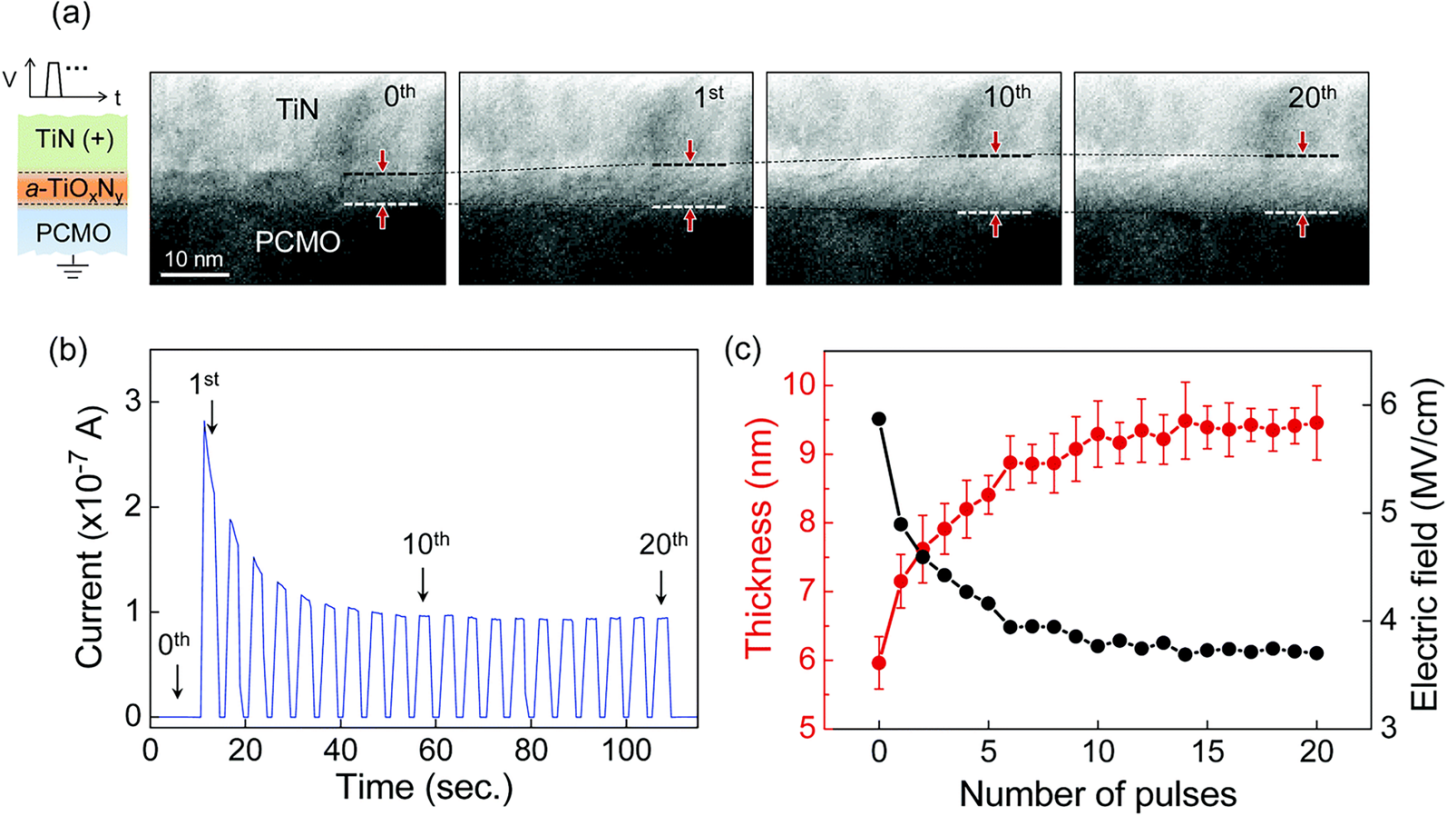
In-situ observation of a-TiO_*x*_N_*y*_ thickness variation with resistive states. Adapted and reprinted with permission from^66^. Copyright 2017 by American Chemical Society.

#### 4.3.2 Charging of the Tunnel Oxide

There are ways in which the change in the oxygen content can alter the tunnel transport across the TO. The oxygen anion O^2–^ are considered as a mobile negative space charge, which bends both the valence and conduction band of the TO upward and thus increases the effective height of the tunnel barrier (Figure 34). This model was used by Rene Meyer et al. to explain the switching behavior of TO/PCMO-based devices.^4,5,73^

**Figure 34:**
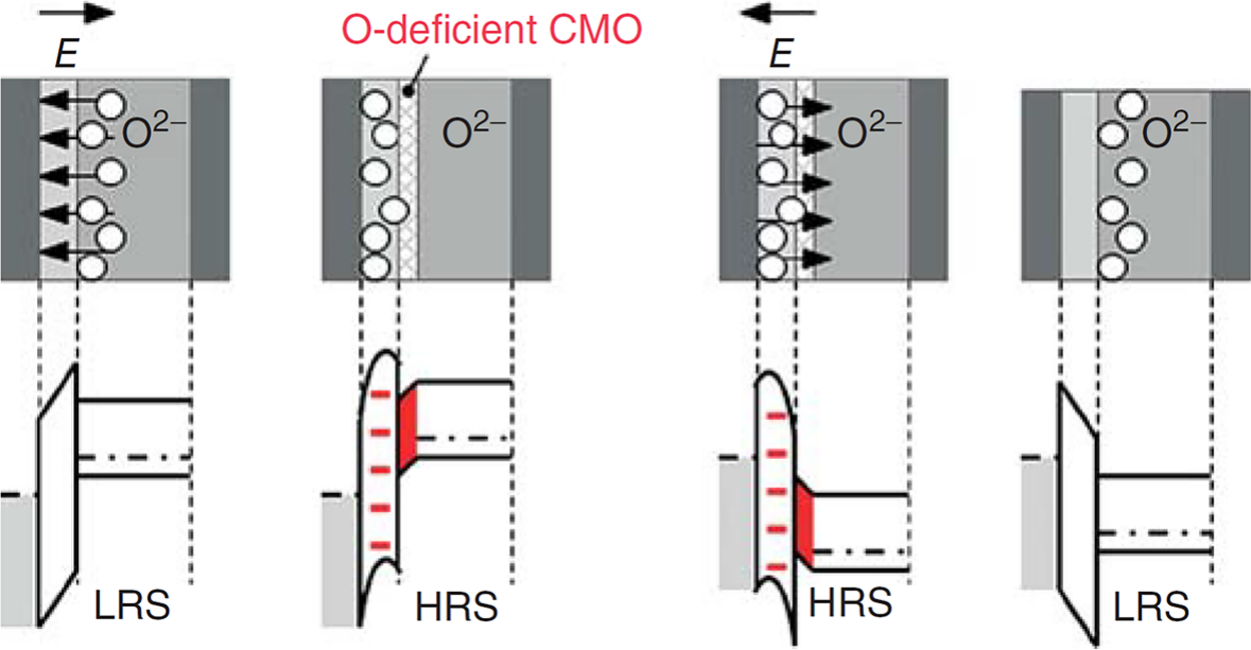
Switching mechanism based on charging of the tunnel oxide. Adapted and reprinted with permission.^5^ Copyright 2016 by John Wiley and Sons.

Arndt et al.^87^ experimentally showed that the electrostatic modulation of the Y-stabilized ZrO_2_ (YSZ) tunnel barrier in the Rh/YSZ/PCMO structure does not explain the resistive switching in this structure as proposed by Meyer et al.^5,73^ Arndt et al.^74^ compared the peak level shift of the Rh 3d and Zr 3d core levels by hard X-ray photoelectron spectroscopy during the resistive switching cycle (Figure 35b). They observed a remanent shift of around 0.34 eV of the Zr 3d peak in the LRS to lower binding energy. Since the Rh 3d level shows no shift, Arndt et al.^74^ assigned the binding energy reduction to a change in the YSZ. The reduction of the binding energy in the LRS would be an experimental indication of an increase in negative space charge in YSZ. However, this contradicts the space charge model, as shown in (Figure 35), since a negative space charge would bend the tunnel barrier upward and lead to the HRS. Arndt assigned the reduction of the binding energy to a decrease of the YSZ band gap in the LRS and introduced a new switching mechanism, as will be discussed in the next section.^87^

**Figure 35:**
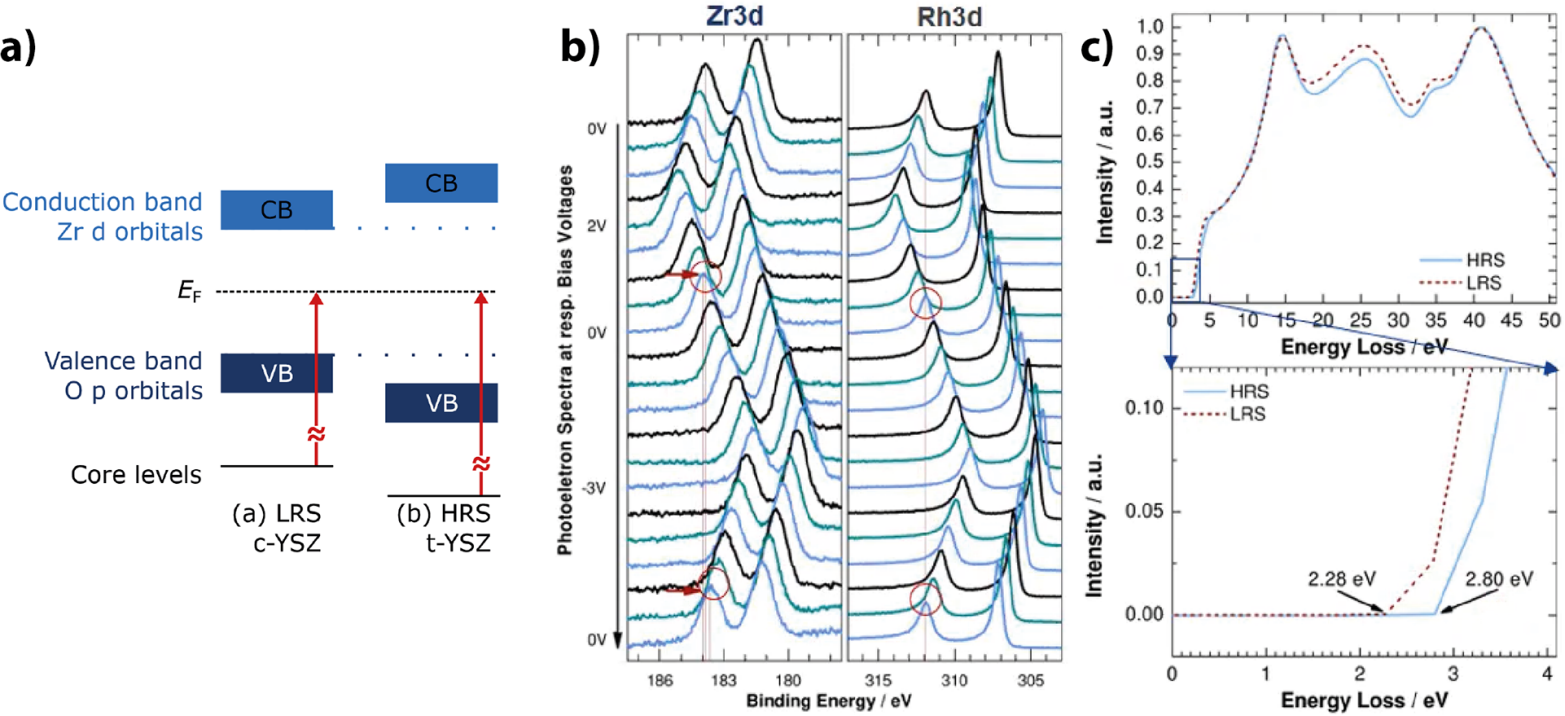
a) Correlation of band gap change and core level change. The original reference is ref 88. b) Change of the Zr 3d and Rh 3d core level in in situ HAXPES with the variation of the applied voltage. c) Variation of the EELS-spectra of YSZ in HRS and LRS. All figures are taken from ref 87.

#### 4.3.3 Change of the Band Gap of the Tunnel Oxide

The height of the tunnel barrier for direct tunneling is given on each site of the tunnel barrier by the difference between the work function of the metal or PCMO and the electron affinity of the TO. A decrease of the band gap of the TO caused by oxygen vacancies would increase the electron affinity of the tunnel oxide and thus reduce the tunnel barrier, as depicted in Figure 31.

Arndt^87^ showed by XPS and EELS analysis a decrease of the YSZ band gap in the LRS compared to the HRS which will be explained in the following. XPS determines the binding energy in reference to the Fermi level. Assuming a constant position of the Fermi level in the middle of the band gap, a reduction of the band gap would be experimentally visible by a reduction of the binding energy, as can be seen in Figure 35a. This was observed by Arndt, for the binding energy of Zr 3d_5/2_, which is lower in the LRS (181.24 eV) than in the HRS (181.58 eV). The band gap reduction is further supported by EELS data of the YSZ in LRS and HRS. This can be seen from the onset of the signal of electron energy loss in reference to the direct beam, which directly measures the band gap (Figure 35c). Arndt measured the onset of the energy loss signal at 2.28 eV for LRS and at 2.80 eV for the HRS.

Arndt assigned the band gap change of the local bonding configuration of the amorphous YSZ from a cubic configuration in the oxygen vacancy-rich LRS to a tetragonal configuration in the HRS. The fact that a cubic phase can be stabilized by oxygen vacancies was reported by Fabris et al.^89^ The relationship between band gap and phase was found by Jiang et al.^90^ and Götsch et al.^91^ A change from cubic to the tetragonal phase relates to an increase of the shoulder around 530 eV in the Oxygen K edge, as shown by McComb^92^ and Ostanin et al.^93^ This change is represented in the EELS data of Arndt (Figure 36). However, Arndt deposited YSZ with PLD at room temperature. Thus, the YSZ is amorphous and does not have a crystal symmetry, which could be assigned to name a phase cubic or tetragonal. The material change here can be seen as a change in the near order.

**Figure 36:**
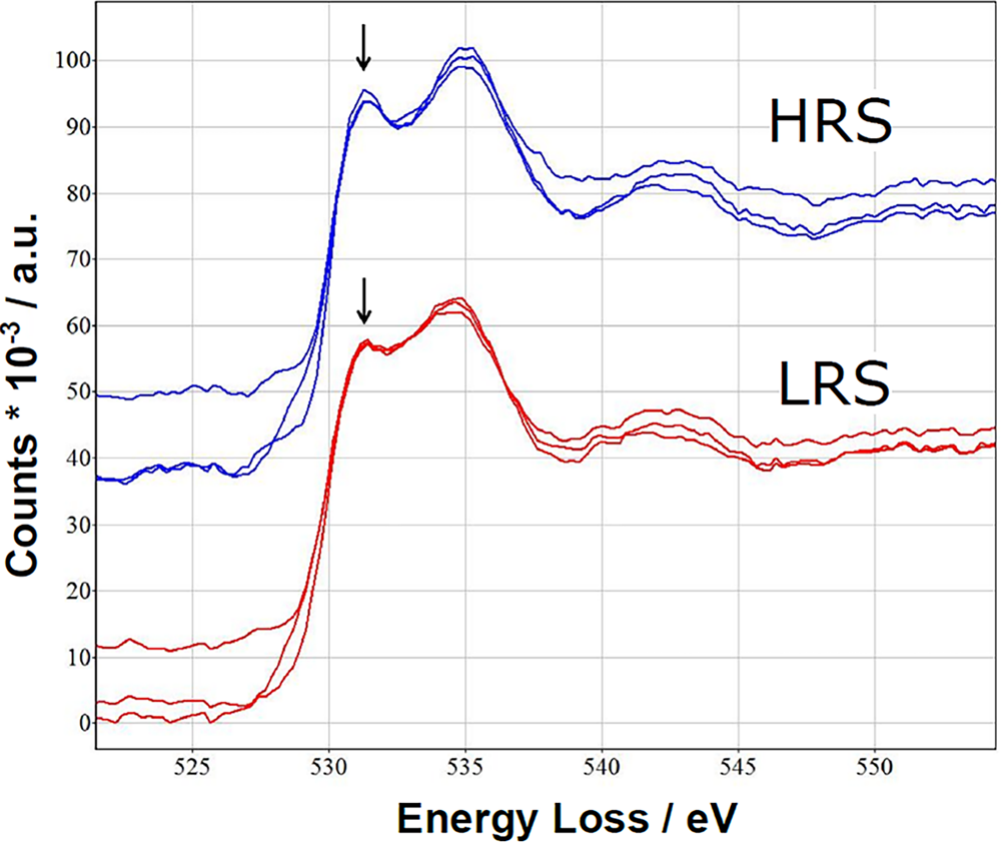
Difference of the oxygen K-edge EELS data of amorphous YSZ in a metal/YSZ/PCMO/SRO stack in HRS and LRS. Ni had been used as the top electrode. All figures are taken from ref 87.

#### 4.3.4 Influence of Permittivity on the Band Bending and Tunneling

As already discussed in section 2.7, 3.3, and 4.4.2 the higher the permittivity, the more space charge is screened and thus the lower is the effect on the band bending. As the MOP stack contains two dielectric materials, the effect of the space charge depends on the relative permittivity between the PCMO and the tunnel oxide.

This has been considered by Sommer et al.^94^ for a metal (high work function)/tunnel oxide/conducting oxide (low electron affinity – n-type)/metal (low work function) stack. In that case, the work function difference between the metal and the conducting oxide gives a negative built-in voltage and forms a Schottky barrier. The contact between the conducting oxide and the low work function metal is ohmic. The stack is shown in Figure 38a, and the two different oxygen vacancies profiles are plotted in red and blue in Figure 38b. The simulated stack deviates from the PCMO system due to several reasons. First, the intrinsic charge carrier concentration of PCMO is larger than 10^21^/cm^3^ (see section 3.3) and therefore much higher than the assumed 10^18^/cm^3^ in the simulation. Therefore, the effect of oxygen vacancy doping on the change of the carrier concentration is much less pronounced than in PCMO. Second, PCMO can be both n or p-type, as discussed in section 2.3.

The three possible scenarios addressed in reference^94^ and shown in Figure 38 are a) permittivity higher in the conduction oxide, b) equal permittivity, and c) higher permittivity in the tunnel oxide (Figure 38c). The red line indicates the scenario with high vacancy concentration in the tunnel oxide, as parametrized in Figure 37b. In the area of higher doping concentration, the conduction band is bent downward because of the higher remaining space charge related to the doping. The lower the permittivity is in the TO, the less the remaining space charge is screened and the stronger its effect on the band bending. Sommer et al. found that the hysteresis in the *I–V* curves (Figure 38a) is caused by the difference in band bending of the tunnel oxide if the relative permittivity is low and thus the band bending is pronounced. In the alternative scenario Figure 38c, a change in the switching polarity is observed, which is caused by an increase of electrons at the interface in the conducting oxide and thus an effective decrease of the tunneling distance for a positive applied bias and vice versa for an applied negative bias.

**Figure 37:**
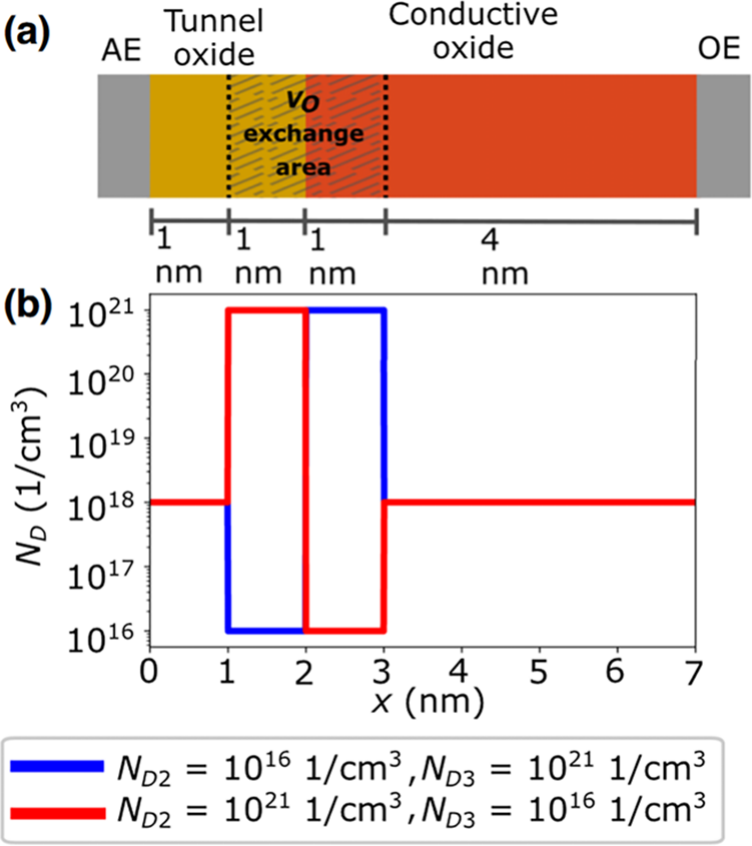
a) shows the geometry of the simulated 1-dimensional model and b) shows the static donor concentration caused by oxygen vacancies. Taken from ref 94.

**Figure 38:**
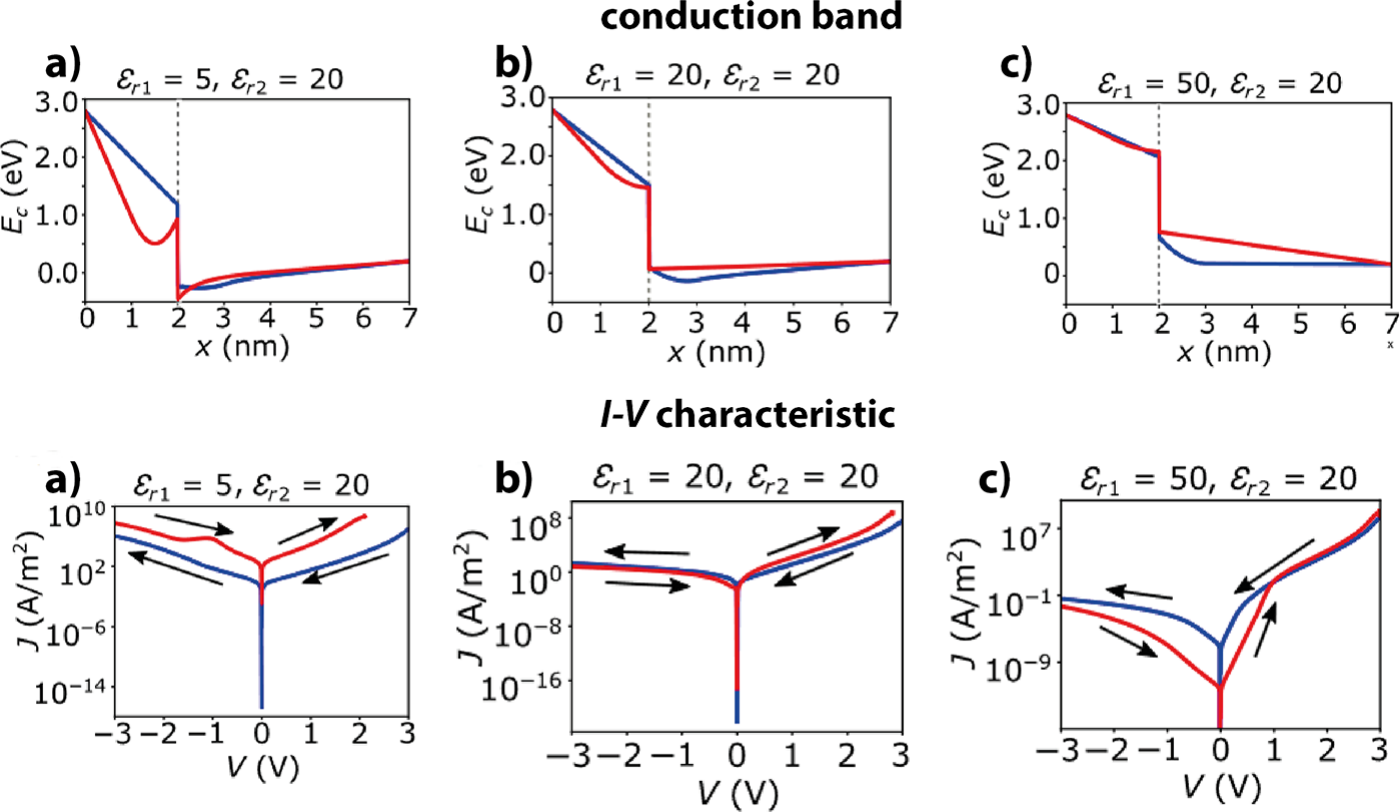
Figures show the conduction band edge and the resulting *I*–*V* loops for the two different oxygen vacancy distributions marked by red and blue, which corresponds to Figure 37b. Simulated are the possible scenarios with relative permittivity differences: a) permittivity higher in the conduction oxide, b) equal permittivity, and c) higher permittivity in the tunnel oxide. Taken from ref 94.

### 4.4 Switching Mechanisms Based on the Modification of PCMO

#### 4.4.1 Electron Mobility and Trap Density Changes

As discussed in section 2.5 and illustrated in Figure 31, oxygen vacancies increase the resistance of PCMO and can therefore contribute to resistive switching. In particular, the increase is associated with a decrease in electrical mobility. It also allows the electric field to stray from the TO into the PCMO and facilitates the movement of the vacancies. The increase in resistance has also been referred to as a Mott transition by Lee et al.,^30^ a term also discussed in section 2.5.

Systems in which PCMO is sandwiched between two noble electrodes can, due to the absence of a tunnel oxide, be used to investigate the effect of oxygen vacancies in the PCMO layer. Several investigations of PCMO-devices with two noble electrodes assign the effect to the interface. In particular, Scherf et al.^95^ investigated PCMO with Pt, Au, and Ag electrodes in different geometrical arrangements and located the switching process to the interfaces, which are competing in their dominance on the resistive change. Most importantly, Kramer et al.^96^ proofed by in situ environmental TEM investigation that the switching in Pt/PCMO/Pt lamellae is connected to the electromigration of oxygen.

Although PCMO does not meet the requirements of SCLC (section 3.5), some studies describe switching in PCMO-based devices by a variation of SCLC caused by changing the trap density in the material. For example, Chakraborty et al.^79^ claimed for W/PCMO that the resistive state changes from LRS and HRS by increasing the trap density in the PCMO. This model was further developed by Saraswat et al.^97^ into a quantitative reaction-drift model that includes ionic transport and describes PCMO switching based on variation of bulk defect states.

#### 4.4.2 Influence of Depletion Zone Variation on Tunneling

The influence of oxygen vacancies on the tunnel current does not only depend on the change of the tunnel barrier height/bending or defect level concentration in the tunnel oxide as shown in Figure 32. The change in PCMO caused by oxygen vacancies can also affect the tunnel current. As shown in the simulation of the previous section, increasing the charge carrier density in the interfacial region can also increase the tunnel current. Charging the depth of the depletion region could also change the width of the tunnel barrier and, thus, the transport.

As discussed in section 3.3, although the space charge region can be detected by photoelectron spectroscopy, it is too narrow to cause a rectification in the *I–V* curves of the metal/PCMO Schottky junction. However, a strong reduction of the charge carrier concentration can reduce the depletion zone (as can be seen in Figure 19) and thus increase the tunnel current. As discussed in section 2.5, switching can increase the oxygen vacancies and deplete the charge carrier density. For Ti/PCMO devices, Sawa^98^ found that the capacity is increased in the LRS compared to the HRS (Figure 39) and concluded that the depletion zone is thinner in the LRS than in the HRS. As discussed in section 2.7, also the permittivity of the oxygen-vacancy rich PCMO region could increase. This would further increase the depletion zone (Figure 19) and support this switching mechanism. However, a shortcoming of this model^33,98^ is that they did not include the TiO_*x*_ tunnel oxide at the interface.^32^ Furthermore, the charge carrier due to oxygen vacancies causes the opposite effect for p-type and n-type PCMO. Therefore, the model predicts opposite switching directions. This cannot be seen when comparing devices based on Pr_0.5_Ca_0.5_MnO_3_ (n-type) and devices based on Pr_0.7_Ca_0.3_MnO_3_ (p-type), as both switches in counter-eightwise direction.^33,54,72,74,98^ The switching direction is independent of the charge carrier type of PCMO if the change of the permittivity is considered solely.

**Figure 39:**
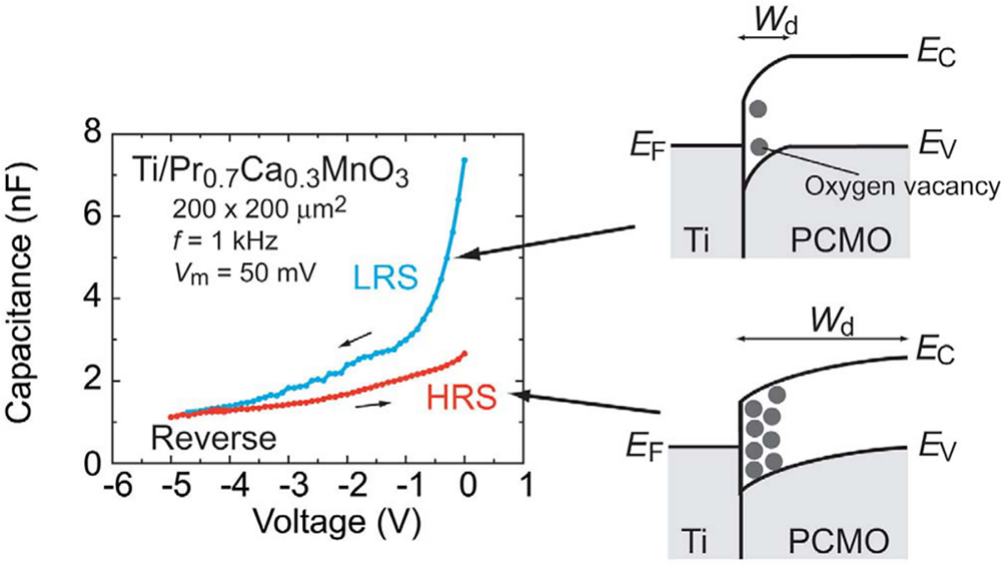
Switching explanation for the PCMO/Ti structure based on the modulation of the space charge region by oxygen vacancies. Adapted and reprinted with permission from ref 98. Copyright 2008 by Elsevier.

#### 4.4.3 Change of Band Gap and Work Function

Besides the charge carrier density and the permittivity also, the work function and the band gap of PCMO can change by oxygen redistribution. As discussed in section 2.6, the work function of PCMO can decrease after thermal reduction about 0.1 eV for PrMnO_3_, 0.1 eV (Pr_0.5_Ca_0.5_MnO_3_) and 0.2 eV (CaMnO_3_). As discussed in section 2.5, the bad gap of PCMO can also increase after thermal reduction around 0.4 eV for PrMnO_3_, 0.4 eV for CaMnO_3_, and 0.2 eV for Pr_0.5_Ca_0.5_MnO_3_. As can be seen in Figure 40a, both effects add up and lift the bottom edge of the conduction band in the reduced PCMO. These effects counteract the shift of the valence band. If the change of the band gap dominates, the valence band edge effectively shifts down.

**Figure 40:**
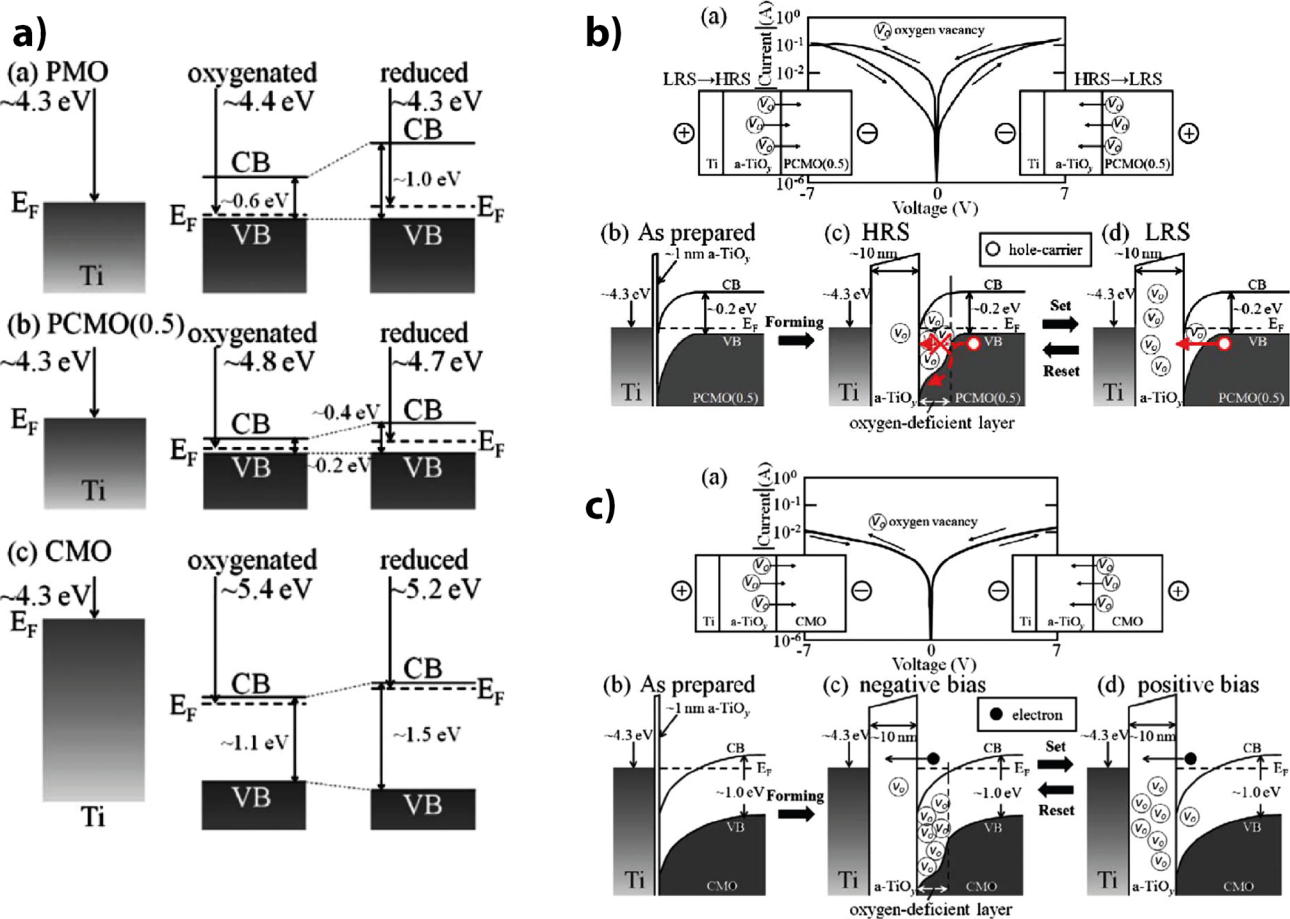
Band diagram-based explanation for resistive switching of the Ti/TiO_x_/PCMO structures. a) Shows the band structure of PrMnO_3_ Pr_0.5_Ca_0.5_MnO_3_ and CaMnO_3_ oxidized and reduced in relation to Ti. b) Shows the resistive switching cycle of Ti/TiO_*x*_/Pr_0.5_Ca_0.5_MnO_3_ and the proposed band diagram change. c) It shows the missing resistive switching of *I*–*V* cycles in Ti/TiO_*x*_/CaMnO_3_ structures and provides a band structure based explanation. Adapted and reprinted with permission from ref 32. Copyright 2009 by American Physical Society.

Asanuma et al.^32^ used the band diagram-based description shown in Figure 40a to explain the resistive switching. They found that Ti/TiO_*x*_ devices based on PrMnO_3_ and Pr_0.5_Ca_0.5_MnO_3_ exhibit resistive switching and that devices based on CaMnO_3_ do not switch. For the explanation, they assume that PrMnO_3_ and Pr_0.5_Ca_0.5_MnO_3_ are p-conducting and that CaMnO_3_ is n-conducting. Therefore, Asanuma et al.^32^ concluded from the band diagram that the change of the band bending by the oxygen redistribution only influences the transport within the valence band. Since the transport for n-type CaMnO_3_ occurs within the conduction band and the work function is such that the bottom of the conduction band is below the Fermi level of the Ti, transport is unaffected by the change in band gap and work function (Figure 40b). For the transport within the valence band as relevant for p-type PrMnO_3_ and Pr_0.5_Ca_0.5_MnO_3_, the increase in band gap overcompensates the decrease in work function in the oxygen-deficient PCMO interface. As a result, the band bending increases, and the broadening of the tunnelling barrier causes an HRS (Figure 40c). However, their assumption of p-type Pr_0.5_Ca_0.5_MnO_3_ is not covered by the Seebeck coefficient, as discussed in section 2.3. Further restricting the switching model on variations in PCMO ignores the effect that oxygen vacancies have on the tunnel oxide. We must conclude that the relevant switching mechanism depends on the material selection in the MOP stacks. Further, different mechanisms can work in parallel; thus one does not necessarily have to dominate.

### 4.5 Switching Kinetics

The switching kinetics of PCMO heterostructures is determined by the speed-limiting step for the field-accelerated movement of oxygen. It could either be limited by the drift within the tunnel oxide, within the PCMO, or across the PCMO/TO interface region. Furthermore, electron transfer can be rate-limiting for redox reactions at the interfaces.^99^ The electrical field can be modulated as a result of changes in resistance, permittivity, and space charge caused by the oxygen exchange. An important difference between the kinetic of area-dependent and filamentary devices is that area-dependent devices are generally considered to be less dominated by Joule heating and the connected thermal acceleration during switching.^4^

Kinetic considerations are important as a prerequisite for enabling resistive switching. Only if the electric field is sufficiently high (*E* > *E*_c_) will oxygen movement be facilitated and resistive switching be induced. The critical field *E*_c_ required to facilitate the movement depends on the movement mechanism, as discussed in section 4.5.1, and can be of the order of ∼1 MV/cm. The resistance of the materials and the resulting field drop within the stack is therefore important to the switching performance. As the switching takes place at the interface of PCMO/TO, it is particularly important that most of the field drops in this area. This is the case because the resistance of the tunnel oxide is usually higher than that of the PCMO. Further, in the interfacial region where the PCMO is reduced, the field can stray further into the PCMO and the space charge region could contribute. If the resistance of the PCMO becomes as high or higher than the tunnel oxide, this could lead to a field distribution whose main contribution is not within the active switching interface. This could lead to the kinetics prohibiting the ionic movement and could explain the poor or missing area-dependent switching of devices based on amorphous PCMO,^100–107^ which has a much higher resistance than crystalline PCMO.

During set and reset, the vacancies must cross the interface in the opposite direction. Since the reset leads to further oxidation of the TO and the set to an oxygen extraction, the critical voltage to overcome the energy barrier may be different for set and reset. Lee et al.^108^ showed that necessary voltage to do a set operation increases with the free energy of oxide formation of the TO oxide.

#### 4.5.1 Diffusion and Drift of Oxygen

As described in section 2.5 in detail, due to the densely packed perovskite lattice of PCMO, Frenkel defects can be excluded, and oxygen movement takes place via vacancies. Therefore, the vacancy can be described as the moving entity while using the same activation barrier Δ*W* as for the oxygen movement. The benefit of using the vacancy as a moving entity is that the vacancy usually has a neighboring oxygen anion since the number of vacancies *N*_V_ is usually much smaller than the number of oxygen anions *N*_O_, and therefore, the average velocity *v*_D_ (*V*_O*¨*_ = *N*_O_/*N*_V_ *v(O_O_) during self-diffusion is not reduced due to the limited possibility of neighboring exchange partners.^4^

The average drift velocity

4.1 $$v_{D}\left(\overset{¨}{V_{O}}\right)=a\omega e^{-\Delta W/k_{B}T}$$

during self-diffusion can be determined by the Arrhenius law $\propto e^{-\Delta W/k_{B}T}$ , the jumping distance *a* (usually interatomic distances of 0.2–0.5 nm^99^), and the attempt frequency ω. The attempt frequency is sometimes approximated by half of the Deby frequency,^109^ which would be 2 × 10^13^ Hz for Pr_0.8_Ca_0.2_MnO_3_ with a Debye Temperature of ∼ 325 K.^110^ The activation barrier Δ*W* can experimentally be determined from self-diffusion profiles by oxygen tracer experiments. Fitting the time-dependent diffusion profiles results in the diffusion coefficient $D\left(O_{O}\right)=\frac{av\left(O_{O}\right)}{2}$ , and the activation barrier Δ*W* can be determined from the temperature dependence.

The application of an electric field *E* leads to the directionality of the drift velocity *v*_D_ (*V̈*_O_) because of the reduced activation barrier by Δ*W* – *zeaE*/2, as sketched in Figure 41a. For the calculation of the effective drift velocity *v*_D_(*V̈*_O_), the vacancy hopping in the opposite direction must be subtracted, resulting in the so-called Mott–Gurney law^111^ of ion hopping

4.2 $$v_{D}\left(\overset{¨}{V_{O}}\right)=a\omega e^{-\Delta W/k_{B}T}2\;\sinh\left(zeaE/\left(2k_{B}T\right)\right)$$

which is plotted in Figure 41b. It is often written in the form of ionic current density:

4.3 $$J\left({\mathrm{V̈}}_{O}\right)=zecv_{D}\left({\mathrm{V̈}}_{O}\right)$$

where *c* is the vacancy concentration and *z* the valence number.

**Figure 41:**
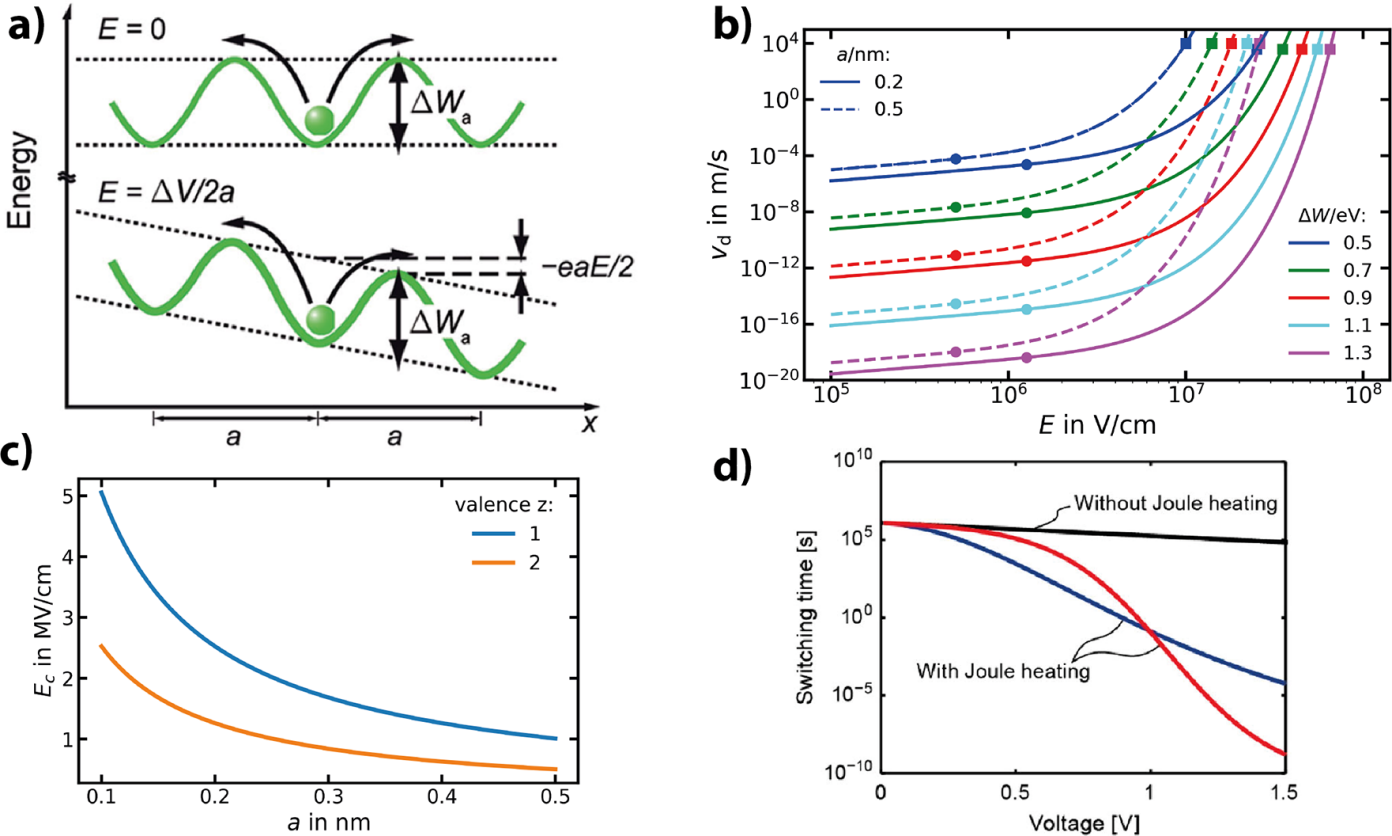
a) Geometric argument for the activation barrier reduction for field enhanced diffusion, leading to the Mott–Gurney law. Adapted and reprinted with permission from ref 99. Copyright 2015 by John Wiley and Sons. b) Drift velocity in dependence of the field according to eq 4.7 for different activation barriers Δ*W* and different jumping distances *a* at room temperatures for *z* = 2 and an attempt frequency of ω = 2 * 10^13^ Hz. *E*_c_ is marked by a dot and *E*_max_ is marked by a square. c) critical field (eq. 4.5) in dependence of valence and jumping distance. d) The influence of Joule heating on the switching was time calculated from Mott–Gurney based oxygen transport. The blue curve is for a linear current voltage relationship and the red for a diode like behavior. Adapted and reprinted with permission from ref 4. Copyright 2022 by Taylor & Francis.

The Mott–Gurney law is only valid until the field *E*_max_, shown by squares in Figure 41b. At this field strength, the electronic potential completely compensates the activation barrier Δ*W*

4.4 $$E_{\max}=\frac{2\Delta W}{zea}$$

The Mott–Gurney law converges at small fields in the linear regime of the sinh to the Einstein–Smoluchowski equation.^111^ The difference between the dashed and the solid lines in Figure 41b also shows that the drift velocity increases for larger jumping distances *a* if the activation barrier Δ*W* remains the same.

At high fields *E* > *E*_c_, with the critical field *E*_c_

4.5 $$E_{c}=2k_{B}T/\left(zea\right)$$

the field dependence of the ionic movement becomes exponential. *E*_c_ is dependent on the jumping distance and the valence number and is usually in the order of few MV/cm, as can be seen in Figure 41c. Controlling the switching kinetics by a nonlinear field dependence is important for the stability of the written state against decay over time (retention), for the stability against a reading voltage, and for fast writing speeds.^99^ This nonlinearity can be temperature accelerated by Joule heating^4^

4.6 $$T=T_{0}+R_{\mathrm{th}}I\left(V\right)V$$

by the electronic power *I* (*V*)*V*, especially when the current–voltage relationship is nonlinear. *R*_th_ is thermal resistance and considers the material parameters. The nonlinearity becomes particularly pronounced in cases of a nonlinear *I–V* relationship, as can be seen in the exemplary case of a diode with *I*(*V*) = *I*_0_ (exp­(*V*/*V*_0_)-1) in Figure 41d.

The ionic current density *J*(*V̈*_*O*_) or the drift velocity *v*_*D*_(*V̈*_*O*_) can relate to the switching time *t*_*SET*_ by

4.7 $$J\left({\mathrm{V̈}}_{O}\right)\propto t_{\mathrm{set}}^{-1}$$

To investigate the switching kinetics, the switching time *t*_SET_ must be clearly defined. Therefore, it is defined as the time which is needed to switch between two defined resistive states *R*_0_ (*c*_0_) and *R*_1_ (*c*_1_). The resistive states are given by two oxygen concentration profiles *c*_0_ and *c*_1_. The redistribution Δ*c* = *c*_1_ - *c*_0_ between both states is caused by the applied voltage. The same redistribution Δ*c* takes place only within the same time *t*_SET_ if the initial distribution *c*_0_ is the same. Therefore, controlling the initial resistance *R*_0_(*c*_0_) is crucial for a reliable analysis of the switching kinetics. If the initial resistance *R*_0_(*c*_0_) is experimentally well controlled, then a switching time can be defined as the time which is needed to achieve a certain arbitrary resistance ratio (*R*_1_(*c*_1_)/(*R*_0_(*c*_0_).

For the investigation of the switching kinetics, *t*_set_ is plotted against the voltage (Figure 42b). If the kinetically limiting process is ion migration according to the Mott–Gurney law, the field strength is larger than *E*_c_, and if Joule heating can be disregarded, than *t*_set_ should lie in the semilogarithmic plot on a straight line^4^ with a slope *m* of

4.8 $$m=-\frac{aze}{2k_{B}Tw}$$

where *w* is the device thickness over which the voltage drops.

**Figure 42:**
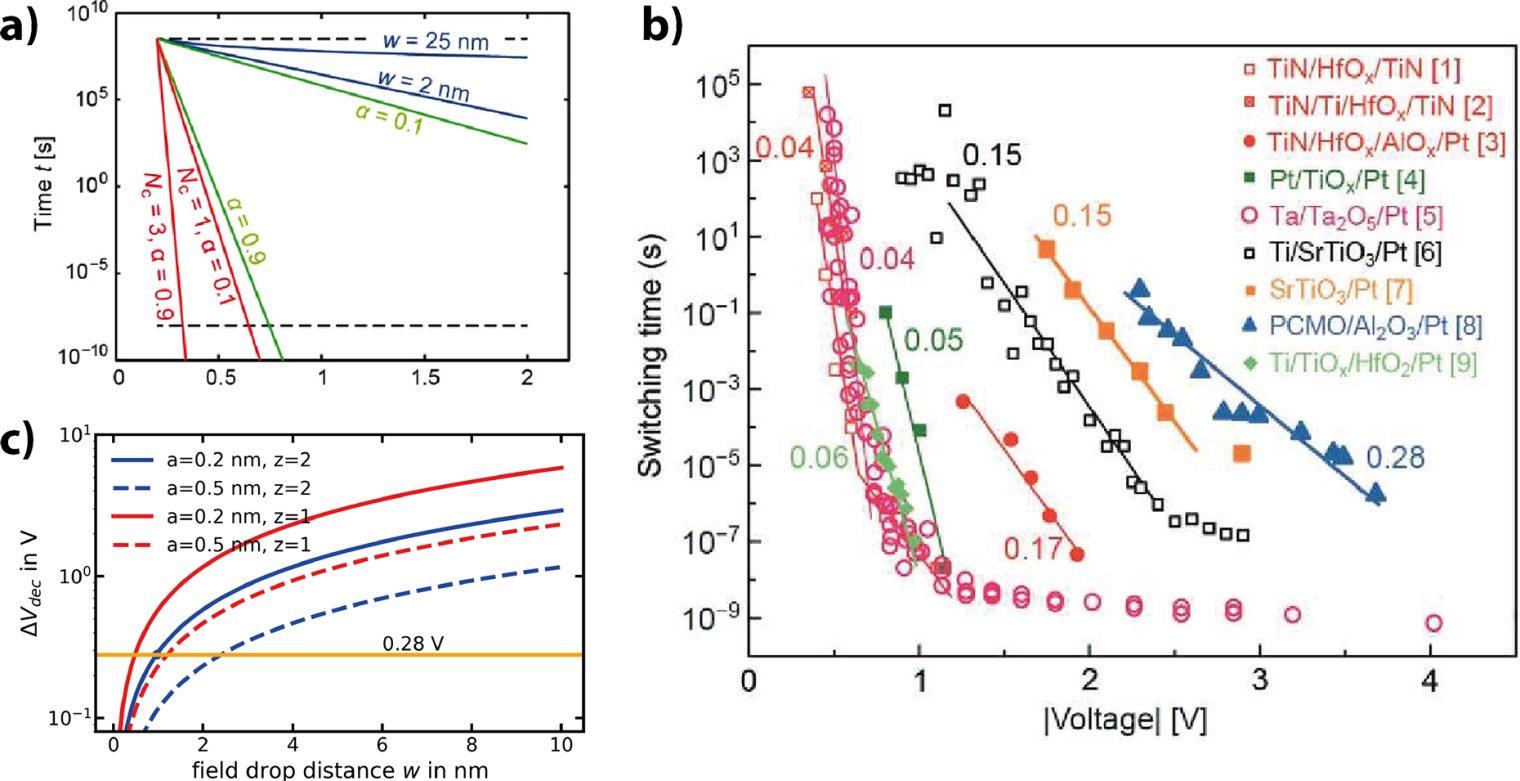
a) Comparing the field dependency of different kinetic limiting processes (blue - ionic hopping, green - nucleation, red - electron transfer) under a span of different physical parameters. Adapted and reprinted with permission from ref 4. Copyright 2022 by Taylor & Francis. b) Set time plotted against applied voltage for different VCM stacks. Adapted and reprinted with permission from ref 112. Copyright 2023 by IEEE. The written numbers are Δ*V*_dec_ of the system. c) Δ*V*_dec_ in dependence of the field drop distance *w* for different parameters in the only field accelerated Mott–Gurney law with *E* > *E*_c_. Δ*V*_dec_ = 0.28 V of the PCMO/Al_2_O_3_/Pt devices is shown by the orange line.

In addition to ion migration, other mechanisms could also limit the kinetics, such as, the electronic transfer at the interface, which can be described by the Buttler-Volmer equation. For electrochemical metallization cells, the formation of a stable metallic nucleus prior to the filament growth could also become kinetically limiting.^4,99^ However, both competing processes result in an exponential field dependence and would result in a line in the semilogarithmic plot. Menzel et al.^4,99^ compared the slopes under a span of physically reasonable parameters Figure 42a and concluded that the Mott–Gurney based ion migration has the lowest slope and thus is generally likely to be the dominating process at high voltages, as long as the Joule heating does not cause higher nonlinearity (Figure 41d) as is the case of filamentary VCM devices.

Since PCMO/Al_2_O_3_/Pt based devices lie on a fairly straight line^112^ at high voltages Figure 42b with a reasonable slope for the Mott–Gurney law, the kinetic of PCMO-based devices is likely controlled by ion migration without thermal acceleration. The slope *m* is given by^4^

4.9 $$\Delta V_{\mathrm{dec}}=-\frac{1}{m}\;\ln\left(10\right)$$

the voltage which is necessary to increase the switching time by 1 order of magnitude. Δ*V*_dec_ is plotted in Figure 42c for different reasonable parameters in the case of a pure field dependent Mott–Gurney governed dynamic. As smaller Δ*V*_dec_ is favorable. The smaller Δ*V*_dec_ is, the shorter is the needed pulse to write a resistance state at high voltage. Further, the smaller Δ*V*_dec_ is, the longer can the resistance state be read at a low voltage without disturbing the state. However, this argumentation is only valid if the whole voltage range is dominated by one kinetic limiting mechanism.

Figure 42c shows possible Δ*V*_*dec*_ in dependence of the field drop distance *w* for the sole field accelerated Mott–Gurney law at *E* *>* *E*_c_. It shows, on the one hand, that this mechanism is not able to achieve high nonlinearity for large time differences between reading and writing. It also shows that the nonlinearity can be improved by material stacks in which the voltages drop across very thin layers.

The Δ*V*_dec_ = 0.28*V* for PCMO/Al_2_O_3_/Pt devices (shown in Figure 42c by an orange line) leads to the conclusion that in the devices the voltage drops in the a region of around ∼1 nm (crossing lines with the different plotted Δ*V*_dec_). Thus, it could be concluded that the active switching region of field drop including the AlO_*x*_ ^112^ reduced PCMO and the space charge region are very narrow.

The lack of temperature acceleration in the kinetics of TaO_*x*_/PCMO devices is further indicated by the behavior of the potentiation and depression measurements (section 5.1.1) performed by Gutsche et al.^62^ Here the change in resistive state depends only on the total applied time of a voltage pulse. The application of many short voltage pulses results in the same resistance changes as a long voltage pulse. This indicates that a longer voltage pulse does not further heat the device and thus thermally increase the vacancy mobility. This analogue switching property of PCMO-based devices, and area-dependent devices in general, makes them suitable for neuromorphic applications (section 5).

However, there are also studies that have found the effect of Joule heating on the kinetics of PCMO-based devices. These studies are based on the W/PCMO system, which exhibits a sharp SET transition in its IV loop and requires current compliance for SET, as is typical for filamentary systems. Although the group claims area dependence,^78^ there is no clear experimental evidence for area scaling of the low resistance state. Therefore, Joule heating in these W/PCMO stacks might be caused by a local confinement of the switching process within the interface oxide. A similar sharp SET behavior has been found for Ti/PCMO devices and has been attributed there to multifilamentary switching within the interfacial oxide.^54^

The experimental evidence for self-heating was found in the SET and RESET transients of W/PCMO devices, with time scales for self-heating between 50 and 100 ns.^113^ This was further complemented by experimental studies of SET voltage versus SET time, which showed steep slopes and saturation at about 100 ns.^97^ These results are accompanied by computational models simulating device temperature.^97,113^ However, these models include controversial approaches with SCLC for electrical transport (section 3.5) and bulk trap variation as switching mechanisms (section 4.4.1).

The switching time is an important quality parameter of PCMO-based devices, determining the possible memory applications (section 5.3). While it is obvious that the switching times speed up with higher voltages (Figure 42), high voltages can lead to poor endurance and difficulties in CMOS integration. However, the switching speed limit is still unknown. While Saraswat et al.^97^ found a switching limit of 100 ns at around 2.5 V for their W/PCMO based devices, the commercial company 4DS Memory publicly claims a switching speed of 4.7 ns for their PCMO based memory arrays.

#### 4.5.2 Retention

Instabilities of the resistive states which limit the retention of memristive devices are induced by the redistributing diffusion of ions in a chemical gradient without applied voltage. The diffusion current can be considered as temperature accelerated by the activation energy Δ*W* as in eq 4.1, and the retention time *t*_ret_ can be assumed to be described by^4^

4.10 $$t_{\mathrm{ret}}≃Ae^{-\Delta W/\left(k_{B}T\right)}$$

where *A* is a constant. Therefore, high activation energies are beneficial for high retention times.^114^

Measurements of the temperature dependence of the retention times can, therefore, be used to determine the activation energy, which are called accelerated life testing. Kumbhare and Ganguly^115^ analyzed the retention time of PCMO/W devices in dependence on the PCMO stoichiometry. They determined Δ*W* of Δ*W*(PrMnO_3_) = 1.29 eV, Δ*W*Pr_0.9_Ca_0.1_MnO_3_) = 1.12 eV, Δ*W*(Pr_0.8_Ca_0.2_MnO_3_) = 1.26 eV and, Δ*W*(CaMnO_3_) = 0.61 eV. Lee et al.^116^ derived an activation energy of 0.6 eV from retention measurements of Pr_0.7_Ca_0.3_MnO_3_. Inge et al.^117^ performed DFT calculations for Pr_0.5_Ca_0.5_MnO_3_ and calculated the activation energy of vacancy migration in dependence of the migration path, ranging from 0.68 to 1.14 eV.

Since oxygen transport during decay consists of transport within the tunnel oxide, over the interface, and within the PCMO, this raises the question, of which activation energy must be considered. It is reasonable to assume that the highest activation energy will limit the kinetic. Since Kumbhare and Ganguly^115^ observed a strong dependence on the retention of the PCMO/W devices from the PCMO stoichiometry, it is reasonable that for this device, the activation energy of oxygen movement in PCMO is the speed-limiting process for their devices. Moon et al.^114^ compared Pr_0.7_Ca_0.3_MnO_3_ devices with different top electrodes (Al, Ti, Ta and Mo). They saw the highest retention of the LRS for the devices with Mo as top electrode and concluded that the activation energy for the oxidation of the top electrode is controlling the retention. Moon et al.^83^ further suggested an Al/Mo/PCMO based device as a compromise between improved retention due to MoO_*x*_ acting as an oxygen buffer layer and AlO_*x*_ to increase the on/off ratio.

However, the retention time of PCMO-based devices varies widely in the literature. In the same way as the measured activation energies of PCMO-based devices vary, also the measured retention times vary, from 10^–2^ s^114^ over 10^8^ s^116^ to 10^15^ s (.^115^) This is, on the one hand, based on the different activation energies for oxygen vacancy migration for different tunnel oxides and differences in the activation energies within PCMO between different stoichiometries and difference in layer growth. Further, retention values will also differ because of different SET or RESET pulse heights and lengths as well as read pulses. In addition, different definitions of retention time are used in relation to the resistance ratio, and a rigorous accelerated life study requires the use of different temperatures.^118^

Furthermore, these reports of retention are single device studies and therefore lack a high statistical base. An example of a statistical analysis of retention of a full memory block of filamentary devices was performed by Wiefels et al.^118^ which shows that the whole statistical distribution changes during accelerated life testing, thus only considering the shift of the mean do reflect the reliability of the devices. To our knowledge, no such studies have been published for PCMO-based devices.

Compared to filamentary devices, area dependent devices are generally considered to have lower retention and more read disturbance than filamentary devices.^3^ This could be due to less pronounced oxygen vacancy redistribution in the switching kinetics along the entire interface, as it is the case for highly temperature-accelerated switching of filamentary devices. However, as discussed in section 5.3, these limitations are not severe enough to stop the industrial interest in PCMO-based devices. Retention times can be controlled by engineering the ionic conductivities in the interfacial region, and the publicly traded company 4DS memory is advertising their devices with tunable retentions on the order of days to months.^85^

### 4.6 Endurance

The endurance of a ReRAM device is the number of cycles in which it can be reversibly switched between its resistive states. As an important reliability criterion for memory applications but even more crucial for the use of memristive devices for in-memory computing and for training in neural networks. For comparison, nonvolatile floating-gate memories such as Flash have an endurance of about 10^4^–10^5^ cycles, and volatile memories such as DRAM and SRAM have much higher endurance of 10^15^ and more. ReRAM memory could therefore fill the gap as a nonvolatile memory with a higher endurance than Flash memory.^119^

For filamentary VCM devices, as reviewed by Lanza et al.,^120^ there is a large gap between the endurance claimed by some academic research, measured on single devices, which goes up to 10^15^ cycles and the realistic endurance offered commercially for memory based on ReRAM technologies, which is in the order of ∼10^6^ cycles, considering the reliability of a full memory block. Therefore, Lanza et al.^120^ concluded that a reliable characterization of endurance requires characterization of a large number of devices rather than single device studies. An example of such a statistical investigation of the endurance of a complete 2 Mbit memory block of Infineon’s filamentary VCM devices was performed by Wiefels et al.^118^ Furthermore, as reviewed by Lanza et al.,^121^ many single device studies claiming high single device performance do not measure the resistive state after each switching event but only check a tiny subset. This leads to unrepresentative data as the measurement concept does not capture whether the device was stuck in a resistive state during the unmeasured cycles.

As far as PCMO-based devices are concerned, to our knowledge there are no endurance studies based on statistical evaluation of many devices. Furthermore, the published single device studies do not fulfill the characterization standards proposed by Lanza et al.^121^

However, Park et al.^122^ claim to achieve with their crossbar array based on AlO_*x*_/TiN/PCMO an endurance of 10^9^ cycles. Further Liao et al.^123^ claim, without providing data for Al/PCMO devices, endurance of up to 10^10^ cycles with PCMO provided by the company 4DS Memory. In addition, 4DS claimed in its official market announcements^85^ an endurance of over 10^9^ cycles for its PCMO-based megabit arrays. In the absence of scientific publications, these claims are not verifiable but support the hypothesis that area-based devices may have an endurance advantage over filamentary devices. These hypotheses arise from the fact that some of the failure mechanisms are based on the heating of the thermodynamically unstable filament and the effect of the heat on the surrounding atomic configurations.^119^ These failure mechanisms could be avoided using area-dependent switching devices. In addition, the switching of filamentary devices is based on the movement of a few oxygen atoms in a stochastic process,^121^ which could be inherently less reliable than the movement of many oxygen ions across an interface.

### 4.7 Reliability Aspects

In addition to retention (section 4.5.2) and endurance (section 4.6), cycle-to-cycle and device-to-device variability, as well as read noise and read disturb, are important reliability characteristics for the use of a memory arrays as a storage technology.

Cycle-to-cycle variability is intrinsically related to the stochasticity of the underlying switching kinetics and can further be used for assessing device-to-device variability. The switching kinetics of area-dependent PCMO-based devices are not considered to be as stochastic as the switching of filamentary devices, whose resistance state, especially in the LRS, can depend on the atomic position of a smaller number of oxygen vacancies.^118^ Thus, the cycle-to-cycle variability of area-dependent devices is generally considered to be lower than that of filamentary devices.^3^ For PCMO-based devices, this has been shown by Kumbhare et al.^115^ and Panwar et al.^124^ through their work on W/PCMO devices and comparison with other filamentary systems. As shown by Phadeke et al.^125^ for W/PCMO devices, this reduced cycle-to-cycle variability can be exploited for multilevel switching of PCMO-based devices, which is particularly useful for neuromorphic applications.

As mentioned in section 4.5, the disadvantage of low retention in PCMO-based devices results in a reduced tolerance of the resistive state to readout pulses. This read disturb is generally considered to be a problem of interfacial VCM devices.^3^ However, these reliability characteristics have not been extensively studied for PCMO-based devices. A study by Kumbhare et al.^126^ characterized the read disturbance of the HRS in W/PCMO devices at a high voltage of 1.75 V and claimed that the HRS is stable at this elevated voltage for up to 10 s, concluding that the HRS can handle a large number of short readout pulses.

Current measurements at read times and voltages below the amplitude of a read disturbance may also show variability, known as read-to-read variability. Since the cycle-to-cycle variability of PCMO-based devices is believed to be low, the read noise indirectly reflected by these measurements is expected to be comparably low.

Read noise in filamentary devices can be caused by fluctuations in individual defect states in the small plug region of the filament.^118^ In comparison, the effective area of current flow in area-dependent devices is much larger, so the relative influence of individual defect fluctuation is smaller. Area-dependent devices therefore have the potential for lower reading noise. However, this logic also implies an increase in read noise with smaller device sizes, which has not yet been investigated for PCMO-based devices. Studies of the read-to-read variability of PCMO-based devices are rare. Lee et al.^127^ investigated the read noise of Al/PCMO devices and found the characteristics of random telegraph noise in the HRS/LRS and a relative noise ΔI/I of (13%/3%) at −1 V readout and (6.3%/4.4%) at −0.6 V. However, apart from this study, more detailed investigations of this read-to-read variability of PCMO-based devices are needed, considering a larger amount of manufactured devices, readout parameters and a larger statistical base.

The device-to-device variability can be used to assess the quality of a manufacturing process in terms of homogeneity and reproducibility, since the cycle-to-cycle variability of PCMO-based devices is generally considered to be low. In addition, for well controlled manufacturing processes, the device-to-device variability also assesses the sensitivity of the switching characteristics to small variations within the manufacturing process. To assess the device-to-device variability of a manufactured crossbar array, the cumulative probability distribution for HRS and LRS of a selected subset of devices can be plotted and compared. This has been done in several studies of crossbar arrays. Cho et al.^128^ showed for their N:TiN/PCMO based arrays the cumulative probability of 30 devices from each array at three different locations of the arrays. Hong et al.^129^ showed for a TiN/PCMO based array the cumulative probability along one diagonal of the array. Park et al.^130^ showed for their Al/PCMO based arrays the cumulative probability distribution of 20 devices at 5 dies from different locations of the fabricated wafer. All three studies show a sufficiently small variation in the probability distribution to clearly separate the LRS and HRS resistivity regions. These studies demonstrate that the sensitivity of the switching characteristics of PCMO-based devices to manufacturing variations is sufficiently low to enable array implementation of these devices. However, the studies shown do not provide a full statistical analysis based on a complete analysis of the entire memory array, as was done, for example, by Wiefels et al.^118^ for filamentary devices.

## 5 Neuromorphic and Memory Applications

Generally, area dependent devices have two main advantages over filamentary devices, that make them interesting for neurons or synapses in artificial neural networks (ANN). First, in contrast to filamentary memristive devices, area-type switching devices display a more gradual SET process. This improves their ability for analog switching and eliminates the need for a current compliance.^4^ Second, because the device resistance scales with the device area, its values can be tailored to specific circuit requirements. Another benefit is the nonlinearity of the *I–V* characteristics of most PCMO devices, which makes them suitable for passive, self-select arrays. This strategy decreases parasitic leakage currents and thus results in a more straightforward array design.^5^

This section will review the technical applications and possibilities for addressing potential imperfections of area-dependent PCMO-based devices.

### 5.1 Neuromorphic Simulations Based on Single PCMO Devices

Studies of large arrays of PCMO devices, integrated into CMOS circuits, are rare. However, network simulations exist, which are based on electrical measurements of single PCMO devices as well as models based on the characterization of PCMO-based device arrays. This can be used to prove the possible application of PCMO devices for ANNs like feedforward neural network (FNN), spiking neural networks (SNNs) or convolutional neural network (CNN). These studies will be presented below.

#### 5.1.1 Potentiation, Depression, and Associated Neuromorphic Performance

The driving motivation for using PCMO devices in ANNs, such as FNN or CNN, is to use them to perform vector-matrix multiplication in a single step in crossbar arrays utilizing Kirchoff’s-law. In this way, the high energy consumption of training and inference of AI models, especially caused by the data transfer between logic operation and data storage in a von-Neumann architecture is reduced by the parallelization of computation and memory.^2,131,132^

To modify the synaptic weight and train the network for inference, the resistance must be adjusted incrementally, called potentiation for increasing conductivity and depression for decreasing conductivity. It can be regarded as the hardware implementation of the biological phenomenon that repeatedly activated synapses become stronger with increasing activation, called long-term potentiation (LTP),^133^ or weaker due to less activation, called long-term depression (LTD).

For potentiation or depression, voltage pulses of a certain height and length are applied to the devices. The pulse must be short and low enough to cause only a partial change in the resistance state. Repeated applications of the same pulse can be used for continuous resistance control.

The usability of the potentiation and depression characteristics of a memristive device can be judged by three quality criteria: symmetry, linearity and number of states. The ideal device should respond symmetrically in its conductance change to a potentiation or depression pulse. In addition, the change in conductance should be independent of the current conductance state of the device, i.e. linear and with an appropriate slope to allow gradual tuning of the resistance. In addition, the utility of the device increases with the number of resistance states that can be discriminated.

For PCMO-based devices, the threshold voltages for achieving the potentiation (SET) and depression (RESET) are asymmetric depending on the Gibbs energy Δ*G*_0_ for the formation of the tunnel oxide involved (section 3.2). For a higher Δ*G*_0_ the oxidation of the tunnel oxide during the depression is thermodynamically facilitated, so that the RESET voltage decreases, and the SET voltages increase.^108^ From the perspective of the oxygen vacancy movement, this asymmetry of oxidation and reduction leads to an asymmetric barrier and thus a different drift velocity.

Control of the drift velocity of oxygen movement during potentiation and depression is essential for a linear conductance change. As shown by Dittmann et al.^112^ for thick-filament devices, reducing the heating by a series resistor and thus controlling the drift velocity allows a more gradual tuning of the resistance. Since the kinetics of PCMO-based systems are not temperature controlled (section 4.5) but field controlled, control of the drift velocity in these devices is mainly a matter of matching the field drop to the resistive state. This can be achieved by parallel and serial resistors or by adjusting the voltage level. However, the linearity is not only controlled by the drift velocity, as the resistance does not necessarily change linearly with the amount of redistributed oxygen vacancies.

Material stack selection can reduce voltage asymmetry. Lee et al.^108^ found a higher resistance ratio during switching for higher Δ*G*_0_ and therefore argue that the choice of material stack is a trade-off between reducing asymmetry and reducing the switching ratio and thus the number of distinguishable resistance states. Cho et al.^128^ and Lee et al.^8^ therefore consider TiN/PCMO as the stack with the best trade-off between on–off ratio and asymmetry for neuromorphic applications. The current and the asymmetry in the *I–V* loop of TiN-based devices can be reduced by increasing the ratio of N_2_ to Ar during the deposition. According to Park et al.^84^ the increase in nitrogen content is increasing the work function of the TiN_*x*_ and therefore decreasing the Schottky barrier between PCMO and TiN_*x*_. Park et al.^122,134^ claimed to achieve symmetric potentiation and depression at symmetric pulse heights (± 3 V, 1 ms pulse length) using an AlO_*x*_/TiN/PCMO stack. Here, the AlO_*x*_ acts as an internal resistor, avoiding strong field droping across the TiN in the LRS and enhancing the TiN/PCMO stack.^8^

The cointegrate of the device with a serial resistor (Figure 43b) in order to reduce synaptic asymmetry was also proposed by Lee et al.^135^ and Dittmann et al.^112^ The serial resistor has the advantage of reducing the effective voltage drop across the resistor in a low resistance state. Thus, the effective pulse height continuously increases during the depression pulses and decreases during the potentiation (Figure 43c). The reduced voltage at the beginning of the depression compensates for the higher voltage sensitivity during both depression and potentiation, thus reducing the asymmetry. Lee et al.^135^ tuned this resistor together with a resistor parallel to the device for an TiN/PCMO/Pt device (Figure 43a), optimizing the asymmetry to a near-ideal conductance ratio after the same amount of applied pulses (Figure 43d). Using multilayer perceptron-based simulations, Lee et al.^135^ showed that the improvement in asymmetry can lead to an improvement in pattern recognition. Cho et al.^128^ further demonstrated improved recognition performance for using the symmetric synaptic weight in a deep neural network to recognize contextual fear memory from locally recorded brain waves of rats (Figure 43e). To use this approach for implementation in a neuromorphic circuit, the resistance values of the deposited devices must be well controlled, as the effect of a tailored parallel resistance is sensitive to the resistance of the memristive device. In addition, the method has the side effect of reducing the voltage during the later pulses of the potentiation, which can lead to a reduction of the nonlinearity during the potentiation.

**Figure 43:**
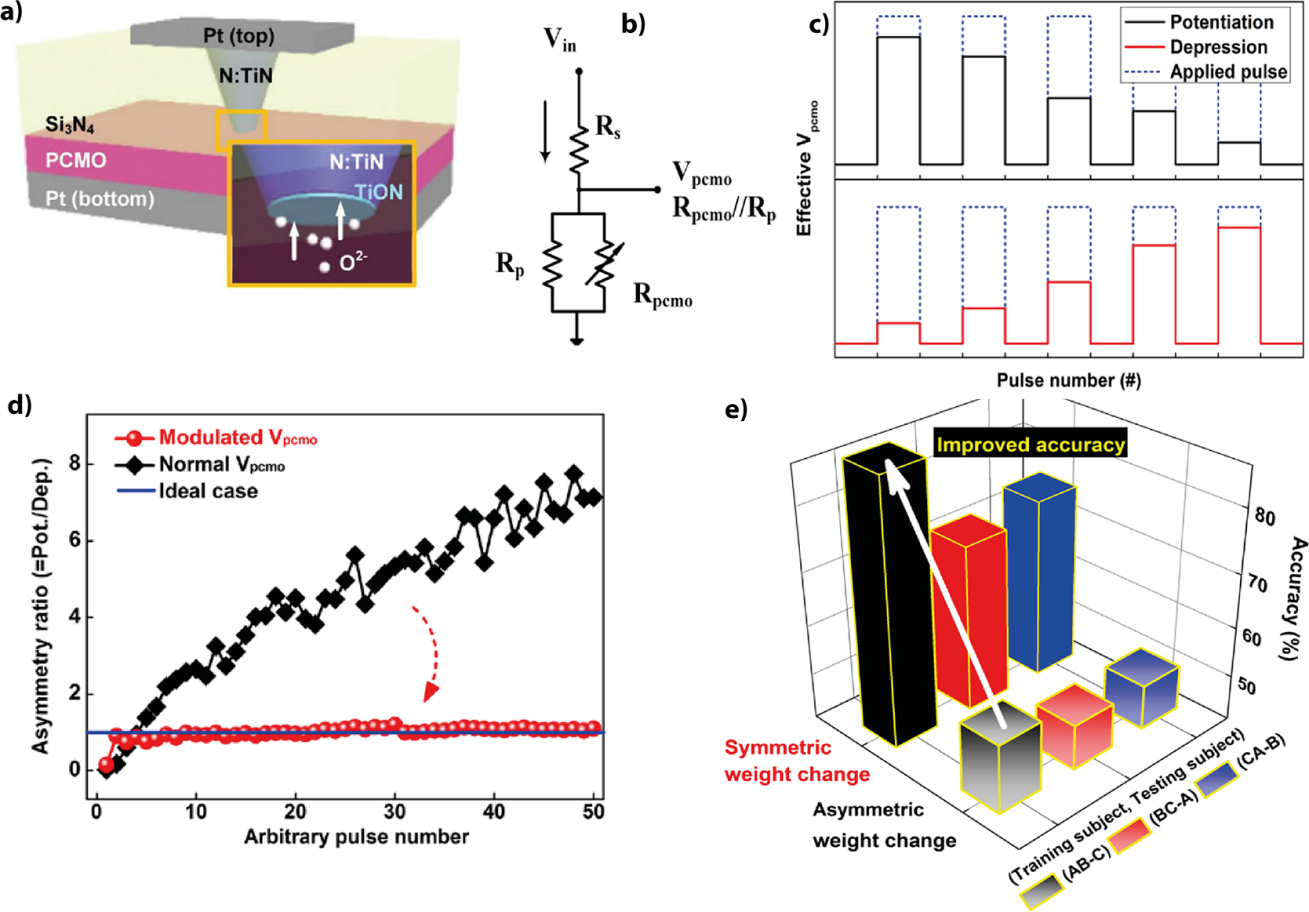
a) Via hole structure of one PCMO-based memristive device from the 8K-bit array used for biomedical application. Adapted and reprinted with permission from^108^ © 2015 by AIP Publishing. Adapted and reprinted with permission from ref 128. Copyright 2024 by Springer Nature. b) Schematic overview of the circuit, which modulates the voltage drop as displayed in c). d) Comparison of the asymmetry of the conductance during potentiation and depression with and without the modulation of the voltage. e) Improved fear recognition accuracy of the PCMO synapse of three different training approaches. The black pillar presents the accuracy after the data from the measurements of rat A and rat B were used for training the system, and rat C was the tested subject. The same principle applies to the other cases. Adapted and reprinted with permission from ref 128. Copyright 2024 by Springer Nature.

To increase the symmetry, asymmetric voltage pulses can be selected for potentiation and depending on the Δ*G*_0_ of the material stack. Some groups find a much more abrupt behavior in the resistance change during depression and a more gradual resistance change during potentiation for TiN/PCMO (1 ms pulse length, Figure 45)^128,135,136^ and Al/PCMO (10 ms^137^ and 1 ms^122^ pulse length). Using a shorter pulse length of 100 μs Gutsche et al.^62^ were able to induce a more gradual depression in Al/PCMO, TaO_*x*_/PCMO and WO_*x*_/PCMO devices (Figure 44).

**Figure 44:**
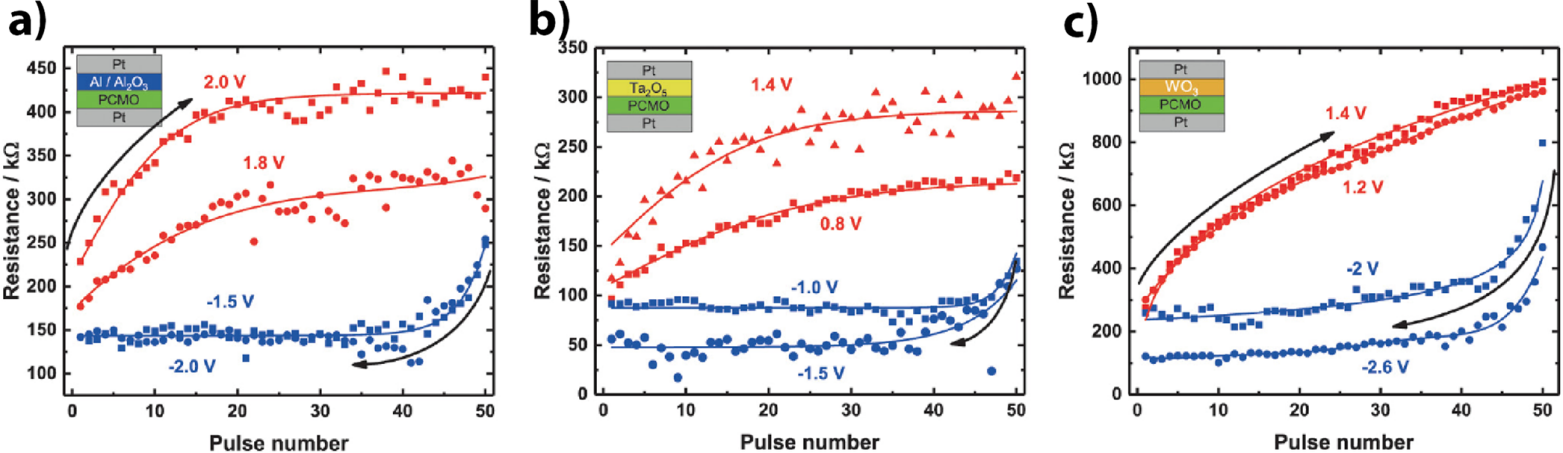
Potentiation and depression measurements on different PCMO-based devices. a) Al/Al_2_O_3_, b) Ta_2_O_5_, and c) WO_3_. Taken from ref 62.

Nonlinearity can be improved by gradually varying pulse trains with continuously increasing pulse height or pulse length can also be used to achieve a more gradual switching, as shown for example for Al/PCMO devices by Park et al.^122^ The use of state dependent voltage pulse heights for potentiation and depression, however, is an additional circuit design requirement for a neuromorphic application and thus a technological barrier.

Asymmetry can also be avoided by using only potentiation or depression through special circuit design. Sheri et al.^137^ for example avoided the asymmetry by representing a synaptic weight by a ″two-memristor structure″. Since potentiation is the more gradual process, as discussed above, the conductance of both devices is changed in the direction of potentiation to adjust the synaptic weight. By temporally decoupling the readout of both devices by an external clock, the current through both devices can be measured in two different clock cycles and then subtracted by an external logic. Thus, the difference in conductance of the two devices serves as the synaptic weight. Since the weight change of these two-memristor devices is limited once the potentiation curve of one device is saturated, this circuit design requires the implementation of a so-called sleep operation in addition to the read and write operation. This operation must be applied when a device is saturated and therefore insensitive to a write operation. The sleep operation reads out the effective memristive weight representing the two devices. Then both devices are fully reset, and the effective weight is stored so that the total potentiation of both devices is minimal. Sheri et al.^137^ used the potentiation and depression curves of an Al/PCMO device and the simulation of a feedforward network to prove the concept of image recognition even when noise in the potentiation and depression curves is considered. However, the disadvantage of this method is that the temporal separation of the two memristors precludes its use for spike-time dependent algorithms. In addition, the need for sleep operation makes it difficult to scale this approach to large networks.

The effect of the nonlinearity can be corrected using a quantization algorithm. For example, Lee et al.^136^ proposed to quantize the potentiation and depression curves into certain intervals and then select a subset of the conductance values (Figure 45a) such that the subset has a more linear distribution of conductance values than linear selection by pulse number (Figure 45b). They claim that their quantization method can be used directly for on-chip learning. Lee et al. applied this method to the potentiation and depression curves of a Pt/PCMO/TiN/Pt device (Figure 45e) and compared the detection accuracy of a neural network based on optimized (Figure 45d) and unoptimized (Figure 45c) selection of conductance values. The optimized selection of conductance values leads to an improvement in classification accuracy for the MNIST data set, especially when the total number of selected conductance values was low. The implementation of this method in on-chip training led to the challenge that the training circuit must read out each synaptic weight before training and then apply the individual necessary number of pulses according to its resistance state to stay within the selected set of conductance values. Thus, correcting the nonlinearity in the manner proposed by Lee et al.^136^ increases the complexity of the circuitry.

**Figure 45:**
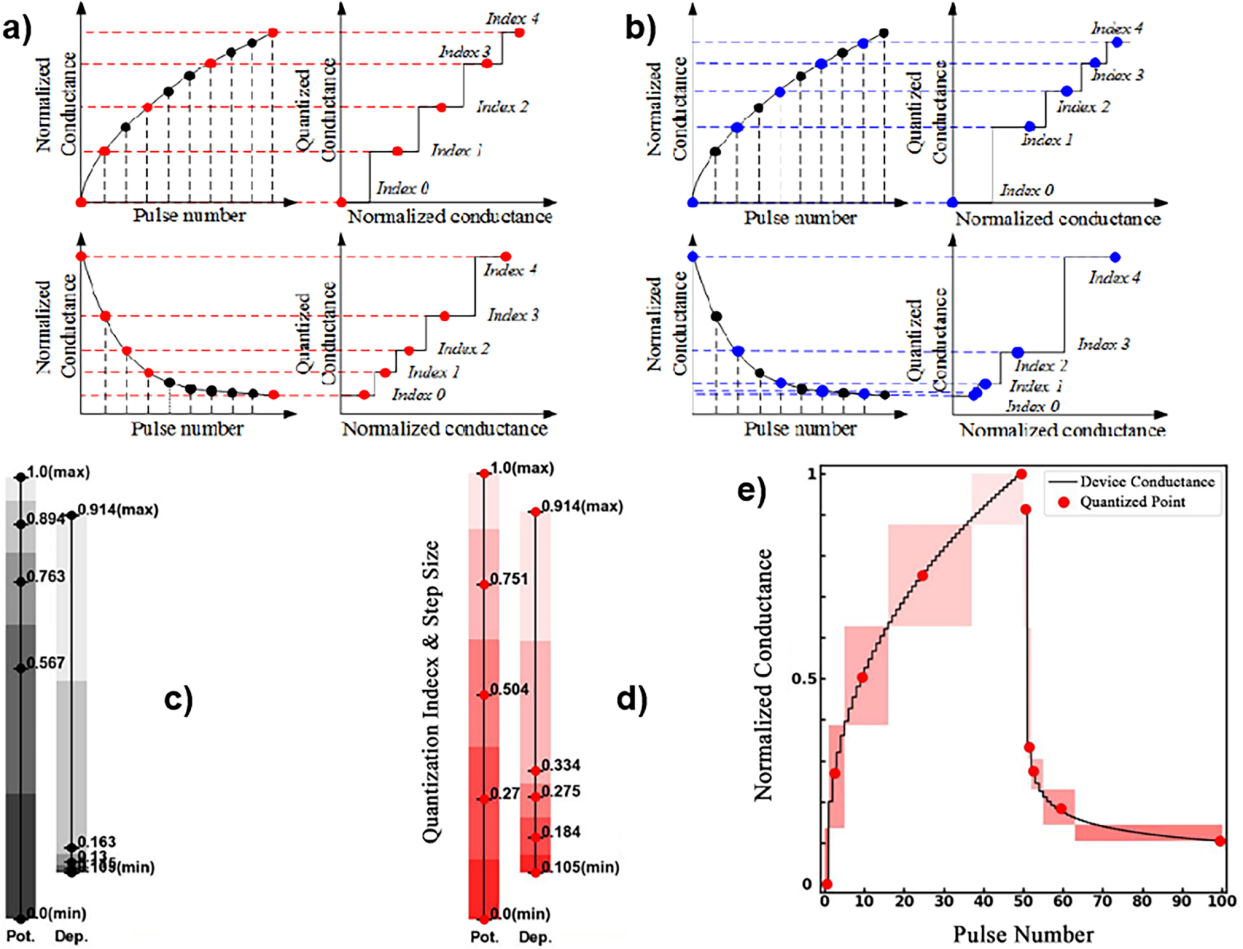
a) Proposed conductance quantization method, b) compared to regular pulse intervals. Distribution of normalized conductance values of Pt/PCMO/N:TiN/Pt devices for c) nonoptimizes and d) optimized conductance selection. e) optimized conductance selection at the potentiation and depression curves of the Pt/PCMO/N:TiN/Pt devices. Taken from ref 136.

A common approach to training the ANN is to optimize the influence of the synaptic weight on a loss function using gradient descent learning. A challenge in implementing gradient descent learning using potentiation and depression pulses is beside asymmetry also the nonlinearity of the resistance changes with the pulses.^138^ A simple circuit implementation that does not consider the resistive state of the memristive element leads to a state-dependent learning rate, which could negatively affect training. To address this issue, Gutsche et al.^62^ used the batch-based training proposed by Gao et al.^138^ and accumulated the backpropagation of 60,000 MNIST data sets for each training epoch to reduce the effect of nonlinearity on individual backpropagations. Furthermore, pulse-based training of a memristive element does not consider the strength of the training signal, which is given by the size of the gradient in the backpropagation. Therefore, Gutsche et al.^62^ only trained the weight with the highest gradient for each layer for the network.

Gutsche at al.^62^ fitted the potentiation and depression measurements as shown in Figure 44 depending on pulse height and length with a logistic function and simulated a four-layer perceptron network with a matrix of the PCMO-based memristive devices used as synaptic weights. Using this model, they evaluated the dependence of the detection accuracy on the nonlinearity and the on/off ratio of the potentiation and depression curves for Al/PCMO, WO_*x*_/PCMO and TaO_*x*_/PCMO devices. Interestingly, the Al/PCMO device gives the highest recognition accuracy due to the combination of high on/off ratio and high nonlinearity. This shows that the nonlinearity of potentiation and depression of PCMO-based devices, is not necessarily problematic for the application in neuromorphic computing as the influence of nonlinearity is highly dependent on the specific implementation.

#### 5.1.2 Spike-Timing-Dependent Plasticity and Associated Neuromorphic Performance

The previous discussed approach of using potentiation and depression of memristive elements in the framework of FNN and CNN are non–von Neumann approaches of parallelized in-memory computing. However, this approach often mimics current AI technology based on central clock-synchronized information flow and time-multiplexed processing. A more brain-inspired approach is spiking neural networks, which encode information through the timing of spikes. This concept holds promise for low-power edge devices by decoupling signal processing from an internal high-frequency clock and instead using external, real-world, event-based, low-frequency time scales.^139^

One biological learning rule that can be used for the realization of SNNs is spike-timing-dependent plasticity (STDP), which is a realization of the Hebbian learning rule. STDP has been showcased on single PCMO devices.^62,140,141^ STDP is the modification of synaptic weight Δ*G* depending on the relative timing Δ*t* of neuronal spikes. This behavior is emulated through pulses using timed pre- and postsynaptic spikes. If *t*_pre_-*t*_post_ < 0, the conductance is enhanced, and if tpre-tpost > 0 it is reduced (cf. Figure 46).^62^ Influencing the device switching that way means that voltage pulses must be used, where the pulse form is added in a SET supporting way if *t*_pre_-*t*_post_ < 0 and in a RESET supporting way for the opposite time difference. This concept is based on the nonlinear increase in switching with applied voltage and requires that the duration of each pulse be longer than the total time difference between the pulses. STDP measurements are highly dependent on the shape of the pulses applied. The total overlapping pulse signal causes potentiation or depression of the synaptic weight. Since the effect of a voltage pulse on potentiation or depression depends on the conductance value of the device, a complete characterization of STDP for a given pulse shape must consider the dependence on the initial conductance state of the device.

**Figure 46:**
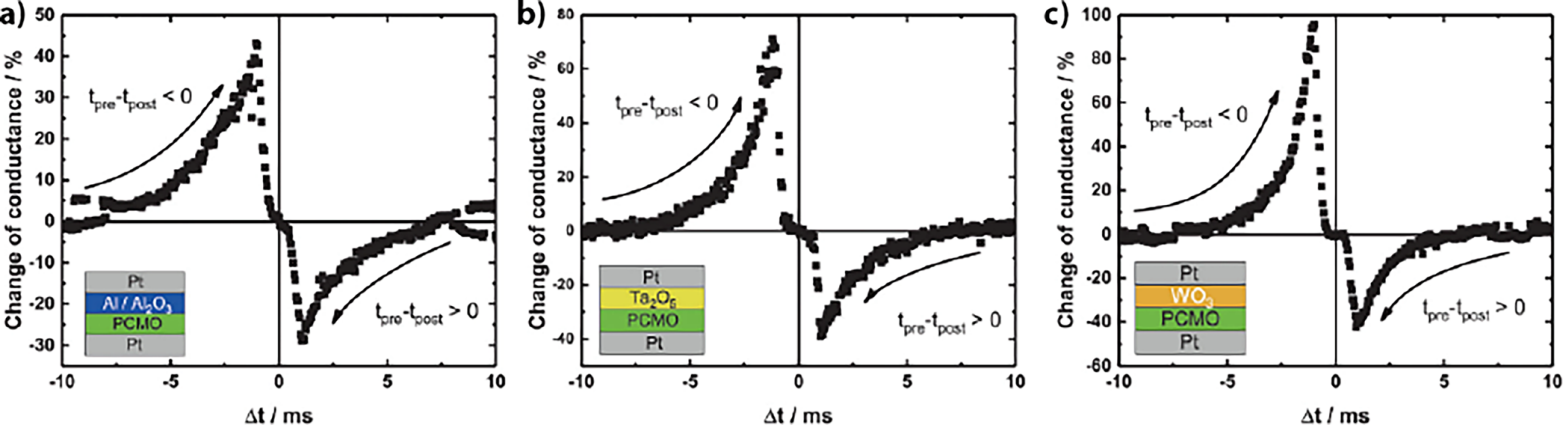
Relative change in conductance as a function of varying time delays between pre- and postsynaptic pulses during STDP measurement is depicted for three devices: (A) Al/native Al_2_O_3_, (B) Ta_2_O_5_, and (C) WO_3_. Taken from ref 62.

Gutsche et al.^62^ showed STDP-like behavior on PCMO-based devices with three different TO layers (Al/Al_2_O_3_, WO_3_, and Ta_2_O_3_). The relative change of the conductance is displayed in Figure 46.^62^

A more detailed analysis of the STDP, including its dependence on the initial conductance, was performed by Shukla et al.^140^ for a W/PCMO device. They used exponentially decaying symmetric spikes with spike heights of + −1 V and asymmetric exponential decay times of 50 and 0.5 μs, and asymmetric spike durations of 1 μs and 100 ms. Shukla et al.^140^ also performed training of a single-layer SNN trained with STDP for classification. From these simulations, they concluded that a low maximum learning rate of 2%, especially for depression, is important to achieve high classification accuracy. The maximum learning rate is the maximum achievable Δ*G*/*G*, where *G* is the conductance of the synaptic weight. Since, according to their analysis, this low learning rate is not achievable with a single synaptic device, Shukla et al.^140^ recommend multiple (*N*) parallel PCMO devices acting as a single synapse to improve the learning rate of PCMO-based devices, resulting in a learning rate of Δ*G*/G * N. However, since this proposed limitation of SNN performance by the maximum learning rate is derived from their implementation of a single SNN accuracy, a modification of SNN implementations might not show the same limitations.

### 5.2 PCMO-Based Device Arrays for Neuromorphic Computing

In the previous section, simulations showcased how PCMO-based devices could act as synaptic weights. In this section, we will present ANN simulations and on-chip computing based on memory arrays. The integration of PCMO-based arrays into crossbar arrays is a building block to achieve vector-matrix multiplication using Kirchhoff’s laws. However, this approach is limited in the possible array size due to the sneak path current problem, which scales with the size of the array. To avoid this problem, each memristive element must be cointegrated with a transistor or selector.^2^

#### 5.2.1 Challenges of CMOS Cointegration

The required back-end of-the line (BEOL) integration into CMOS technology circuits is challenging because PCMO must be deposited at elevated temperatures to grow in a crystalline phase, which is considered necessary for low-voltage bipolar resistive switching.^100,142^ Temperatures above 400 °C are considered incompatible with the CMOS back-end of the line.

Crystalline growth below 400 °C by sputter deposition has been achieved by a special back-biased face-target sputtering process by the company 4DS.^48,58,59,123,143^ Normal RF sputter deposition at room temperature shows only a very low degree of crystallinity as shown by Kanegami et al.^102^

Pulsed laser deposition (PLD) has not been able to achieve high-crystallinity, which means significant strong Bragg reflections, at CMOS-compatible temperatures. Different crystallization temperatures have been reported depending on the substrate material. Yamamoto et al.^104^ reported a low intensity of Bragg reflection for deposition at 600 °C on MgO (001) single crystal substrate and amorphous SiO_2_ substrate, with a lower degree of crystallinity for deposition on amorphous SiO_2_ compared to crystalline MgO. Kim et al.^144^ showed that low intensity Bragg reflections are already visible when deposited on a crystalline Ag bottom electrode at 400 °C. Seong et al.^105^ saw no evidence of crystallinity for deposition on a polycrystalline Pt bottom electrode at 500 °C and below. Panwar et al.^142^ also saw no evidence of crystallinity for deposition on a polycrystalline Pt electrode at 450 °C.

So, to our knowledge, the best way to achieve BEOL integrated crystalline PCMO is a special sputtering process. The alternative is the use of amorphous PCMO, which is less studied. There are some studies showing area dependent switching for PCMO grown at room temperature by pulsed laser deposition (PLD), namely for ITO/PCMO,^107^ Ti/PCMO,^103^ and AlO_*x*_/PCMO.^100^ However, there are also studies showing different switching behavior with room temperature PLD grown PCMO, such as filamentary switching for ZnO,^106^ unipolar switching for W/PCMO^142^ and a combination of filamentary and area dependent switching for Ti with room temperature sputtered PCMO.^102^

#### 5.2.2 Sneak Current Challenge for Crossbar Arrays

The difficulties described in section 5.2.1 limit the research community for true CMOS cointegration of PCMO-based devices. This makes it difficult to avoid the crosstalk problem by a sneak path current without 1T1R integration. Some groups claim that the strong nonlinearity and of the *I–V* loop of a PCMO-based device can be used for a selector-free array without read interference, a concept which is limit in scalability.^5^ A simple concept to exploit the nonlinearity is to select the cell by applying half the read voltage to each line, so that the nonselected cells of the selected lines are only biased by half the voltage and a strong nonlinearity can reduce the sneak path current (Figure 47a).

**Figure 47:**
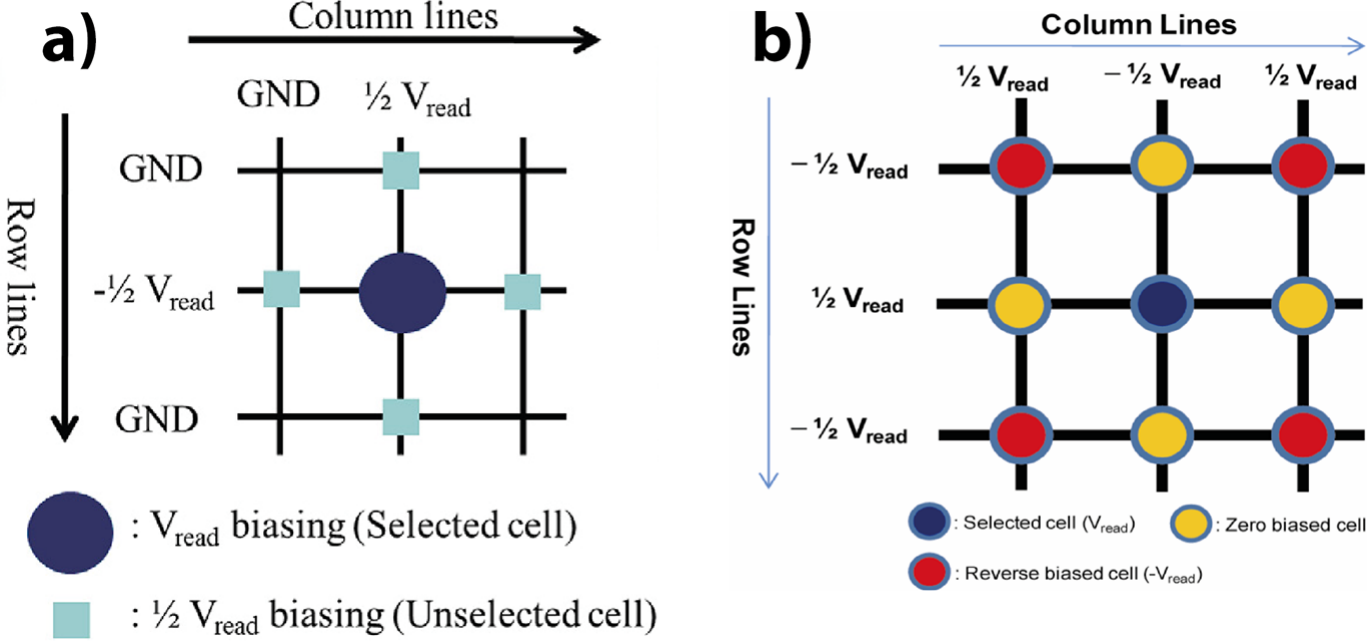
a) Simple readout scheme using half voltage selection to exploit nonlinearity. Adapted and reprinted with permission from ref 145. Copyright 2011 by Elsevier. b) Advanced readout scheme that further exploits asymmetry. Adapted and reprinted with permission from ref 146. Copyright 2010 by IEEE.

Jo et al.^146^ and Lee et al.^145^ proposed to use the asymmetry of the *I–V* loop of Al/PCMO devices to reduce read interference by using an alternating readout scheme and demonstrated it on a 50 nm Al/PCMO hole device. This readout scheme is based on the concept that only the selected device has a readout voltage in the forward direction, all devices in the same row and column line are effectively unbiased, and all other devices have an effective voltage in the reverse direction and therefore do not contribute to the overall readout. This is achieved by applying half the readout voltage to the row and column lines of the selected device with the sign of the forward direction and applying a voltage of opposite sign to all other lines (Figure 47b). The functionality of this readout scheme to reduce readout noise as well as set and reset noise was demonstrated by Park et al.^147^ for a W/Ti/PCMO device in a crossbar array. However, this concept can only be used for single device readout and is not helpful for the vector matrix multiplication application where the entire array is read out in one step.

Another concept for writing and reading selector-free arrays was presented by Chevalier et al.^148^ and Meyer et al.^5^ of Unity Semiconductor for the 64 Mb implementation of their CMO_*x*_ technology^73^ based on PCMO.^5^ Their concept is based on line-wise writing and reading, where each device along a given word line is processed at each interval. For writing and erasing, strong linearity is exploited by applying half of the switching voltage to the word line and half of the switching voltage to all bit lines of the targeted device. For reading, the current of all bit lines is measured simultaneously for each word line. An amplification unit is implemented to handle the small readout currents (device size 0.17 μm). A problem with the small readout current is that it limits the readout speed to 100 μs per Word line and the maximum read speed at the presented chip to 100 MB/s. In addition, the readout voltage must compensate for process variations in tunnel oxide thickness or external temperature changes to achieve a given readout current, as the electronic transport is very sensitive to both parameters. Therefore, an additional reference circuit is implemented to tune the readout voltage between 100–200 mV. This work by Chevalier et al.^148^ is, to our knowledge, the only work published on CMOS cointegration of a PCMO-based memory array and handles 1000 word lines and 1000 bit lines per array. After the acquisition of Unity Semiconductor by Rambus Inc. in 2012, this work continued until at least 2014. Arrays up to 8192 × 256 have been realized.^5^ However, as already mentioned the size of these selector-free arrays is limited. In this concept, the maximum number of word lines is limited by the sneak current during writing, and the maximum number of bit lines is limited by the sneak current during reading.^5,148^ This results in an asymmetric scaling limit on the number of word and bit lines for this concept.^5^

Since the nonlinearity in the *I–V* characteristic of PCMO-based devices already reduces the sneak current problem, Lee et al. proposed to increase the nonlinearity of Al/PCMO device by a CaMnO_3_ interlayer in PCMO to further improve the crosstalk problem.^149^ The nonlinearity can be further increased by the cointegration of a selector device. Lashkare et al.^150^ cointegrated an npn-selector with a W/PCMO device and showed the avoidance of sneak current below the threshold voltage of the selector.

#### 5.2.3 PCMO Crossbar Arrays and Neuromorphic Applications

Due to the difficulties of crystalline growth (section 5.2.1), to our knowledge no academic research group, but only companies such as Unity/Rambus and 4DS, have been able to integrate PCMO-based devices with CMOS technology. Thus, in the field of neuromorphic applications, the crossbar arrays have been fabricated as stand-alone devices, and neuromorphic applications have been achieved either by connecting the device to external circuitry or by exploiting the switching characteristics of the arrays in neuromorphic simulations.

Park et al.^130^ fabricated a 1 kbit crossbar array based on W/Al/PCMO devices. This crossbar array was connected to an external circuit to serve as a simplified building block for testing a device as a synapse, along with a connected neuron, which was trained and tested in HRS and LRS. Based on these device characteristics, the feasibility of this concept to classify number images (Figure 48b) was demonstrated in a circuit-level simulation.

**Figure 48:**
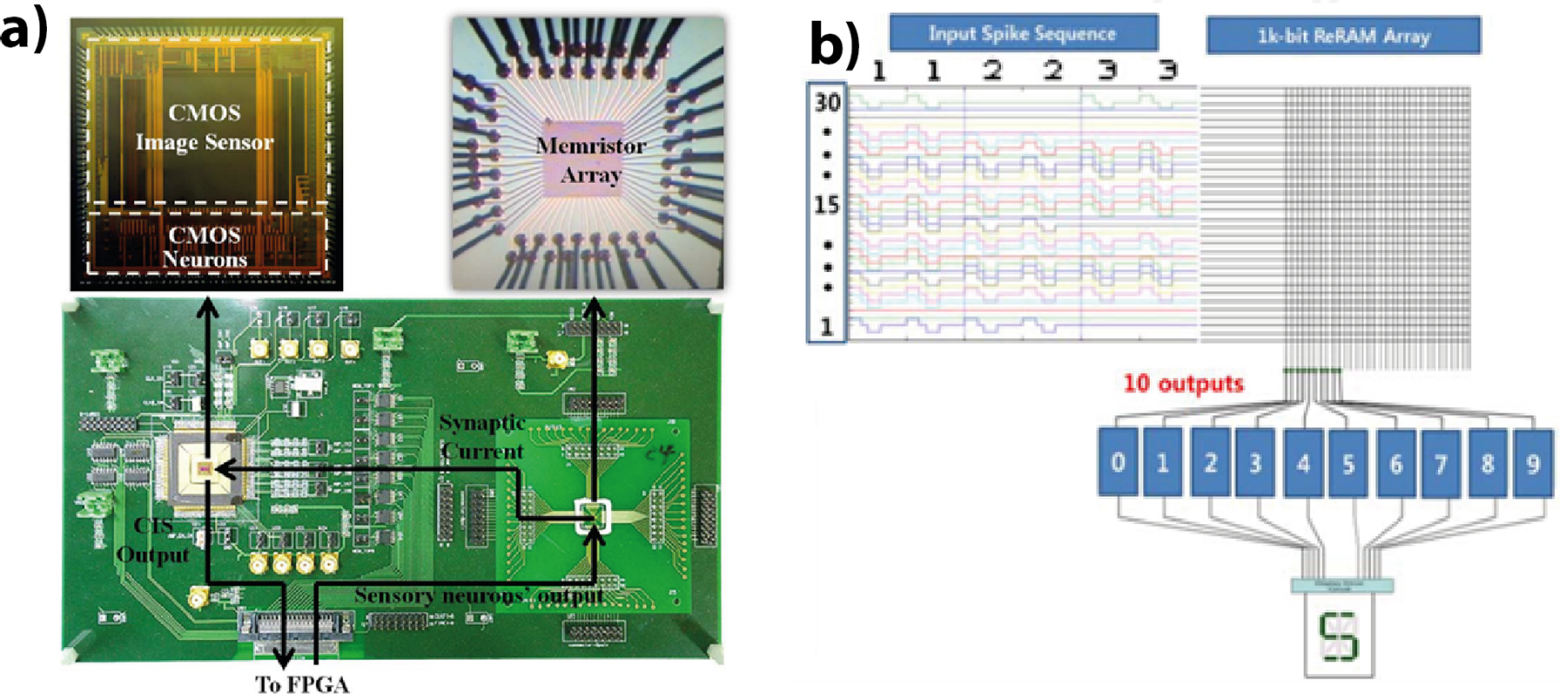
a) Image sensor, CMOS neurons, and memristive array implemented in one system. Adapted and reprinted with permission from ref 141. Copyright 2015 by IEEE. b) Schematic illustration of the image recognition system combining the 1k bit RRAM array and the CMOS neurons. Adapted and reprinted with permission from ref 130. Copyright 2012 by IEEE.

Chu et al.^141^ used a TiN/PCMO-based crossbar array and showed a complete experimental realization (Figure 48a) of number image classification. The input was a CMOS sensor combined with an FPGA to convert number images into a digital representation of 5 × 6 pixels. The 30 pixels were then converted into voltage pulses used to train or test the crossbar array, which forms the synaptic layer of a simple single-layer classification network.

There are multiple publications on groups who fabricated crossbar arrays, while only using the single device characteristics for simulating ANN tasks. For example Cho et al.^128^ fabricated chips of 8 K-bit array on an 8-in. wafer based on TiN/PCMO and used the single device characteristic for the simulations discussed in section 5.1.1 (Figure 43).

Hong et al.^129^ also fabricated a TiN/PCMO-based array (1 Kbit). From this array, a subset of 3 devices sharing the same bit line and 3 different SET voltages were used to realize 8 different conductance values. These device features were then used in a 3 × 3 weight kernel for edge detection in a CNN (Figure 49b).^129^

**Figure 49:**
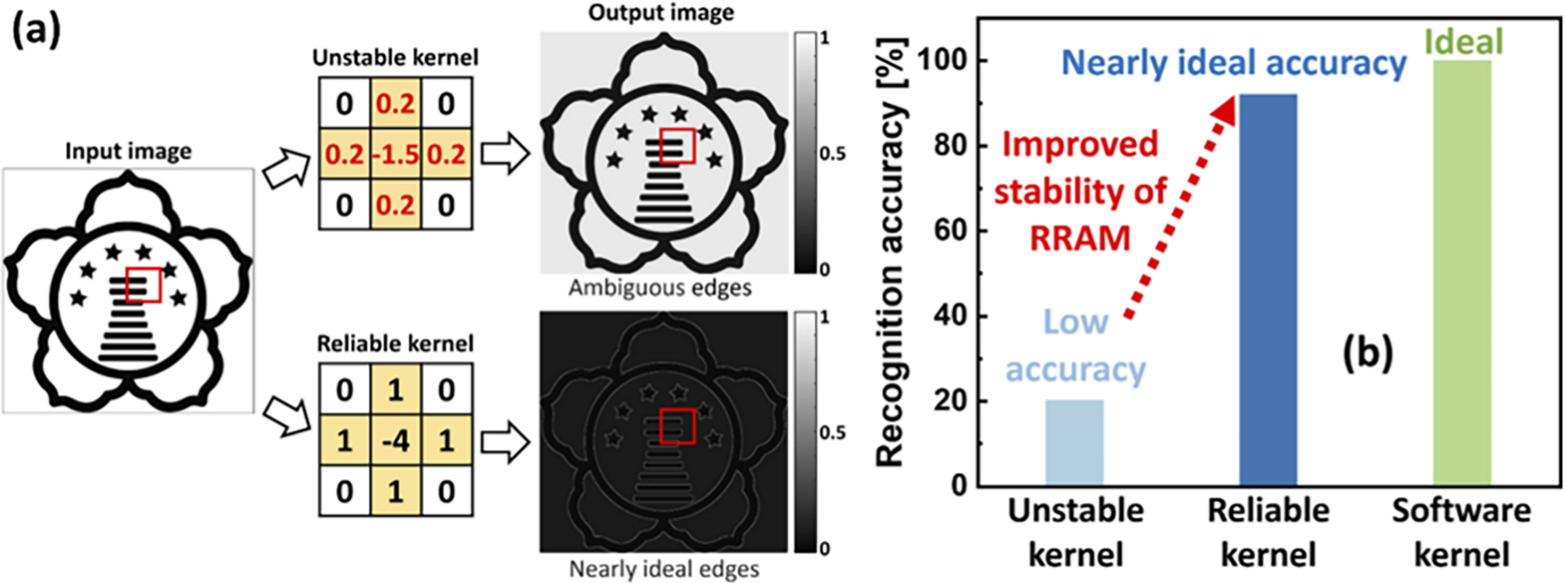
(a) Comparison of the image recognition with an unstable kernel due to unstable conductance states and an improved stable kernel and (b) the corresponding recognition accuracy. Taken from.^129^

Park et al.^122^ also fabricated 1 Kbit arrays, one based on Al/PCMO and another based on AlO_*x*_/TiN/PCMO. As discussed in section 5.1.1, the AlO_*x*_/TiN/PCMO-based array was superior in symmetric potentiation and depression, so its properties were further used to simulate the performance of a SNN task and for a hardware integration into a neural network. In the simulation, the devices were used as synapses for a feed-forward SNN trained for pattern recognition on the response signal of a cochlear implant to the sound of three Korean vowels, “a,” “I,” and “u,” and on the electroencephalogram (EEG) caused from this vowel.^122^ In addition, the arrays developed were fully integrated with CMOS-hardware and used for a single layer neural network to classify the EEG data according to the vowels.^134^

### 5.3 Industrial Application

Established memory technologies on the market can be categorized using a hierarchical structure (Figure 50) based on access time and capacity, which are generally inversely related, resulting in a symbolic pyramid shape. Resistive switching memory is ranked between Flash and DRAM based on its access time. With faster switching times than Flash, potentially higher endurance and retention, and a simpler structure with potentially better scaling potential for small nodes, it was seen as a potential competitor. However, despite its promising characteristics, the industrial process of Flash memory is superior in storage capacity and cost per bit due to continuous scaling and has prevented resistive switching memory from gaining a foothold in this market.^120^ Compared to DRAM, ReRAM can be nonvolatile and can achieve similar switching speeds but has the disadvantage of finite endurance. ReRAM is therefore being considered as a ″storage class memory″ to complement DRAM as a nonvolatile, fast and low-power memory. However, initiatives such as Intel Optane have not yet been able to establish a working business model. Today, companies such as Infineon, TSMC and Weebit Nano are offering their ReRAM technology for the edge device market, taking advantage of low power consumption and the ability to integrate BEOL for system-on-a-chip applications.^120^ However, devices with area-dependent switching mechanisms based on oxygen vacancies, such as PCMO-based devices, have not yet been used on a large scale in commercial products. Two major industrial attempts are well reported.

**Figure 50:**
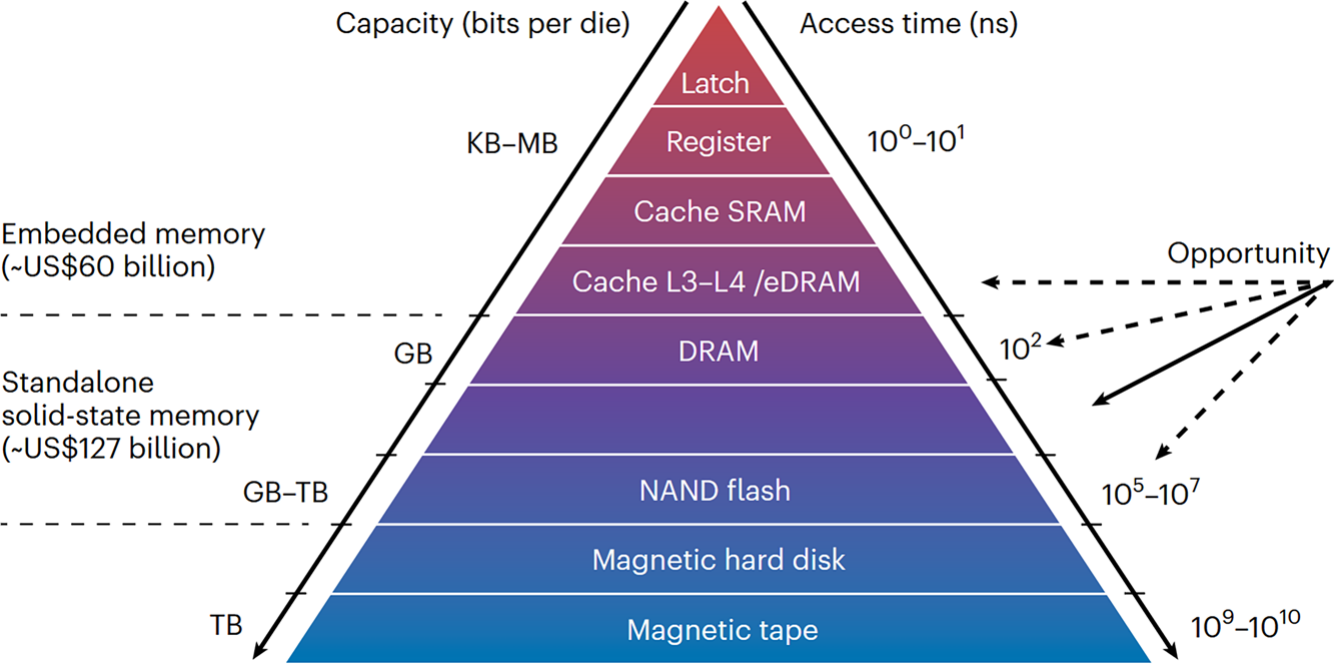
Hierarchical ordering of memory according to access time and storage capacity. Adapted and reprinted with permission from ref 120. Copyright 2023 by Springer Nature.

As discussed in section 5.2.2, Unity Semiconductor (acquired by Rambus in 2012) released a 64 Mbit memory in 2010 ^148^ with a 64 Gbit scaling plan based on its CMO_*x*_ technology^5,73^ as an alternative to NAND flash when it reached its scaling limit. Since then, however, NAND flash has not stopped scaling and, to our knowledge, Rambus has not reported further on its CMO_*x*_ memory technology, and signed a license agreement with Western Digital in 2017, which allow them the usage of Rambus resistive memory technology. The application of PCMO-based memory as a storage device could be limited by lower retention (section 4.5.2) compared to other ReRAM technologies.

The only other publicly reported company working on PCMO-based memory is 4DS Memory, founded in 2007. Every year since 2014, they have renewed a joint development agreement with HGST, a subsidiary of Western Digital, which may also give them access to Unity’s memory technology. They have focused on PCMO for DRAM-like applications, since the more reliable area-dependent movement of oxygen vacancies, offers potential advantages in endurance over filamentary devices (see section 4.6). According to the public announcement on their Web site^85^ and on their investor hub to inform the markets and their shareholders, they have produced a megabit array of 60 nm devices in a CMOS fab-compatible process chain at IMEC in Belgium with an endurance of over 10^9^ cycles and a DRAM-competitive write speed of 4.7 ns. This high endurance and switching speed could give 4DS technology a competitive advantage over other ReRAM technologies in DRAM-close applications, as analyzed in a market report paid for by 4DS from Lodge Partners Pty Ltd..^151^

Other companies, such as Micron Technology, have also been researching PCMO as a memory technology, as can be concluded from related patents or publications.^52^ However, there are no other publicly reported industrial activities leading to the manufacture of high-density PCMO-based memory.

## 6 Outlook

Since the first observation of a voltage-induced resistance change in PCMO by Asamitu et al.^152^ in 1997, based on an electronic phase transition at low temperatures, and today’s array integration of PCMO-based ReRAM, hundreds of research papers have been written on PCMO from the material side and on PCMO in switching device stacks.

Fully CMOS integrated arrays based on PCMO-TO stacks could potentially outperform filamentary switching arrays in cycle-to-cycle and read-to-read variability and endurance. The high resistance values of PCMO-based devices, even in the LRS, could enable large crossbar arrays by avoiding the parasitic effects caused by the line resistances that dominate the arrays of their filamentary counterparts. With the ability to provide incremental resistance tuning and associated multilevel states, they could serve as synaptic weights for memristor-based AI hardware accelerators in the growing field of AI technology and neuromorphic computing. New market opportunities for memristors in hardware-based AI accelerators are created by the rapid growth of AI technologies and their excessive need for computational resources and power consumption.^2^ As the current of PCMO-based devices scales with area, it may be possible to achieve femto-joule switching energies for 25 nm Al/Mo/PCMO devices, as discussed by Moon et al.^83^ Low switching energies, together with higher endurance and high switching speed, may be an opportunity for PCMO-based memristor technology for in-memory computing in low-power edge devices.^3^ However, there are still many unanswered questions related to the atomic and electronic processes in the material, as well as the operating and failure mechanisms of the devices. It is still not clear how oxygen vacancies change the electronic transport and how they change the band structure in detail. Extended DFT modeling of the band structure changes could help here. Moreover, there is a lot of room for improvement with respect to the choice of material stacks for improved device performance.

In most current PCMO device stacks, the lack of Joule heating, which enables gradual switching, is accompanied by reduced switching speed, poorer retention and more pronounced read disturbance. This trade-off could be resolved by tailored stacking of materials or interface engineering to confine the field drop to the position where fast ion movement is required. To achieve CMOS integrated crossbar arrays, BEOL compatible PCMO TO stacks are required.

For crystalline PCMO, the research community desperately lacks well-published deposition methods to enable CMOS BEOL integration of PCMO-based devices. On the other hand, the switching of devices based on amorphous PCMO is not yet fully understood. In particular, there is a lack of thorough measurements of the material properties and conductivity of amorphous PCMO. Studies comparing different material combinations could help to gain a systematic understanding.

In order to advance this technology, research into the reliability of PCMO-based devices by analyzing a large number of devices will be required. Today, any analysis of the reliability criteria of PCMO-based devices lacks a statistical basis. This analysis would further contribute to the development of variability-aware compact models that allow reliable circuit simulations in the future.

## References

[1] Aguirre F., Sebastian A., Le Gallo M., Song W., Wang T., Yang J. J., Lu W., Chang M. F., Ielmini D., Yang Y., Mehonic A., Kenyon A., Villena M. A., Roldán J. B., Wu Y., Hsu H. H., Raghavan N., Suñé J., Miranda E., Eltawil A., Setti G., Smagulova K., Salama K. N., Krestinskaya O., Yan X., Ang K. W., Jain S., Li S., Alharbi O., Pazos S., Lanza M. (2024). Hardware Implementation of Memristor-Based
Artificial Neural Networks. Nature Communications
2024 15:1.

[2] Huang Y., Ando T., Sebastian A., Chang M.-F., Yang J. J., Xia Q. (2024). Memristor-Based Hardware
Accelerators for Artificial Intelligence. Nature
Reviews Electrical Engineering 2024 1:5.

[3] Christensen D. V., Dittmann R., Linares-Barranco B., Sebastian A., Le Gallo M., Redaelli A., Slesazeck S., Mikolajick T., Spiga S., Menzel S., Valov I., Milano G., Ricciardi C., Liang S. J., Miao F., Lanza M., Quill T. J., Keene S. T., Salleo A., Grollier J., Marković D., Mizrahi A., Yao P., Yang J. J., Indiveri G., Strachan J. P., Datta S., Vianello E., Valentian A., Feldmann J., Li X., Pernice W. H. P., Bhaskaran H., Furber S., Neftci E., Scherr F., Maass W., Ramaswamy S., Tapson J., Panda P., Kim Y., Tanaka G., Thorpe S., Bartolozzi C., Cleland T. A., Posch C., Liu S. C., Panuccio G., Mahmud M., Mazumder A. N., Hosseini M., Mohsenin T., Donati E., Tolu S., Galeazzi R., Christensen M. E., Holm S., Ielmini D., Pryds N. (2022). 2022 Roadmap on Neuromorphic Computing and Engineering. Neuromorphic Computing and Engineering.

[4] Dittmann R., Menzel S., Waser R. (2021). Nanoionic
Memristive Phenomena in
Metal Oxides: The Valence Change Mechanism. Adv. Phys..

[5] Sawa, A. ; Meyer, R. Interface-Type Switching. In Resistive Switching; Ielmini, D. , Waser, R. , Eds.; John Wiley & Sons, Ltd, 2016; pp 457–482. 10.1002/9783527680870.CH16.

[6] Bagdzevicius S., Maas K., Boudard M., Burriel M., Maas K., Boudard M., Burriel M. (2022). Interface-Type Resistive Switching
in Perovskite Materials. J. Electroceramics.

[7] Schulman A., Huhtinen H., Paturi P. (2024). Manganite
Memristive Devices: Recent
Progress and Emerging Opportunities. J. Phys.
D Appl. Phys..

[8] Lee D., Hwang H., Lee D., Hwang H. (2017). Pr_0.7_Ca_0.3_MnO_3_ (PCMO)-Based
Synaptic Devices. Neuro-inspired Computing Using
Resistive Synaptic Devices.

[9] Pithan C., Iida Y., Dornseiffer J., Tsubouchi A., Waser R. (2022). Oxygen Nonstoichiometry and Electrical
Transport Properties of Pr_1‑x_Ca_x_MnO_3_ Ceramics. J. Eur. Ceram Soc..

[10] Dagotto E., Hotta T., Moreo A. (2001). Colossal Magnetoresistant Materials:
The Key Role of Phase Separation. Phys. Rep..

[11] Hwang H. Y., Cheong S.-W., Radaelli P. G., Marezio M., Batlogg B. (1995). Lattice Effects
on the Magnetoresistance in Doped LaMnO_3_. Phys. Rev. Lett..

[12] Hwang H. Y., Palstra T. T. M., Cheong S. W., Batlogg B. (1995). Pressure Effects
on
the Magnetoresistance in Doped Manganese Perovskites. Phys. Rev. B.

[13] Shannon R. D. (1976). Revised
Effective Ionic Radii and Systematic Studies of Interatomic Distances
in Halides and Chalcogenides. Acta Crystallogr..

[14] Kozakov A. T., Kochur A. G., Trotsenko V. G., Nikolskii A. V., El Marssi M., Gorshunov B. P., Torgashev V. I. (2018). Valence
State of Cations in Manganites Pr_1‑x_Ca_x_MnO_3_ (0.3 ≤ *x* ≤ 0.5) from
X-Ray Diffraction and X-Ray Photoelectron Spectroscopy. J. Alloys Compd..

[15] Anderson P. W., Hasegawa H. (1955). Considerations on Double Exchange. Phys. Rev..

[16] Coey J. M. D., Viret M., Von Molnár S. (1999). Mixed-Valence Manganites. Adv. Phys..

[17] Tomioka Y., Asamitsu A., Kuwahara H., Moritomo Y., Tokura Y. (1996). Magnetic-Field-Induced
Metal-Insulator Phenomena in Pr_1‑x_Ca_x_MnO_3_ with Controlled Charge-Ordering Instability. Phys. Rev. B.

[18] Kressdorf B., Meyer T., Ten Brink M., Seick C., Melles S., Ottinger N., Titze T., Meer H., Weisser A., Hoffmann J., Mathias S., Ulrichs H., Steil D., Seibt M., Blöchl P. E., Jooss C. (2021). Orbital-Order Phase
Transition in Pr_1‑x_Ca_x_MnO_3_ Probed by Photovoltaics. Phys. Rev. B.

[19] Gibbs D., Keimer B., Gog T., Tokura Y., Blume M., Nelson C. S., Hill J. P., Zimmermann M. v., Casa D., Murakami Y., Kao C. C., Venkataraman C., Tomioka Y. (2001). X-Ray Resonant Scattering Studies
of Orbital and Charge
Ordering in Pr_1‑x_Ca_x_MnO_3_. Phys. Rev. B.

[20] Jirák Z., Krupička S., Šimša Z., Dlouhá M., Vratislav S. (1985). Neutron Diffraction Study of Pr_1‑x_Ca_x_MnO_3_ Perovskites. J. Magn Magn Mater..

[21] Ifland B., Peretzki P., Kressdorf B., Saring P., Kelling A., Seibt M., Jooss C. (2015). Current-Voltage
Characteristics of
Manganite-Titanite Perovskite Junctions. Beilstein
J. Nanotechnol.

[22] Schramm S., Hoffmann J., Jooss C. (2008). Transport and Ordering of Polarons
in CER Manganites PrCaMnO. J. Phys.: Condens.
Matter.

[23] Jooss C., Wu L., Beetz T., Klie R. F., Beleggia M., Schofield M. A., Schramm S., Hoffmann J., Zhu Y. (2007). Polaron Melting and
Ordering as Key Mechanisms for Colossal Resistance Effects in Manganites. Proc. Natl. Acad. Sci. U. S. A..

[24] Scherff M., Hoffmann J., Meyer B., Danz T., Jooss C. (2013). Interplay
of Cross-Plane Polaronic Transport and Resistive Switching in Pt-Pr_0.67_Ca_0.33_MnO_3_-Pt Heterostructures. New J. Phys..

[25] Raabe S., Mierwaldt D., Ciston J., Uijttewaal M., Stein H., Hoffmann J., Zhu Y., Blöchl P., Jooss C. (2012). In Situ Electrochemical
Electron Microscopy Study of Oxygen Evolution
Activity of Doped Manganite Perovskites. Adv.
Funct Mater..

[26] Cox, P. A. Transition Metal Oxides. An Introduction to Their Electronic Structure and Properties; Rowlinson, J. S. , Halpern, J. , Green, M. L. H. , Mukaiyama, T. , Eds.; Oxford University Press, 1992; Vol. 105. 10.1002/ANGE.19931050352.

[27] Zampieri G., Abbate M., Prado F., Caneiro A., Morikawa E. (2002). XPS and XAS
Spectra of CaMnO_3_ and LaMnO_3_. Physica B Condens Matter.

[28] Inge S. V., Pandey A., Ganguly U., Bhattacharya A. (2023). Accurate Prediction
of Migration Barrier of Oxygen Vacancy in Formula Presented and Formula
Presented: Explaining Experimental Results with Density Functional
Theory. Phys. Rev. B.

[29] Jung J. H., Kim K. H., Eom D. J., Noh T. W., Choi E. J., Yu J., Kwon Y. S., Chung Y. (1997). Determination of Electronic Band
Structures of CaMnO_3_ and LaMnO_3_ Using Optical-Conductivity
Analyses. Phys. Rev. B.

[30] Lee H. S., Choi S. G., Park H. H., Rozenberg M. J. (2013). A New Route
to the Mott-Hubbard Metal-Insulator Transition: Strong Correlations
Effects in Pr_0.7_Ca_0.3_MnO_3_. Scientific Reports 2013 3:1.

[31] Rini M., Tobey R., Dean N., Itatani J., Tomioka Y., Tokura Y., Schoenlein R. W., Cavalleri A. (2007). Control of
the Electronic Phase of a Manganite by Mode-Selective Vibrational
Excitation. Nature 2007 449:7158.

[32] Asanuma S., Akoh H., Yamada H., Sawa A. (2009). Relationship between
Resistive Switching Characteristics and Band Diagrams of Ti/Pr_1‑x_Ca_x_MnO_3_ Junctions. Phys. Rev. B Condens Matter Mater. Phys..

[33] Sawa A., Fujii T., Kawasaki M., Tokura Y. (2005). Highly Rectifying Pr_0.7_Ca_0.3_MnO_3_/SrTi_0.9998_Nb_0.0002_O_3_ p-n
Junction. Appl.
Phys. Lett..

[34] Heikes, R. R. ; Ure, R. W., Jr. Thermoelectricity: Science and Engineering; Interscience Publishers, Inc., 1961.

[35] Mondal P. S., Asai S., Igarashi T., Suzuki T., Okazaki R., Terasaki I., Yasui Y., Kobayashi K., Kumai R., Nakao H., Murakami Y. (2014). Possible Existence
of Two Charge-Ordered Phases in Pr_1‑x_Ca_x_MnO_3_ for 0.40 ≤ *x* ≤ 0.50. J. Phys. Soc. Jpn..

[36] Yamada S., Arima T. H., Ikeda H., Takita K. (2000). Thermopower in Pr_1‑x_Ca_x_MnO_3_. J. Phys. Soc. Jpn..

[37] Venkataiah G., Kalyana Lakshmi Y., Venugopal Reddy P. (2007). Thermopower Studies of Pr_0.67_D_0.33_MnO_3_ Manganite System. J. Phys.
D Appl. Phys..

[38] Alexandrov A. S., Kornilovitch P. E. (1999). Mobile
Small Polaron. Phys. Rev.
Lett..

[39] Austin I. G., Mott N. F. (1969). Polarons in Crystalline
and Non-Crystalline Materials. Adv. Phys..

[40] Mildner S., Hoffmann J., Blöchl P. E., Techert S., Jooss C. (2015). Temperature-and
Doping-Dependent Optical Absorption in the Small-Polaron System Pr_1‑x_Ca_x_MnO_3_. Phys. Rev. B.

[41] Holstein T. (1959). Studies of
Polaron Motion: Part I. The Molecular-Crystal Model. Ann. Phys. (N Y).

[42] Feinberg D., Ciuchi S., de Pasquale F. (1990). Squeezing
Phenomena in Interacting
Electron-Phonon Systems. Int. J. Mod. Phys.
B.

[43] Holstein T. (2000). Studies of
Polaron Motion: Part II. The “Small” Polaron. Ann. Phys. (N Y).

[44] Hoffmann J, Moschkau P, Mildner S, Norpoth J, Jooss C., Wu L, Zhu Y (2014). Effects of
Interaction and Disorder on Polarons in
Colossal Resistance Manganite Pr_0.68_Ca_0.32_MnO_3_ Thin Films. Mater. Res. Express.

[45] Boris A. V., Kovaleva N. N., Bazhenov A. V., Samoilov A. V., Yeh N. C., Vasquez R. P. (1997). Infrared Optical
Properties of La_0.7_Ca_0.3_MnO_3_ Epitaxial
Films. J.
Appl. Phys..

[46] Herpers, A. Electrical Characterization of Manganite and Titanate Heterostructures. PhD Thesis. Jülich, 2014, Vol. 32. https://juser.fz-juelich.de/record/152080.

[47] Nian Y. B., Strozier J., Wu N. J., Chen X., Ignatiev A. (2007). Evidence for
an Oxygen Diffusion Model for the Electric Pulse Induced Resistance
Change Effect in Transition-Metal Oxides. Phys.
Rev. Lett..

[48] Liao Z., Gao P., Bai X., Chen D., Zhang J. (2012). Evidence for Electric-Field-Driven
Migration and Diffusion of Oxygen Vacancies in Pr_0.7_Ca_0.3_MnO_3_. J. Appl. Phys..

[49] Jooss Ch., Hoffmann J., Fladerer J., Ehrhardt M., Beetz T., Wu L., Zhu Y. (2008). Electric Pulse
Induced Resistance Change Effect in
Manganites Due to Polaron Localization at the Metal-Oxide Interfacial
Region. Phys. Rev. B.

[50] Rene Meyer from Rambus Inc. Tunnel RRAM - Device Features and 1R True Cross-Point Implementation. IFF - FZ Jülich, March 6, 2013.

[51] Reagor D. W., Lee S. Y., Li Y., Jia Q. X. (2004). Work Function
of
the Mixed-Valent Manganese Perovskites. J. Appl.
Phys..

[52] Bi L., Pandey S. C., Ramaswamy N. (2013). Determination of Effective Work Function
of Pr_0.7_Ca_0.3_MnO_3_ and Pt Films on
ZrO_x_ Using Terraced-Oxide Method. Appl. Phys. Lett..

[53] Sawa A., Yamamoto A., Yamada H., Fujii T., Kawasaki M., Matsuno J., Tokura Y. (2007). Fermi Level Shift in La_1‑x_Sr_x_MO_3_ (M = Mn, Fe, Co, and Ni) Probed by Schottky-like
Heteroepitaxial Junctions with SrTi_0.99_Nb_0.01_O_3_. Appl. Phys. Lett..

[54] Herpers A., Lenser C., Park C., Offi F., Borgatti F., Panaccione G., Menzel S., Waser R., Dittmann R. (2014). Spectroscopic
Proof of the Correlation between Redox-State and Charge-Carrier Transport
at the Interface of Resistively Switching Ti/PCMO Devices. Adv. Mater..

[55] Sheng Z., Nakamura M., Kagawa F., Kawasaki M., Tokura Y. (2012). Dynamics of
Multiple Phases in a Colossal-Magnetoresistive Manganite as Revealed
by Dielectric Spectroscopy. Nature Communications
2012 3:1.

[56] Biškup N., De Andrés A., Martinez J. L., Perca C. (2005). Origin of the Colossal
Dielectric Response of Pr_0.6_Ca_0.4_MnO_3_. Phys. Rev. B.

[57] Lee, H.-G. Chemical Thermodynamics for Metals and Materials; World Scientific Publishing Company, 1999.

[58] Liao Z. L., Wang Z. Z., Meng Y., Liu Z. Y., Gao P., Gang J. L., Zhao H. W., Liang X. J., Bai X. D., Chen D. M. (2009). Categorization of Resistive Switching of Metal- Pr_0.7_Ca _0.3_MnO_3_ -Metal Devices. Appl. Phys. Lett..

[59] Shirasaki S., Yamamura H., Haneda H., Kakegawa K., Moori J. (1980). Defect Structure
and Oxygen Diffusion in Undoped and La-doped Polycrystalline Barium
Titanate. J. Chem. Phys..

[60] Gutsche A., Hambsch S., Branca N. C., Dittmann R., Scholz S., Knoch J. (2022). Disentangling Ionic
and Electronic Contributions to the Switching
Dynamics of Memristive Pr_0.7_Ca_0.3_MnO_3_/Al Devices by Employing a Two-Resistor Model. Phys. Rev. Mater..

[61] Shono K., Kawano H., Yokota T., Gomi M. (2008). Origin of Negative
Differential Resistance Observed on Bipolar Resistance Switching Device
with Ti/Pr_0.7_Ca_0.3_MnO_3_/Pt Structure. Applied Physics Express.

[62] Gutsche A., Siegel S., Zhang J., Hambsch S., Dittmann R. (2021). Exploring
Area-Dependent Pr_0.7_Ca_0.3_MnO_3_-Based
Memristive Devices as Synapses in Spiking and Artificial Neural Networks. Front Neurosci.

[63] Kaji H., Kondo H., Fujii T., Arita M., Takahashi Y. (2010). Effect of
Electrode and Interface Oxide on the Property of ReRAM Composed of
Pr_0.7_Ca_0.3_MnO_3_. IOP Conf Ser. Mater. Sci. Eng..

[64] Gutsche, A. Area-Dependent Resistive Switching in PCMO-Based Memristive Devices, PhD Dissertation, Aachen, 2023. 10.18154/RWTH-2023-07951.

[65] Moon, K. ; Park, S. ; Lee, D. ; Woo, J. ; Cha, E. ; Lee, S. ; Hwang, H. Resistive-Switching Analogue Memory Device for Neuromorphic Application. In 2014 Silicon Nanoelectronics Workshop, SNW 2014; IEEE, 2015 10.1109/SNW.2014.7348602.

[66] Baek K., Park S., Park J., Kim Y.-M., Hwang H., Oh S. H. (2017). In Situ TEM Observation on the Interface-Type Resistive Switching
by Electrochemical Redox Reactions at a TiN/PCMO Interface. Nanoscale.

[67] Smoliner, J. Grundlagen Der Halbleiterphysik, 2nd ed.; Springer Berlin Heidelberg, 2020. 10.1007/978-3-662-60654-4.

[68] Ifland B., Peretzki P., Kressdorf B., Saring P., Kelling A., Seibt M., Jooss C. (2015). Current-Voltage
Characteristics of
Manganite-Titanite Perovskite Junctions. Beilstein
Journal of Nanotechnology 6:152.

[69] Seibt, M. ; Jooß, C. ; Blöchl, P. ; Hofsäss, H.-C. ; Ropers, C. ; Volkert, C. A. Structural and Electronic Investigation of Strongly Correlated Transition Metal Oxide Perovskite Thin Films and Interfaces Using In-Situ Transmission Electron Microscopy. Doctoral Thesis, 2020. 10.53846/GOEDISS-8815.

[70] Saucke G., Norpoth J., Jooss C., Su D., Zhu Y. (2012). Polaron Absorption
for Photovoltaic Energy Conversion in a Manganite-Titanate Pn Heterojunction. Phys. Rev. B Condens Matter Mater. Phys..

[71] Meyer T., Kressdorf B., Lindner J., Peretzki P., Roddatis V., Jooss C., Seibt M. (2019). High-Resolution Scanning Transmission
EBIC Analysis of Misfit Dislocations at Perovskite Pn-Heterojunctions. IOP Publishing.

[72] Borgatti F., Park C., Herpers A., Offi F., Egoavil R., Yamashita Y., Yang A., Kobata M., Kobayashi K., Verbeeck J., Panaccione G., Dittmann R. (2013). Chemical Insight into
Electroforming of Resistive Switching Manganite Heterostructures. Nanoscale.

[73] Meyer, R. ; Schloss, L. ; Brewer, J. ; Lambertson, R. ; Kinney, W. ; Sanchez, J. ; Rinerson, D. Oxide Dual-Layer Memory Element for Scalable Non-Volatile Cross-Point Memory Technology. 9th Annual Non-Volatile Memory Technology Symposium, NVMTS; IEEE, 2008; pp 1–5 10.1109/NVMT.2008.4731194

[74] Arndt B., Borgatti F., Offi F., Phillips M., Parreira P., Meiners T., Menzel S., Skaja K., Panaccione G., MacLaren D. A., Waser R., Dittmann R. (2017). Spectroscopic Indications
of Tunnel Barrier Charging as the Switching Mechanism in Memristive
Devices. Adv. Funct Mater..

[75] Simmons J. G. (1963). Electric
Tunnel Effect between Dissimilar Electrodes Separated by a Thin Insulating
Film. J. Appl. Phys..

[76] Funck C., Menzel S. (2021). Comprehensive Model
of Electron Conduction in Oxide-Based
Memristive Devices. ACS Appl. Electron Mater..

[77] Houng M. P., Wang Y. H., Chang W. J. (1999). Current Transport Mechanism in Trapped
Oxides: A Generalized Trap-Assisted Tunneling Model. J. Appl. Phys..

[78] Lashkare S., Chouhan S., Chavan T., Bhat A., Kumbhare P., Ganguly U. (2018). PCMO RRAM for Integrate-and-Fire
Neuron in Spiking
Neural Networks. IEEE Electron Device Lett..

[79] Chakraborty, I. ; Panwar, N. ; Khanna, A. ; Ganguly, U. Space Charge Limited Current with Self-Heating in Pr_0.7_Ca_0.3_MnO_3_ Based RRAM. arXiv 2016, 10.48550/arXiv.1605.08755.

[80] Chakraborty, I. ; Singh, A. K. ; Kumbhare, P. ; Panwar, N. ; Ganguly, U. Materials Parameter Extraction Using Analytical Models in PCMO Based RRAM. In Device Research Conference - Conference Digest, DRC; Institute of Electrical and Electronics Engineers Inc., 2015; Vol. 2015-August, pp 87–88. 10.1109/DRC.2015.7175568.

[81] Fujimoto M., Koyama H., Nishi Y., Suzuki T. (2007). Resistive Switching
Properties of High Crystallinity and Low-Resistance Pr_0.7_Ca_0.3_MnO_3_ Thin Film with Point-Contacted Ag
Electrodes. Appl. Phys. Lett..

[82] Harada T., Ohkubo I., Tsubouchi K., Kumigashira H., Ohnishi T., Lippmaa M., Matsumoto Y., Koinuma H., Oshima M. (2008). Trap-Controlled Space-Charge-Limited
Current Mechanism in Resistance Switching at Al/Pr_0.7_Ca_0.3_MnO_3_ Interface. Appl. Phys.
Lett..

[83] Moon K., Fumarola A., Sidler S., Jang J., Narayanan P., Shelby R. M., Burr G. W., Hwang H. (2018). Bidirectional Non-Filamentary
RRAM as an Analog Neuromorphic Synapse, Part I: Al/Mo/Pr_0.7_Ca_0.3_MnO_3_ Material Improvements and Device
Measurements. IEEE Journal of the Electron Devices
Society.

[84] Chiou S.-Y., Lin M.-H., Park S., Siddik M., Noh J., Lee D., moon K., Woo J., Hun Lee B., Hwang H. (2014). A Nitrogen-Treated
Memristive Device for Tunable Electronic Synapses. Semicond. Sci. Technol..

[85] 4DS Memory Limited - Website. https://www.4dsmemory.com/ (accessed 2025-03-05).

[86] Linderälv C., Lindman A., Erhart P. (2018). A Unifying Perspective on Oxygen
Vacancies in Wide Band Gap Oxides. J. Phys.
Chem. Lett..

[87] Arndt, B. J. ; Waser, R. ; Mayer, J. Resistive Switching in Pr_1‑x_Ca_x_MnO_3_YSZ: Conclusive Model and Switching Kinetics. Doctoral thesis, 2022. 10.18154/RWTH-2022-02791.

[88] Lackner P., Zou Z., Mayr S., Diebold U., Schmid M. (2019). Using Photoelectron
Spectroscopy to Observe Oxygen Spillover to Zirconia. Phys. Chem. Chem. Phys..

[89] Fabris S., Paxton A. T., Finnis M. W. (2002). A Stabilization
Mechanism of Zirconia
Based on Oxygen Vacancies Only. Acta Mater..

[90] Jiang H., Gomez-Abal R. I., Rinke P., Scheffler M. (2010). Electronic
Band Structure of Zirconia and Hafnia Polymorphs from the GW Perspective. Phys. Rev. B.

[91] Götsch T., Bertel E., Menzel A., Stöger-Pollach M., Penner S. (2018). Spectroscopic Investigation of the Electronic Structure
of Yttria-Stabilized Zirconia. Phys. Rev. Mater..

[92] McComb D. W. (1996). Bonding
and Electronic Structure in Zirconia Pseudopolymorphs Investigated
by Electron Energy-Loss Spectroscopy. Phys.
Rev. B.

[93] Ostanin S., Craven A. J., McComb D. W., Vlachos D., Alavi A., Finnis M. W., Paxton A. T. (2000). Effect of Relaxation
on the Oxygen
K-Edge Electron Energy-Loss near-Edge Structure in Yttria-Stabilized
Zirconia. Phys. Rev. B.

[94] Sommer N., Dittmann R., Menzel S. (2023). Effect of
Oxygen Exchange between
Two Oxide Layers of a Memristive Bilayer Valence-Change Memory Cell
on the Switching Polarity. Phys. Rev. Appl..

[95] Scherff M., Meyer B.-U., Hoffmann J., Jooss Ch. (2011). Polarity Reversal
in Bipolar Resistive Switching in Pr_0.7_Ca_0.3_MnO_3_ Noble Metal Sandwich Structures. J. Appl. Phys..

[96] Kramer T., Mierwaldt D., Scherff M., Kanbach M., Jooss C. (2018). Developing
an in Situ Environmental TEM Set up for Investigations of Resistive
Switching Mechanisms in Pt-Pr_1‑x_Ca_x_MnO_3‑δ_-Pt Sandwich Structures. Ultramicroscopy.

[97] Saraswat V., Prasad S., Khanna A., Wagh A., Bhat A., Panwar N., Lashkare S., Ganguly U. (2020). Reaction-Drift Model
for Switching Transients in Pr_0.7_Ca_0.3_MnO_3_-Based Resistive RAM. IEEE Trans. Electron
Devices.

[98] Sawa A. (2008). Resistive
Switching in Transition Metal Oxides. Mater.
Today.

[99] Menzel S., Böttger U., Wimmer M., Salinga M. (2015). Physics of
the Switching
Kinetics in Resistive Memories. Adv. Funct Mater..

[100] Buczek M., Pohlmann M., Liu Z., Moos Z., Gutsche A., Cao P., Mayer J., Stein W., Dittmann R. (2024). Large Area Pulsed Laser
Deposition of Memristive Pr_0.7_Ca_0.3_MnO_3_ Heterostructures for Neuromorphic
Computing. Thin Solid Films.

[101] Zhang T., Bai Y., Jia C.-H., Zhang W.-F. (2012). Interface-Related
Switching Behaviors of Amorphous Pr_0.67_Sr_0.33_MnO_3_-Based Memory Cells. Chinese
Physics B.

[102] Kanegami N., Nishi Y., Kimoto T. (2020). Unique Resistive Switching
Phenomena Exhibiting Both Filament-Type and Interface-Type Switching
in Ti/Pr_0.7_Ca_0.3_MnO_3‑δ_/Pt ReRAM Cells. Appl. Phys. Lett..

[103] Seong T. G., Choi K. B., Lee B. S., Kim B. Y., Oh J. H., Jung K. H., Hong K., Nahm S. (2013). Effect of
Oxygen Pressure on the Resistive Switching Behavior of Amorphous Pr_0.7_Ca_0.3_MnO_3_ Films. ECS Solid State Letters.

[104] Yamamoto H., Murakami T., Sakai J., Imai S. (2007). Correlation
between Degree of Crystallinity and Transition Field in Electric or
Magnetic Field-Induced Insulator-Metal Transition of Pr_0.5_Ca_0.5_MnO_3_ Thin Films. Solid State Commun..

[105] Seong T. G., Lee B. S., Choi K. B., Kweon S. H., Kim B. Y., Jung K., Moon J. W., Lee K. J., Hong K., Nahm S. (2014). Unipolar Resistive Switching Properties
of Amorphous Pr_0.7_Ca_0.3_MnO_3_ Films
Grown on a Pt/Ti/SiO_2_/Si Substrate. Curr. Appl. Phys..

[106] Zhang R., Miao J., Shao F., Huang W. T., Dong C., Xu X. G., Jiang Y. (2014). Transparent Amorphous
Memory Cell: A Bipolar Resistive Switching in ZnO/Pr_0.7_Ca_0.3_MnO_3_/ITO for Invisible Electronics Application. J. Non Cryst. Solids.

[107] Seong T. G., Bum Choi K., Seo I. T., Oh J. H., Won Moon J., Hong K., Nahm S. (2012). Resistive
Switching
Properties of Amorphous Pr_0.7_Ca _0.3_MnO_3_ Films Grown on Indium Tin Oxide/Glass Substrate Using Pulsed Laser
Deposition Method. Appl. Phys. Lett..

[108] Lee D., Moon K., Park J., Park S., Hwang H. (2015). Trade-off
between Number of Conductance States and Variability of Conductance
Change in Pr_0.7_Ca_0.3_MnO_3_-Based Synapse
Device. Appl. Phys. Lett..

[109] De Souza R. A. (2016). Ion Transport in Metal Oxides. Resistive Switching.

[110] Wahl A., Hardy V., Martin C., Simon Ch. (2002). Magnetic
Contributions to the Low-Temperature Specific Heat of the Ferromagnetic
Insulator Pr_0.8_Ca_0.2_MnO_3_. European Physical Journal B - Condensed Matter and Complex
Systems.

[111] Waser R., Dittmann R., Staikov C., Szot K. (2009). Redox-Based
Resistive Switching Memories - Nanoionic Mechanisms, Prospects, and
Challenges. Adv. Mater..

[112] Dittmann, R. ; Sarantopoulos, A. ; Bengel, C. ; Gutsche, A. ; Cüppers, F. ; Hoffmann-Eifert, S. ; Menzel, S. Engineering the Kinetics of Redox-Based Memristive Devices for Neuromorphic Computing. In International Electron Devices Meeting (IEDM); IEEE: San Francisco, CA, USA, 2023. 10.1109/IEDM45741.2023.10413803.

[113] Panwar N., Khanna A., Kumbhare P., Chakraborty I., Ganguly U. (2017). Self-Heating during Submicrosecond
Current Transients
in Pr_0.7_Ca_0.3_MnO_3_-Based RRAM. IEEE Trans. Electron Devices.

[114] Moon K., Cha E., Park J., Gi S., Chu M., Baek K., Lee B., Oh S. H., Hwang H. (2016). Analog Synapse
Device with 5-b MLC and Improved Data Retention for Neuromorphic System. IEEE Electron Device Lett..

[115] Kumbhare P., Ganguly U. (2018). Ionic Transport Barrier
Tuning by
Composition in Pr_1‑x_Ca_x_MnO_3_-Based Selector-Less RRAM and Its Effect on Memory Performance. IEEE Trans. Electron Devices.

[116] Lee, C. ; Kwak, M. ; Choi, W. K. ; Kim, S. ; Hwang, H. Improved On-Chip Training Efficiency at Elevated Temperature and Excellent Inference Accuracy with Retention (> 10^8^s) of Pr_0.7_Ca_0.3_MnO_3‑x_ ECRAM Synapse Device for Hardware Neural Network. In Technical Digest - International Electron Devices Meeting, IEDM; IEEE, 2021; 2021-December, pp 12.3.1–12.3.4. 10.1109/IEDM19574.2021.9720597.

[117] Inge, S. V. ; Pandey, A. ; Ganguly, U. ; Bhattacharya, A. Understanding and Predicting the Activation Energy of Oxygen Migration in Pr_0.5_Ca_0.5_MnO: A DFT Study. In IEEE Electron Devices Technology and Manufacturing Conference: Strengthening the Globalization in Semiconductors, EDTM 2024; IEEE, 2024. 10.1109/EDTM58488.2024.10511856.

[118] Wiefels S., Kopperberg N., Hofmann K., Otterstedt J., Wouters D., Waser R., Menzel S. (2024). Reliability Aspects
of 28 Nm BEOL-Integrated Resistive Switching Random Access Memory. Physica Status Solidi (A) Applications and Materials Science.

[119] Wouters, D. J. ; Chen, Y.-Y. ; Fantini, A. ; Raghavan, N. Reliability Aspects. In Resistive Switching; John Wiley & Sons, Ltd, 2016; pp 597–622. 10.1002/9783527680870.ch21.

[120] Lanza M., Molas G., Naveh I. (2023). The Gap between Academia
and Industry in Resistive Switching Research. Nature Electronics 2023 6:4.

[121] Lanza M., Waser R., Ielmini D., Yang J. J., Goux L., Suñe J., Kenyon A. J., Mehonic A., Spiga S., Rana V., Wiefels S., Menzel S., Valov I., Villena M. A., Miranda E., Jing X., Campabadal F., Gonzalez M. B., Aguirre F., Palumbo F., Zhu K., Roldan J. B., Puglisi F. M., Larcher L., Hou T. H., Prodromakis T., Yang Y., Huang P., Wan T., Chai Y., Pey K. L., Raghavan N., Dueñas S., Wang T., Xia Q., Pazos S. (2021). Standards for the Characterization
of Endurance in Resistive Switching Devices. ACS Nano.

[122] Park, S. ; Sheri, A. ; Kim, J. ; Noh, J. ; Jang, J. ; Jeon, M. ; Lee, B. ; Lee, B. R. ; Lee, B. H. ; Hwang, H. Neuromorphic Speech Systems Using Advanced ReRAM-Based Synapse. In International Electron Devices Meeting; IEEE: Washington, DC, USA, 2013; pp 25.6.1–25.6.4. 10.1109/IEDM.2013.6724692.

[123] Liao Z., Gao P., Meng Y., Zhao H., Bai X., Zhang J., Chen D. (2011). Electroforming and Endurance Behavior
of Al/Pr_0.7_Ca _0.3_MnO_3_/Pt Devices. Appl. Phys. Lett..

[124] Panwar, N. ; Ganguly, U. Variability Assessment and Mitigation by Predictive Programming in Pr_0.7_Ca_0.3_MnO_3_ Based RRAM. In Device Research Conference - Conference Digest, DRC; Institute of Electrical and Electronics Engineers Inc., 2015; Vol. 2015-August, pp 141–142. 10.1109/DRC.2015.7175595.

[125] Phadke O., Saraswat V., Ganguly U. (2022). Highly Deterministic
One-Shot Set-Reset Programming Scheme in PCMO Resistive Random-Access
Memory. ACS Appl. Electron Mater..

[126] Kumbhare P., Chakraborty I., Khanna A., Ganguly U. (2017). Memory Performance
of a Simple Pr_0.7_Ca_0.3_MnO_3_-Based
Selectorless RRAM. IEEE Trans. Electron Devices.

[127] Lee M. S., Lee J. K., Hwang H. S., Shin H. C., Park B. G., Park Y. J., Lee J. H. (2011). Conduction Mechanism
and Low Frequency Noise Analysis in Al/Pr _0.7_Ca_0.3_MnO_3_ for Bipolar Resistive Switching. Jpn. J. Appl. Phys..

[128] Cho S., Lee C., Lee D. (2024). Synapse Device Based Neuromorphic
System for Biomedical Applications. Biomed Eng.
Lett..

[129] Hong E., Jeon S., Kim N., Kim H. W., Kang H., Moon K., Woo J. (2023). Convolutional Kernel
with PrCaMnO_x_-Based Resistive Random-Access Memory for
Neuromorphic Image Processing. AIP Adv..

[130] Park, S. ; Kim, H. ; Choo, M. ; Noh, J. ; Sheri, A. ; Jung, S. ; Seo, K. ; Park, J. ; Kim, S. ; Lee, W. ; Shin, J. ; Lee, D. ; Choi, G. ; Woo, J. ; Cha, E. ; Jang, J. ; Park, C. ; Jeon, M. ; Lee, B. ; Lee, B. H. ; Hwang, H. RRAM-Based Synapse for Neuromorphic System with Pattern Recognition Function. In International Electron Devices Meeting; IEEE: San Francisco, CA, USA, 2012; pp 10.2.1–10.2.4. 10.1109/IEDM.2012.6479016.

[131] Schuman C. D., Kulkarni S. R., Parsa M., Mitchell J. P., Date P., Kay B. (2022). Opportunities for Neuromorphic
Computing
Algorithms and Applications. Nat. Comput. Sci..

[132] Xia Q., Yang J. J. (2019). Memristive Crossbar Arrays for Brain-Inspired Computing. Nat. Mater..

[133] Baltaci S. B., Mogulkoc R., Baltaci A. K. (2019). Molecular Mechanisms
of Early and Late LTP. Neurochem. Res..

[134] Park S., Chu M., Kim J., Noh J., Jeon M., Hun Lee B., Hwang H., Lee B., Lee B. G. (2015). Electronic System
with Memristive Synapses for Pattern
Recognition. Sci. Rep.

[135] Lee C., Koo S. M., Oh J. M., Lee D. (2018). Compensated Synaptic
Device for Improved Recognition Accuracy of Neuromorphic System. IEEE Journal of the Electron Devices Society.

[136] Lee J. E., Lee C., Kim D. W., Lee D., Seo Y. H. (2020). An On-Chip Learning Method for Neuromorphic Systems
Based on Non-Ideal Synapse Devices. Electronics
(Basel).

[137] Sheri A. M., Hwang H., Jeon M., Lee B. G. (2014). Neuromorphic
Character Recognition System with Two PCMO Memristors as a Synapse. IEEE Transactions on Industrial Electronics.

[138] Gao, Y. ; Wu, S. ; Adam, G. C. Batch Training for Neuromorphic Systems with Device Non-Idealities. In ACM International Conference Proceeding Series; ACM, 2020. 10.1145/3407197.3407208

[139] Chicca E., Indiveri G. (2020). A Recipe for Creating Ideal Hybrid
Memristive-CMOS Neuromorphic Processing Systems. Appl. Phys. Lett..

[140] Shukla, A. ; Prasad, S. ; Lashkare, S. ; Ganguly, U. A Case for Multiple and Parallel RRAMs as Synaptic Model for Training SNNs. In International Joint Conference on Neural Networks (IJCNN); Institute of Electrical and Electronics Engineers Inc.: Rio de Janeiro, Brazil, 2018; Vol. 2018-July, pp 1–8. 10.1109/IJCNN.2018.8489429.

[141] Chu M., Kim B., Park S., Hwang H., Jeon M., Lee B. H., Lee B. G. (2015). Neuromorphic Hardware
System for
Visual Pattern Recognition with Memristor Array and CMOS Neuron. IEEE Transactions on Industrial Electronics.

[142] Panwar N., Kumbhare P., Singh A. K., Venkataramani N., Ganguly U. (2015). Effect of Morphological Change on Unipolar and Bipolar
Switching Characteristics in Pr_0.7_Ca_0.3_MnO_3_ Based RRAM. Mater. Res. Soc. Symp.
Proc..

[143] Gang J. L., Li S. L., Liao Z. L., Meng Y., Liang X. J., Chen D. M. (2010). Clockwise vs Counter-Clockwise I-V
Hysteresis of Point-Contact Metal-Tip/Pr_0.7_Ca_0.3_MnO_3_/Pt Devices. Chin. Phys. Lett..

[144] Kim D. S., Kim Y. H., Lee C. E., Kim Y. T. (2006). Resistive
Switching Characteristics of Pr_0.7_Ca_0.3_MnO_3_ Thin Films Grown on Glass Substrates by Pulsed Laser Deposition. Thin Solid Films.

[145] Lee J., Jo M., Seong D. J., Shin J., Hwang H. (2011). Materials
and Process Aspect of Cross-Point RRAM (Invited). Microelectron. Eng..

[146] Jo M., Seong D. J., Kim S., Lee J., Lee W., Park J. B., Park S., Jung S., Shin J., Lee D., Hwang H. (2010). Novel Cross-Point Resistive
Switching Memory with Self-Formed
Schottky Barrier. Digest of Technical Papers
- Symposium on VLSI Technology.

[147] Park S., Jung S., Siddik M., Jo M., Park J., Kim S., Lee W., Shin J., Lee D., Choi G., Woo J., Cha E., Lee B. H., Hwang H. (2012). Self-Formed Schottky Barrier Induced Selector-Less RRAM for Cross-Point
Memory Applications. Physica Status Solidi -
Rapid Research Letters.

[148] Chevallier, C. J. ; Siau, C. H. ; Lim, S. F. ; Namala, S. R. ; Matsuoka, M. ; Bateman, B. L. ; Rinerson, D. A 0.13μm 64Mb Multi-Layered Conductive Metal-Oxide Memory Dig Tech Pap IEEE Int. Solid State Circuits Conf; IEEE, 2010; Vol. 53, pp 260–261. 10.1109/ISSCC.2010.5433945.

[149] Lee H. S., Park H. H., Rozenberg M. J. (2015). Manganite-Based
Memristive Heterojunction with Tunable Non-Linear I - V Characteristics. Nanoscale.

[150] Lashkare S., Panwar N., Kumbhare P., Das B., Ganguly U. (2017). PCMO-Based RRAM and NPN Bipolar Selector as Synapse
for Energy Efficient STDP. IEEE Electron Device
Lett..

[151] Sven Restel, L. P. P. L. Initiation Research Report 4DS Memory Ltd (ASX:4DS) New Emerging Memory; 2024. https://www.4dsmemory.com/ (accessed 2025-03-05).

[152] Asamitsu A., Tomioka Y., Kuwahara H., Tokura Y. (1997). Current Switching
of Resistive States in Magnetoresistive Manganites. Nature 1997 388:6637.

